# Supplementary figures and images for: Spatiotemporal recruitment of the ubiquitin-specific protease USP8 directs endosome maturation
Source: eLife. 2024 Nov 22;13:RP96353. doi: 10.7554/eLife.96353 (PMC11584181; doi:10.7554/eLife.96353)

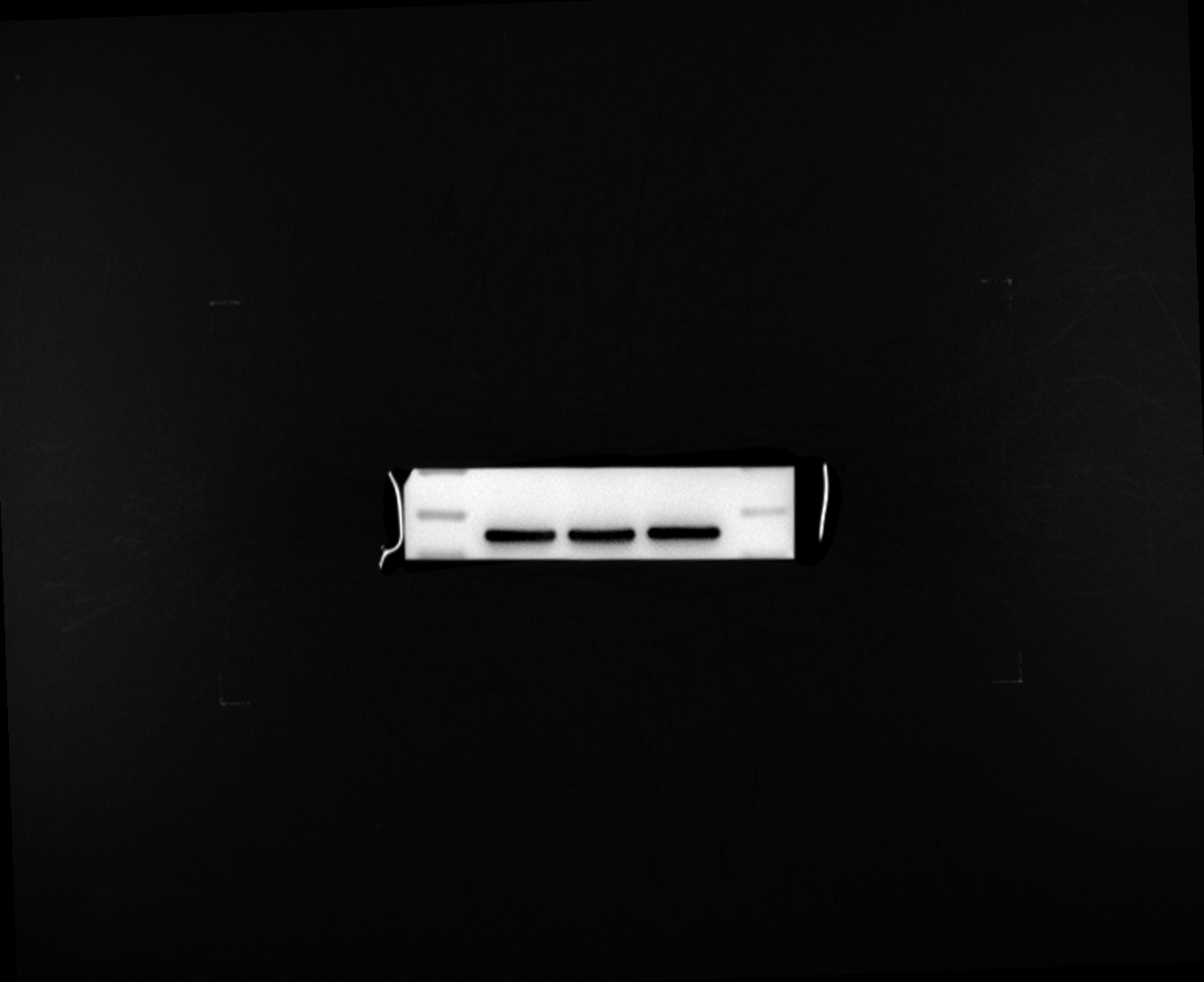

Supplement: Figure 1—figure supplement 1—source data 1. [file elife-96353-fig1-figsupp1-data1.zip › fig. 2-S1C/4-GAPDH-0.5S+M-1(fig2-s1).tif]

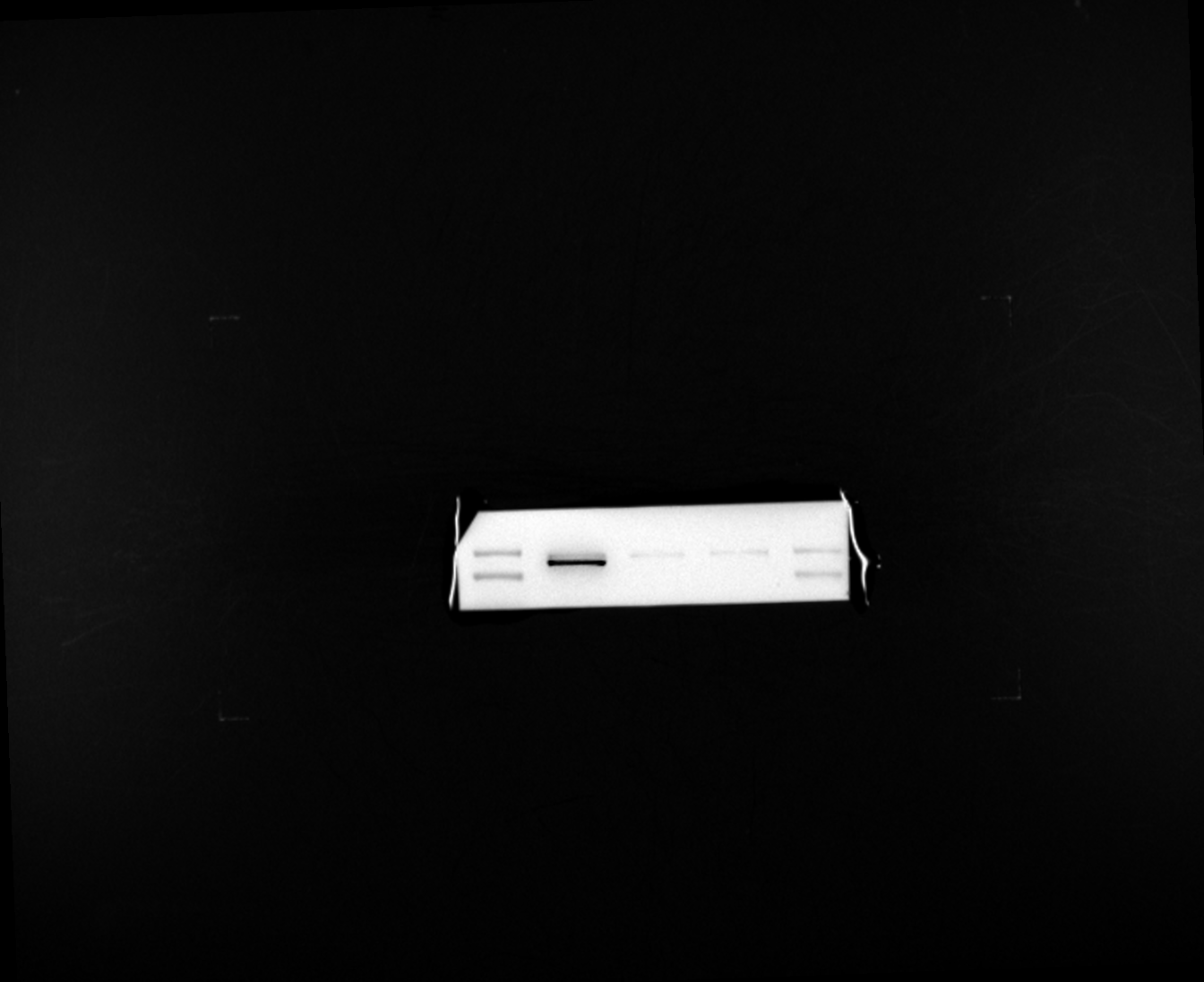

Supplement: Figure 1—figure supplement 1—source data 1. [file elife-96353-fig1-figsupp1-data1.zip › fig. 2-S1C/4-UBPY-5S+M-1(fig2-s1).tif]

**Figure 2-S1C**

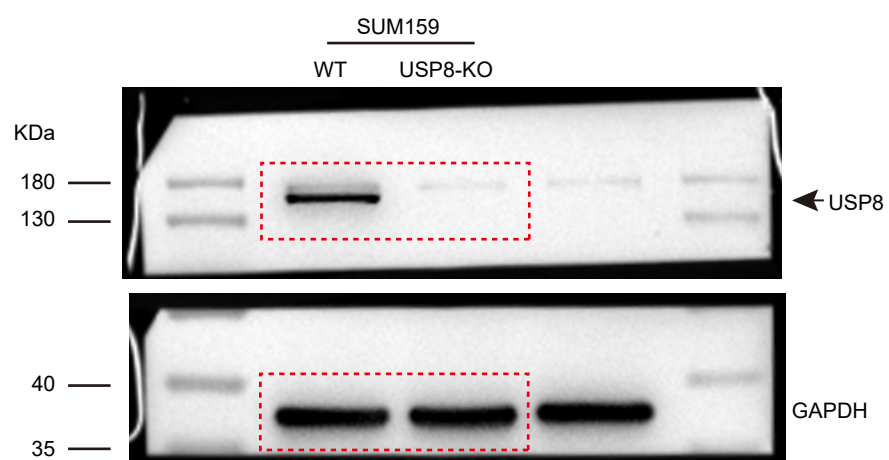

Supplement: Figure 2—figure supplement 1—source data 2. [file elife-96353-fig2-figsupp1-data2.pdf]

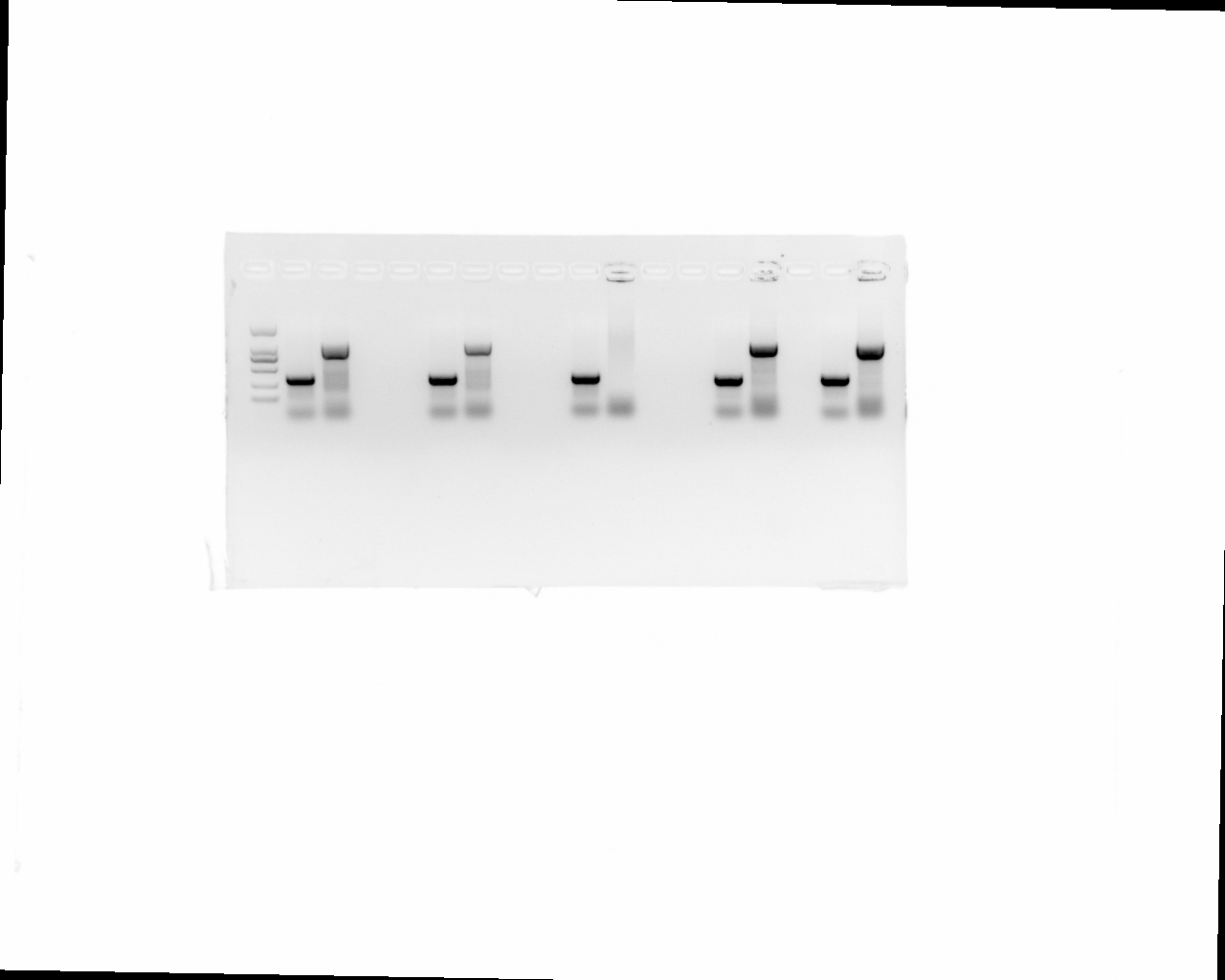

Supplement: Figure 3—figure supplement 2—source data 1. [file elife-96353-fig3-figsupp2-data1.zip › fig. 3-S2B/USP8-GFP-C2---2-1(fig3-s2).tif]

Figure 3-S2B

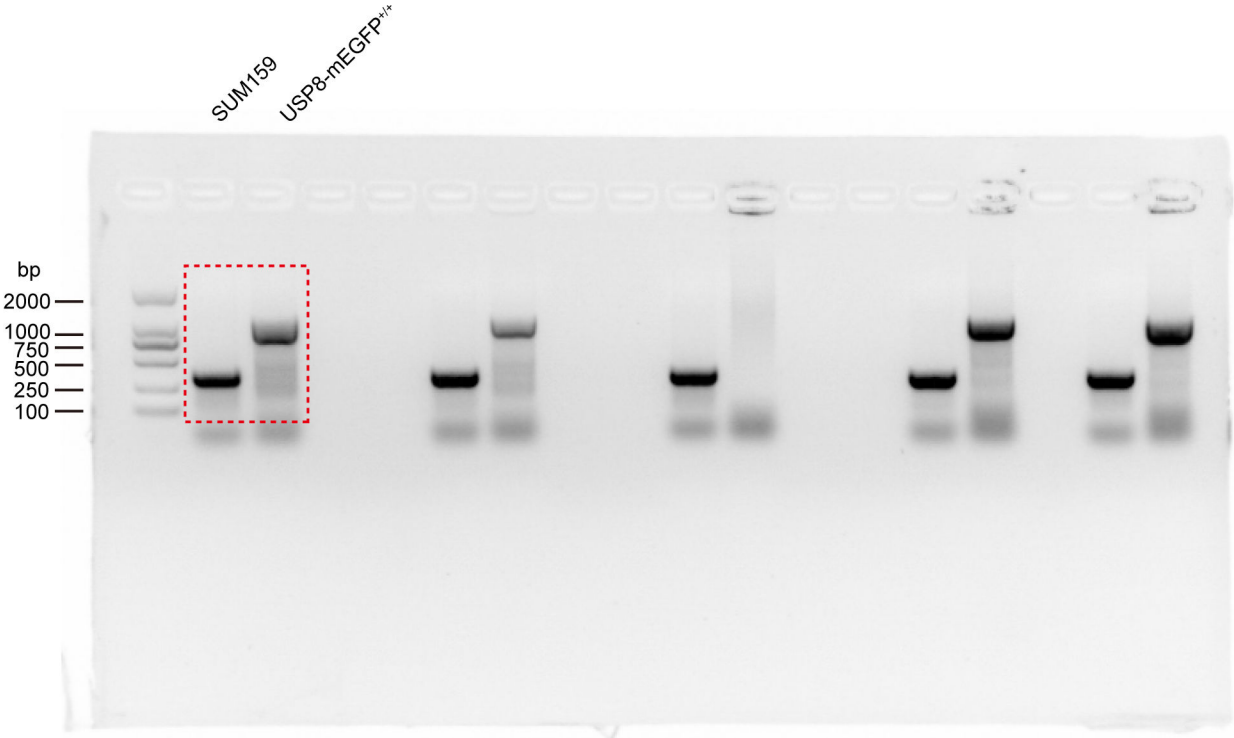

Supplement: Figure 3—figure supplement 2—source data 2. [file elife-96353-fig3-figsupp2-data2.pdf]

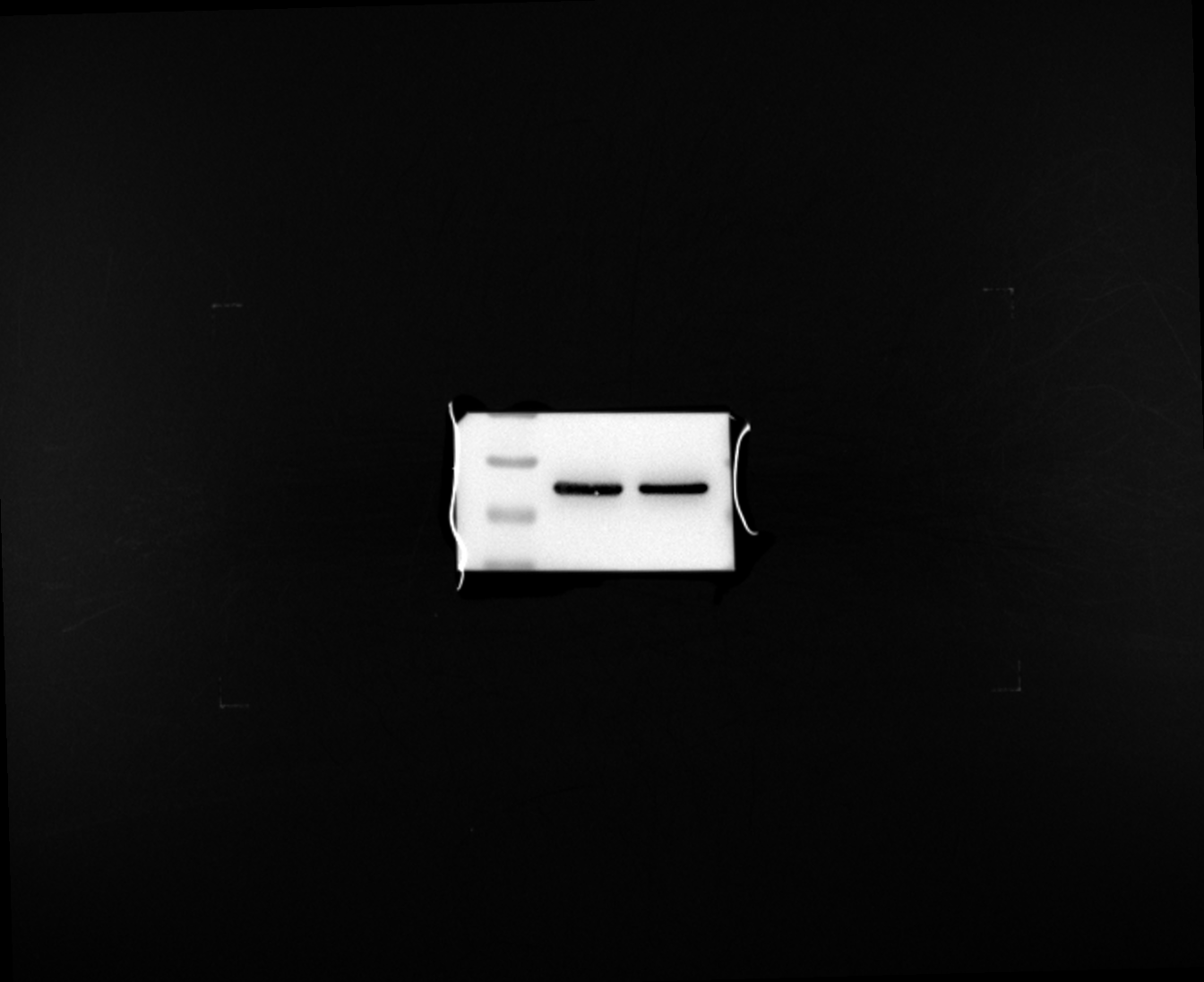

Supplement: Figure 3—figure supplement 2—source data 3. [file elife-96353-fig3-figsupp2-data3.zip › fig. 3-S2C/GAPDH-2S+M-1(fig3-s2).tif]

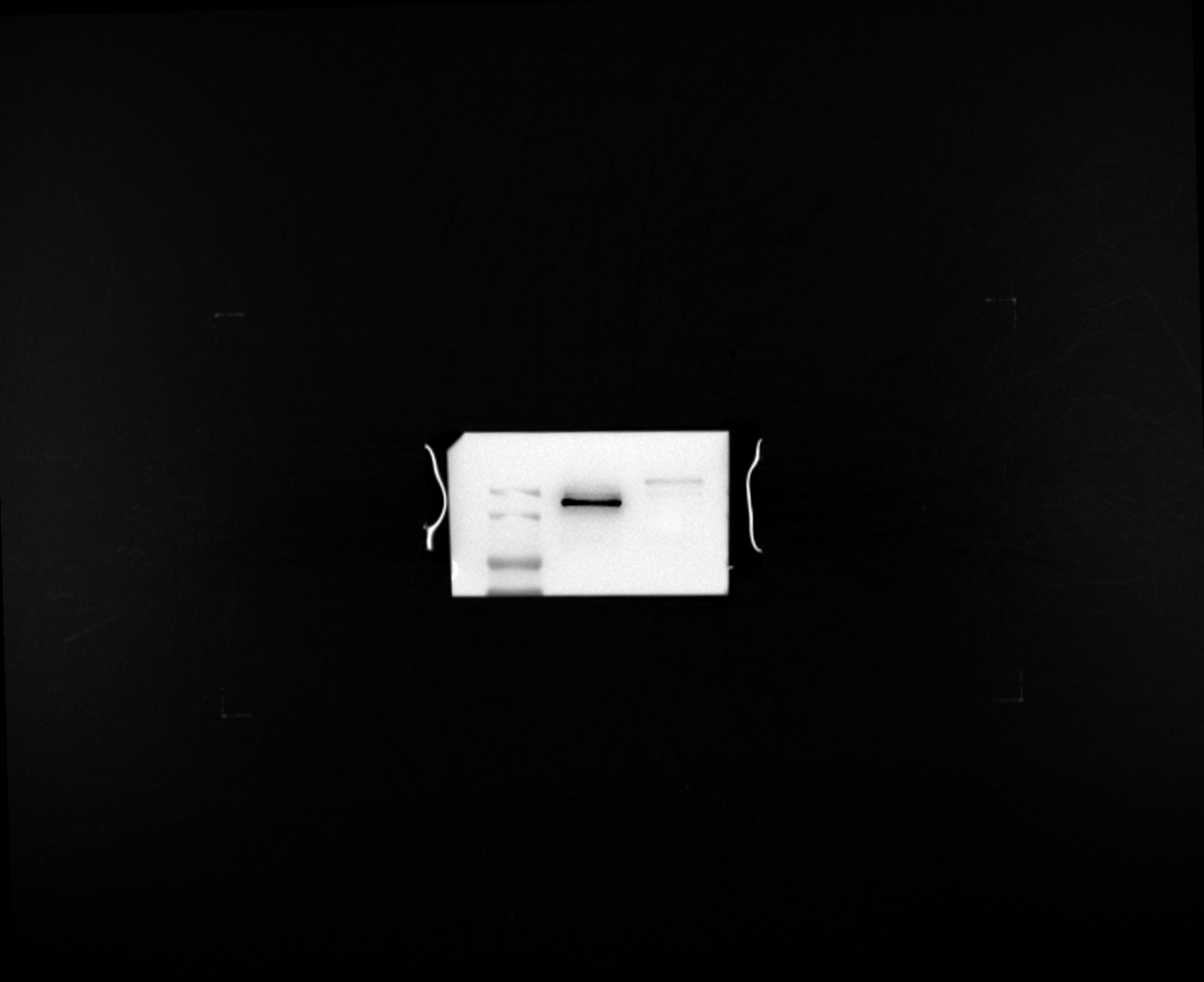

Supplement: Figure 3—figure supplement 2—source data 3. [file elife-96353-fig3-figsupp2-data3.zip › fig. 3-S2C/usp8-4s+m-1(fig3-s2).tif]

**Figure 3-S2C**

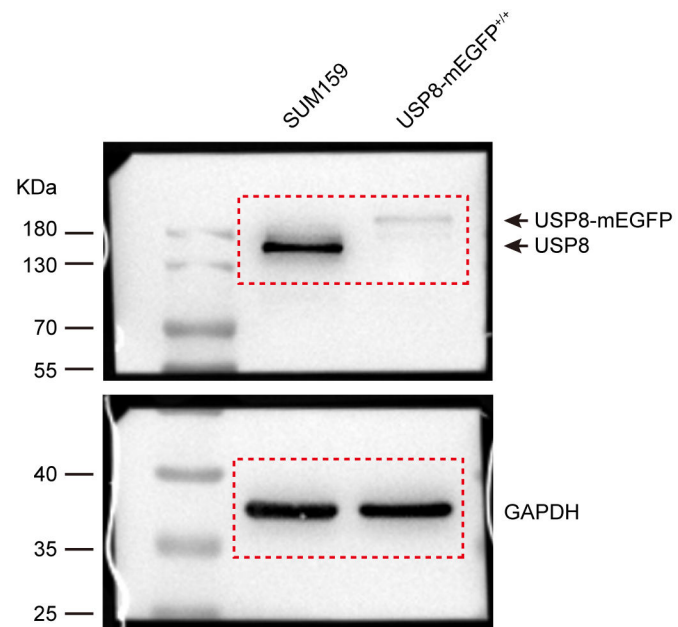

Supplement: Figure 3—figure supplement 2—source data 4. [file elife-96353-fig3-figsupp2-data4.pdf]

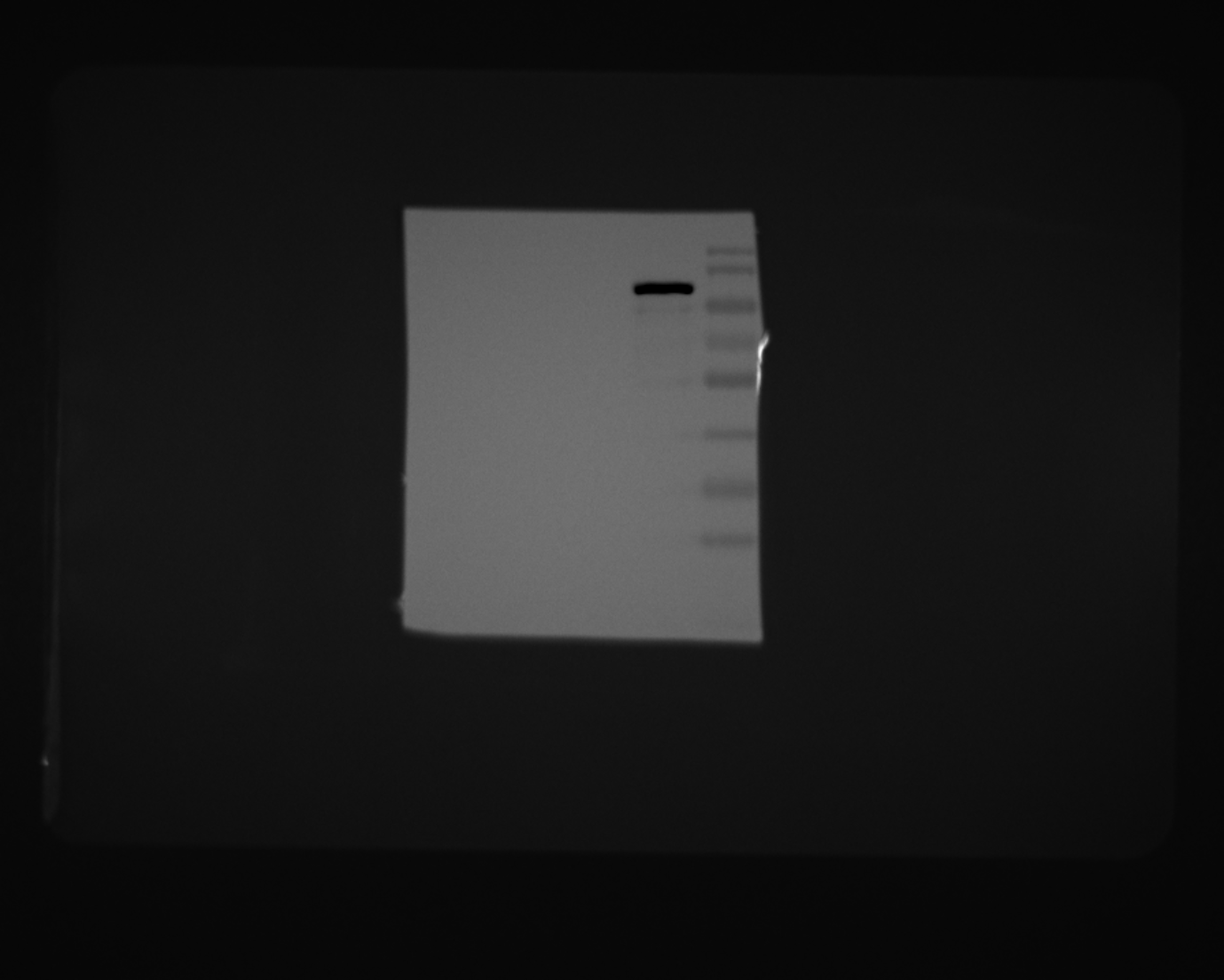

Supplement: Figure 4—source data 1. [file elife-96353-fig4-data1.zip › fig. 4A/input anti-flag.tif]

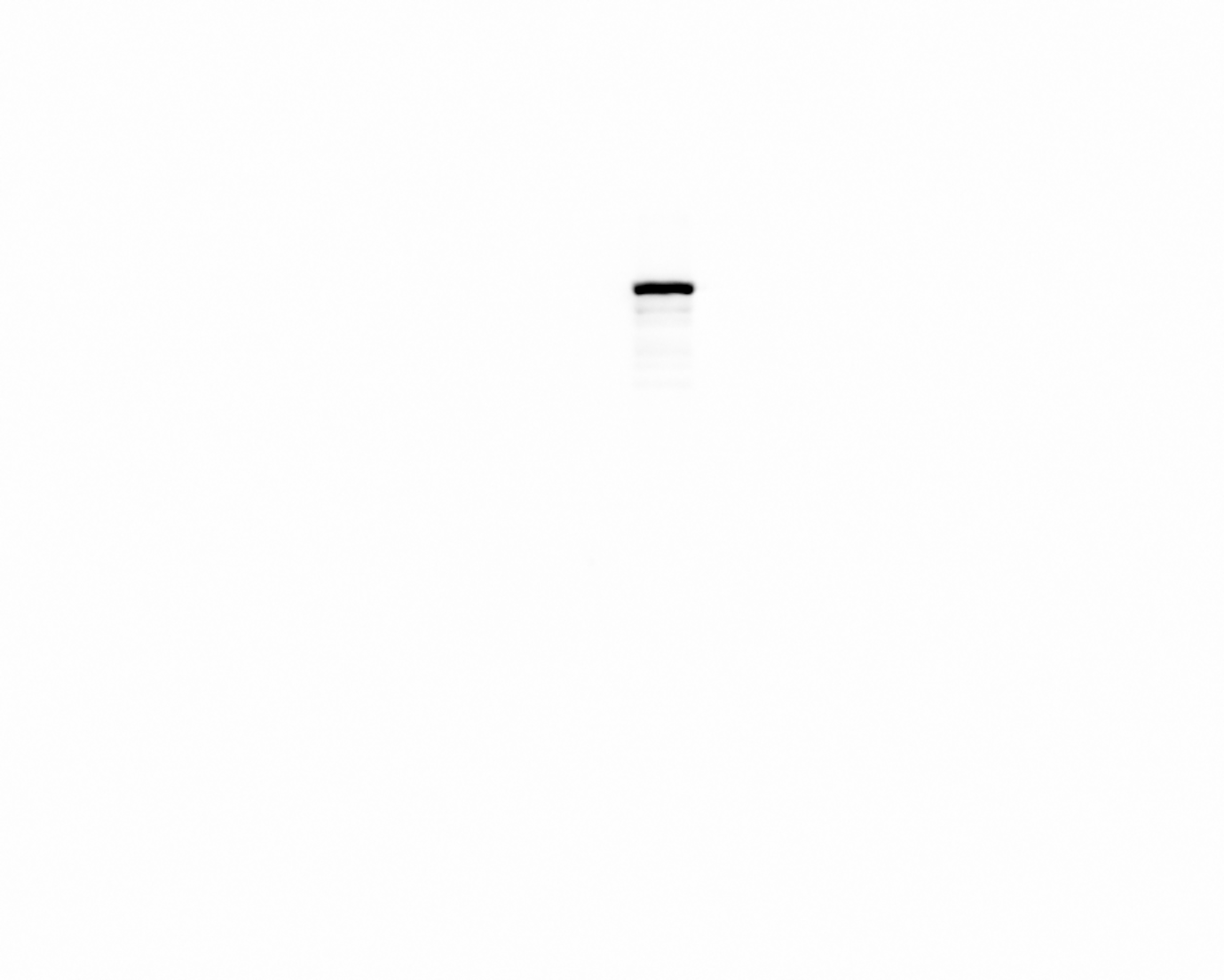

Supplement: Figure 4—source data 1. [file elife-96353-fig4-data1.zip › fig. 4A/input anti-flag_300ms-0006.tif]

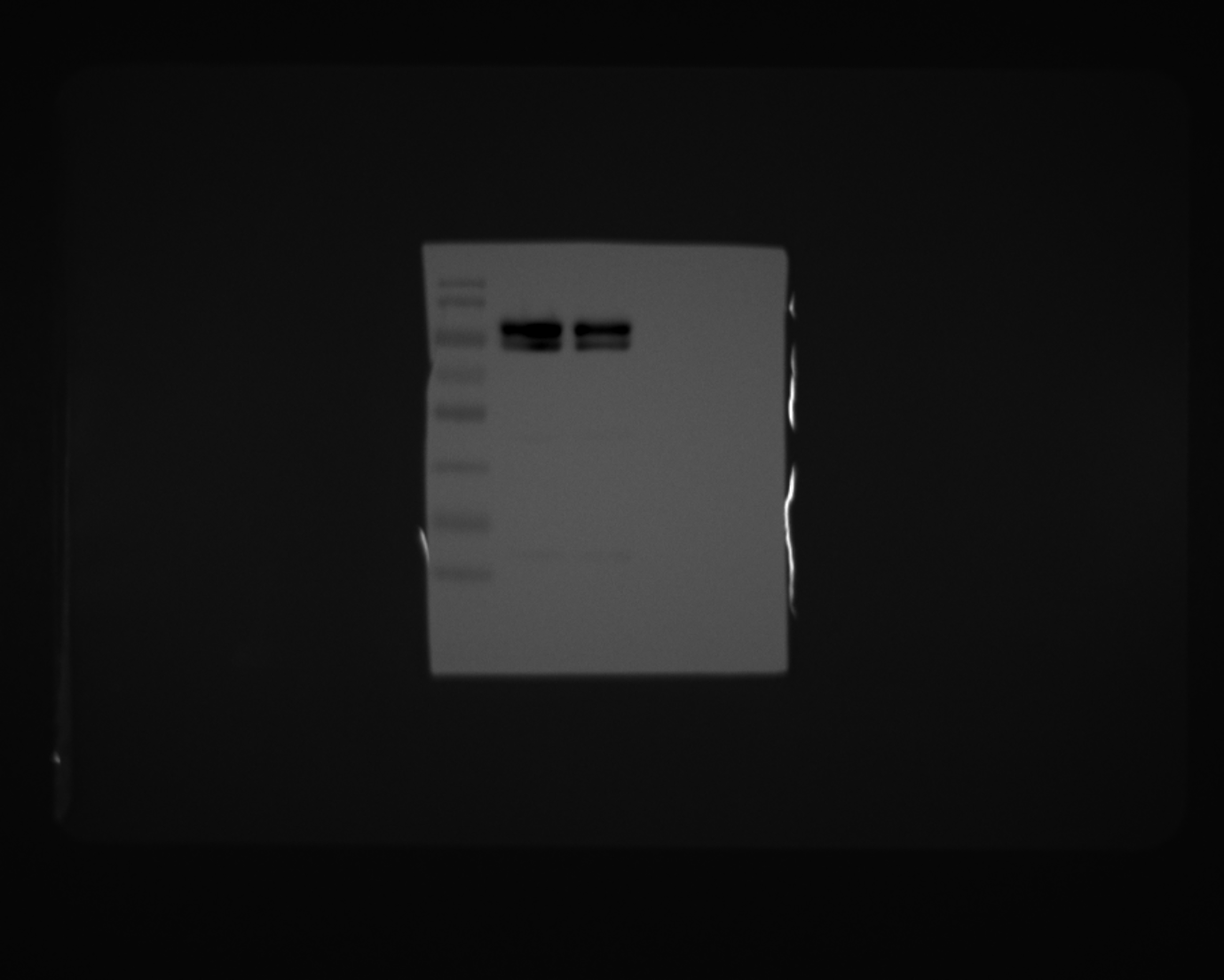

Supplement: Figure 4—source data 1. [file elife-96353-fig4-data1.zip › fig. 4A/input anti-gfp.tif]

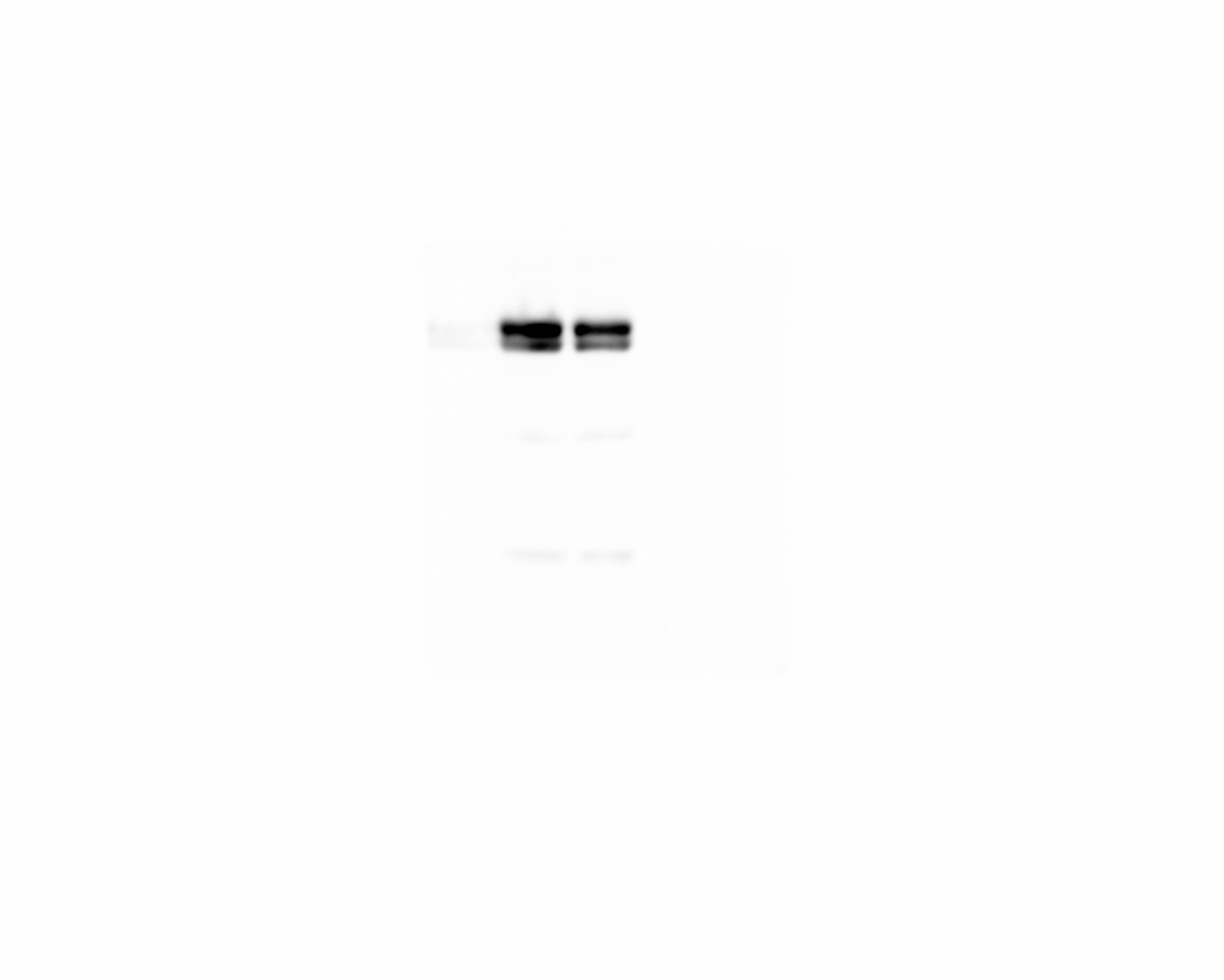

Supplement: Figure 4—source data 1. [file elife-96353-fig4-data1.zip › fig. 4A/input anti-gfp_1s-0004.tif]

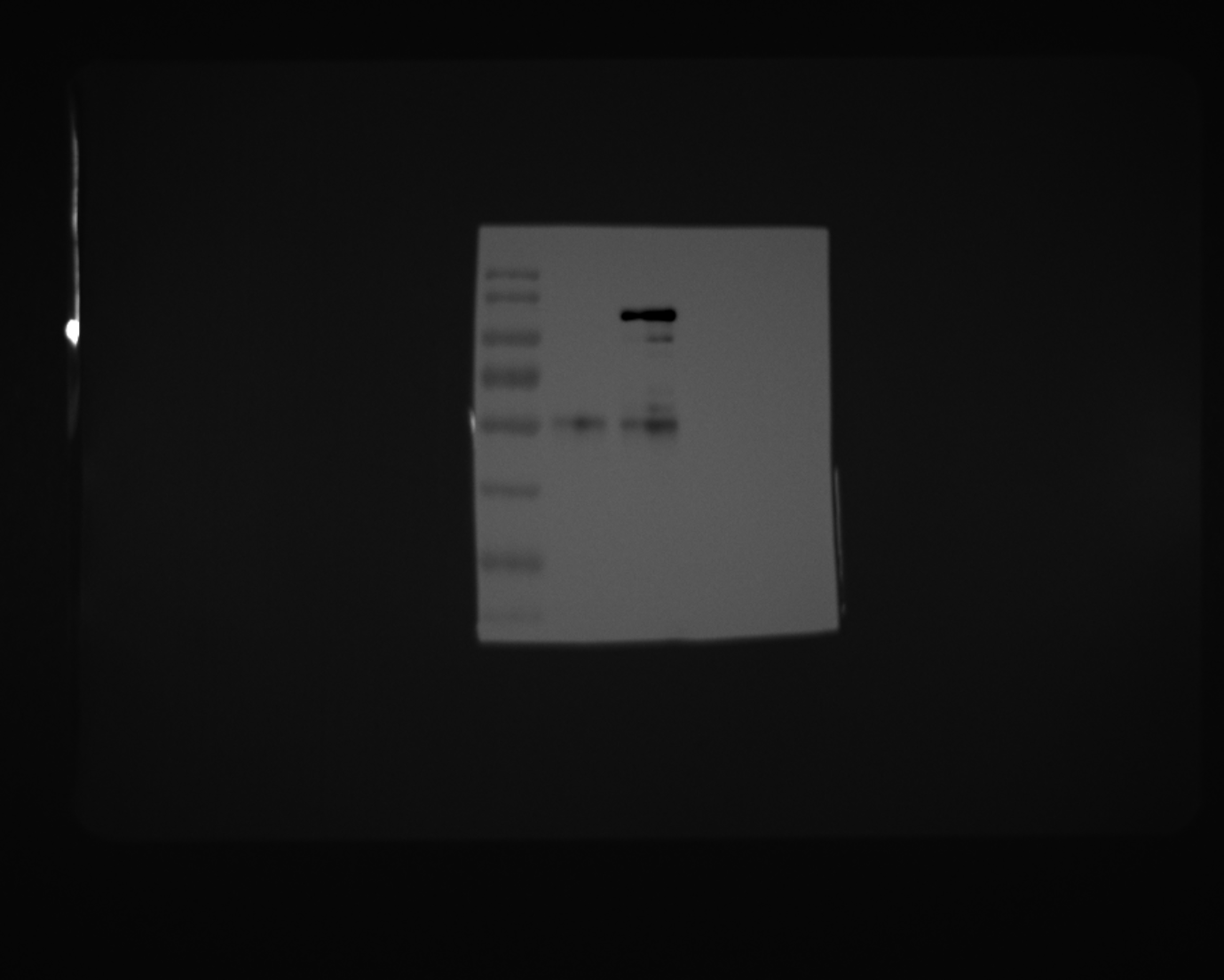

Supplement: Figure 4—source data 1. [file elife-96353-fig4-data1.zip › fig. 4A/IP anti-flag.tif]

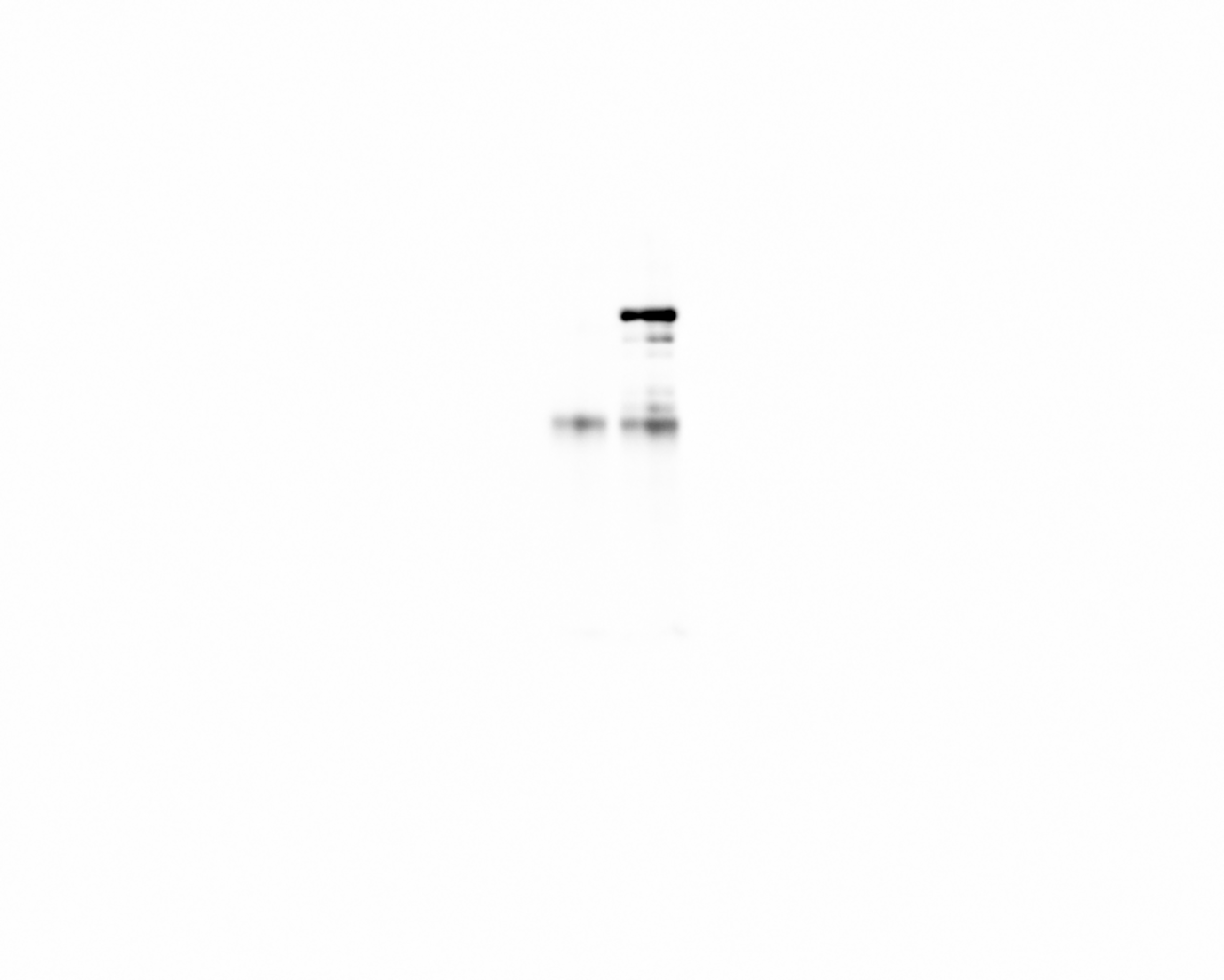

Supplement: Figure 4—source data 1. [file elife-96353-fig4-data1.zip › fig. 4A/IP anti-flag_100ms-0005.tif]

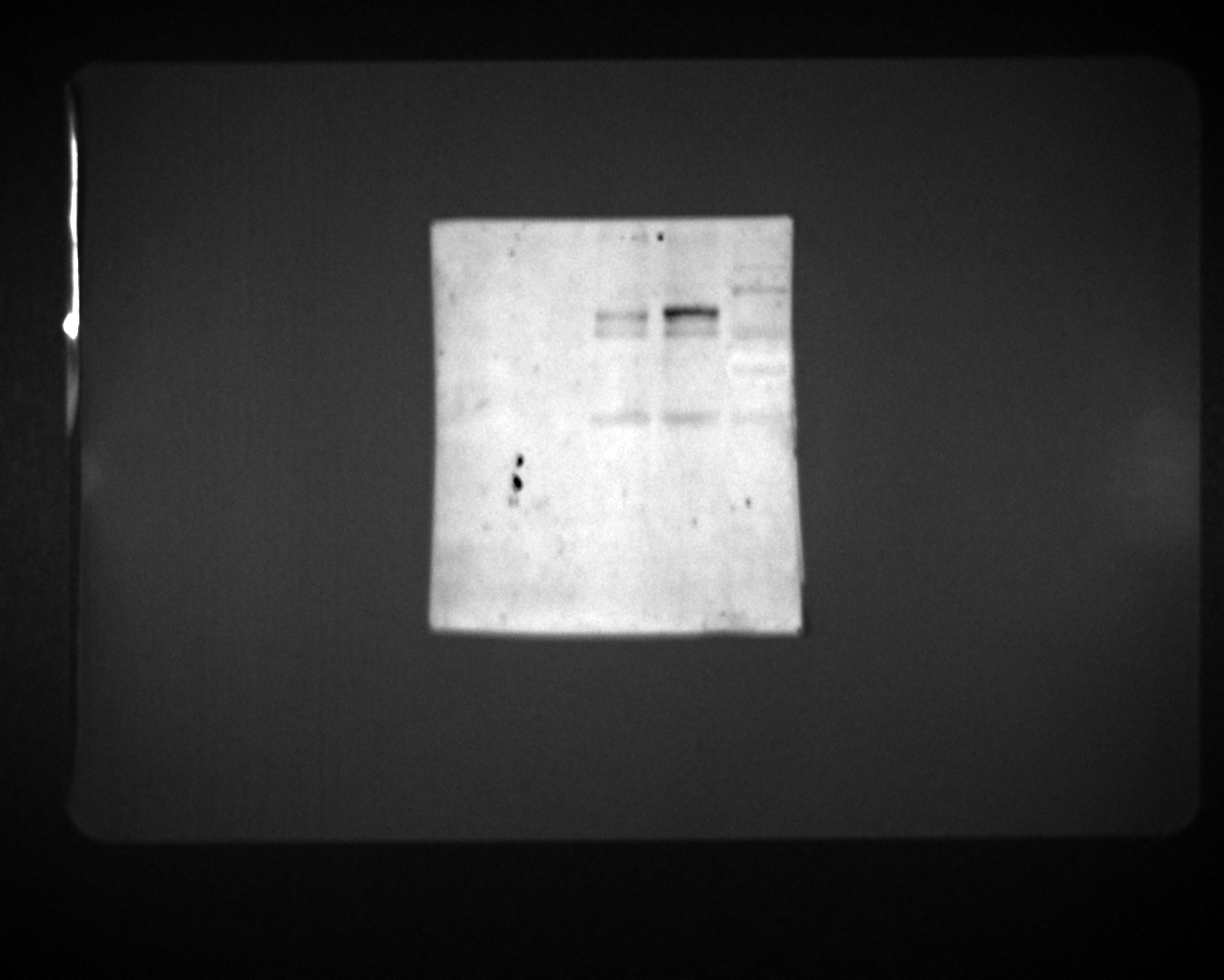

Supplement: Figure 4—source data 1. [file elife-96353-fig4-data1.zip › fig. 4A/IP anti-gfp.tif]

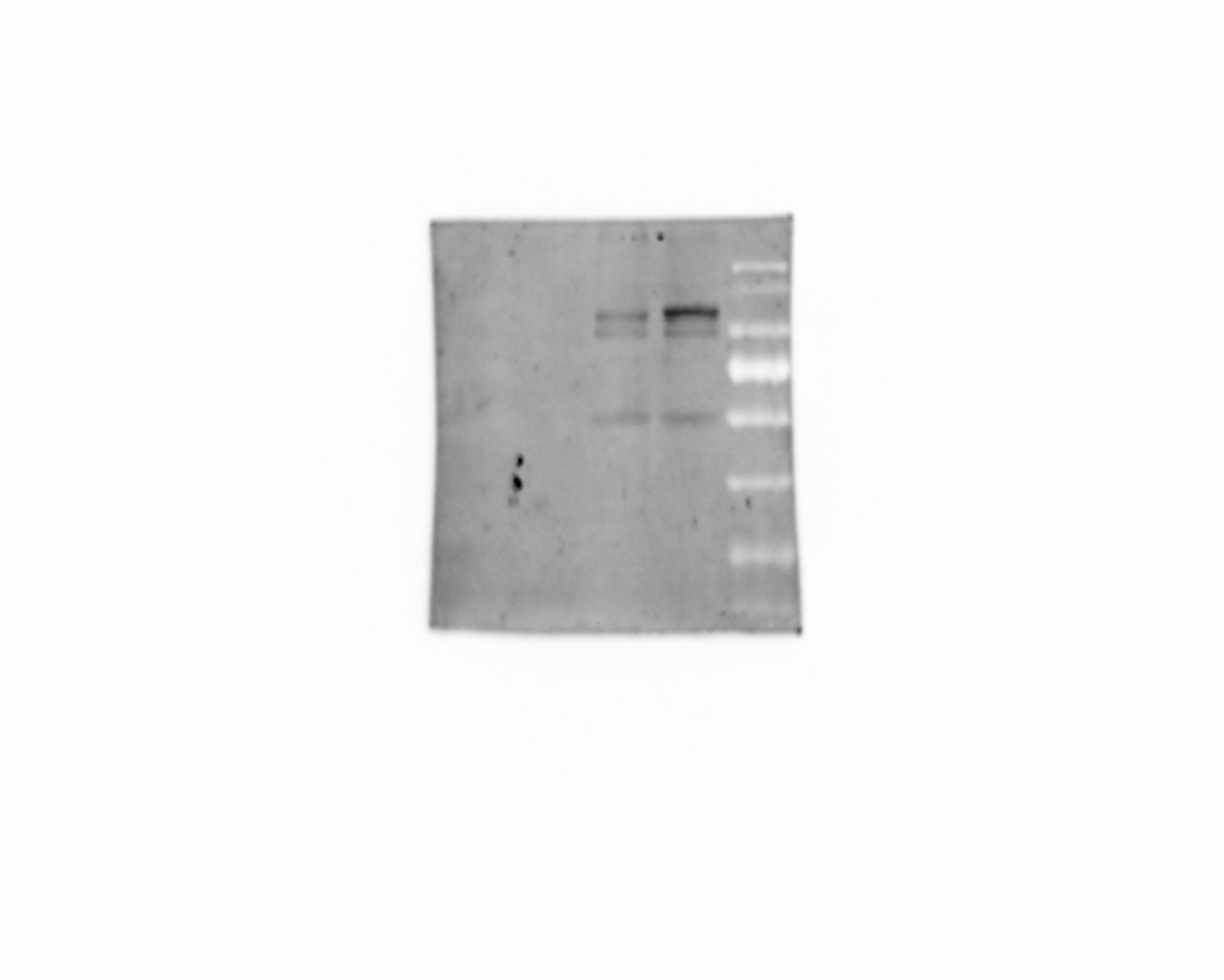

Supplement: Figure 4—source data 1. [file elife-96353-fig4-data1.zip › fig. 4A/IP anti-gfp_10s-0006.tif]

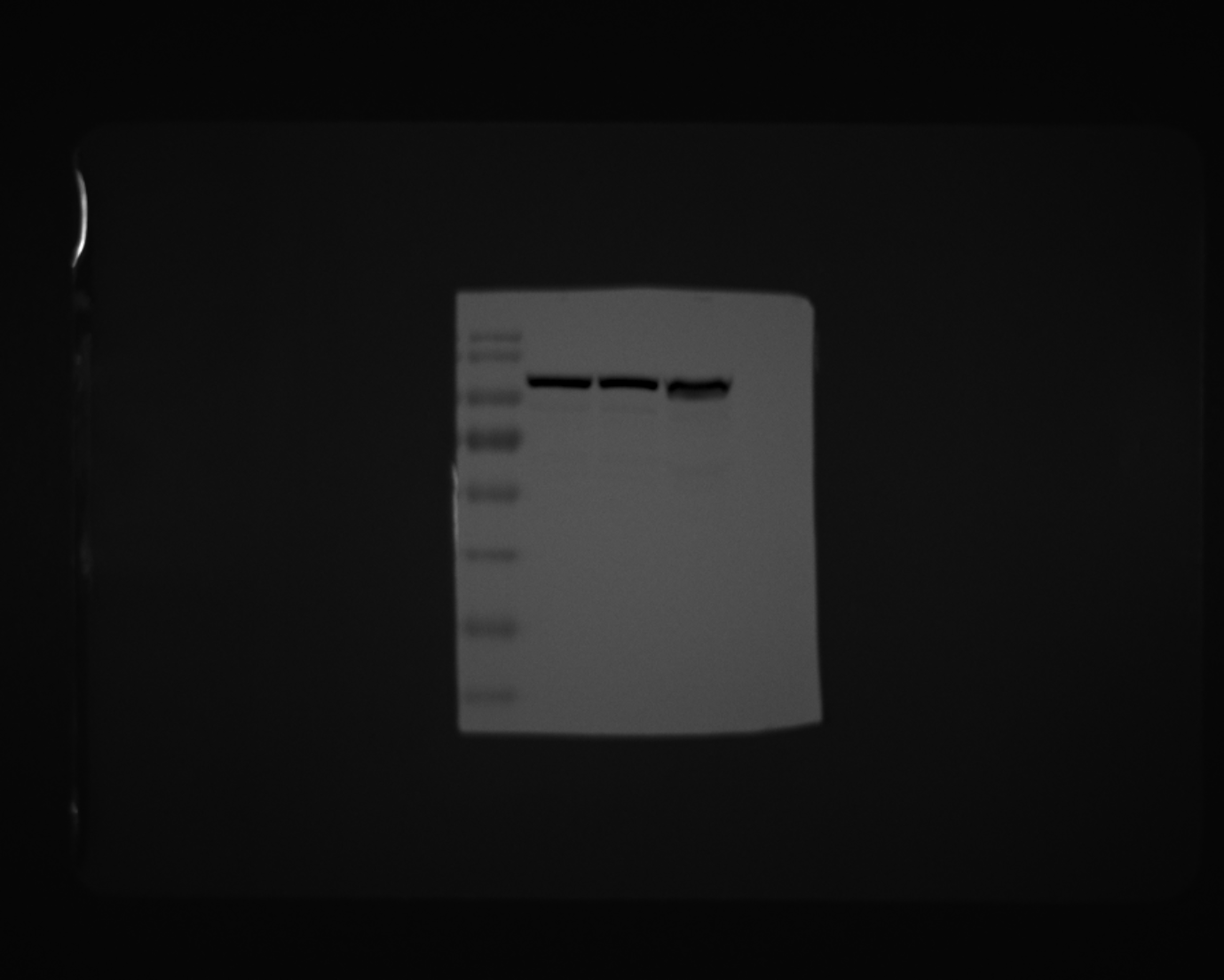

Supplement: Figure 4—source data 3. [file elife-96353-fig4-data3.zip › fig. 4B/input anti-flag.tif]

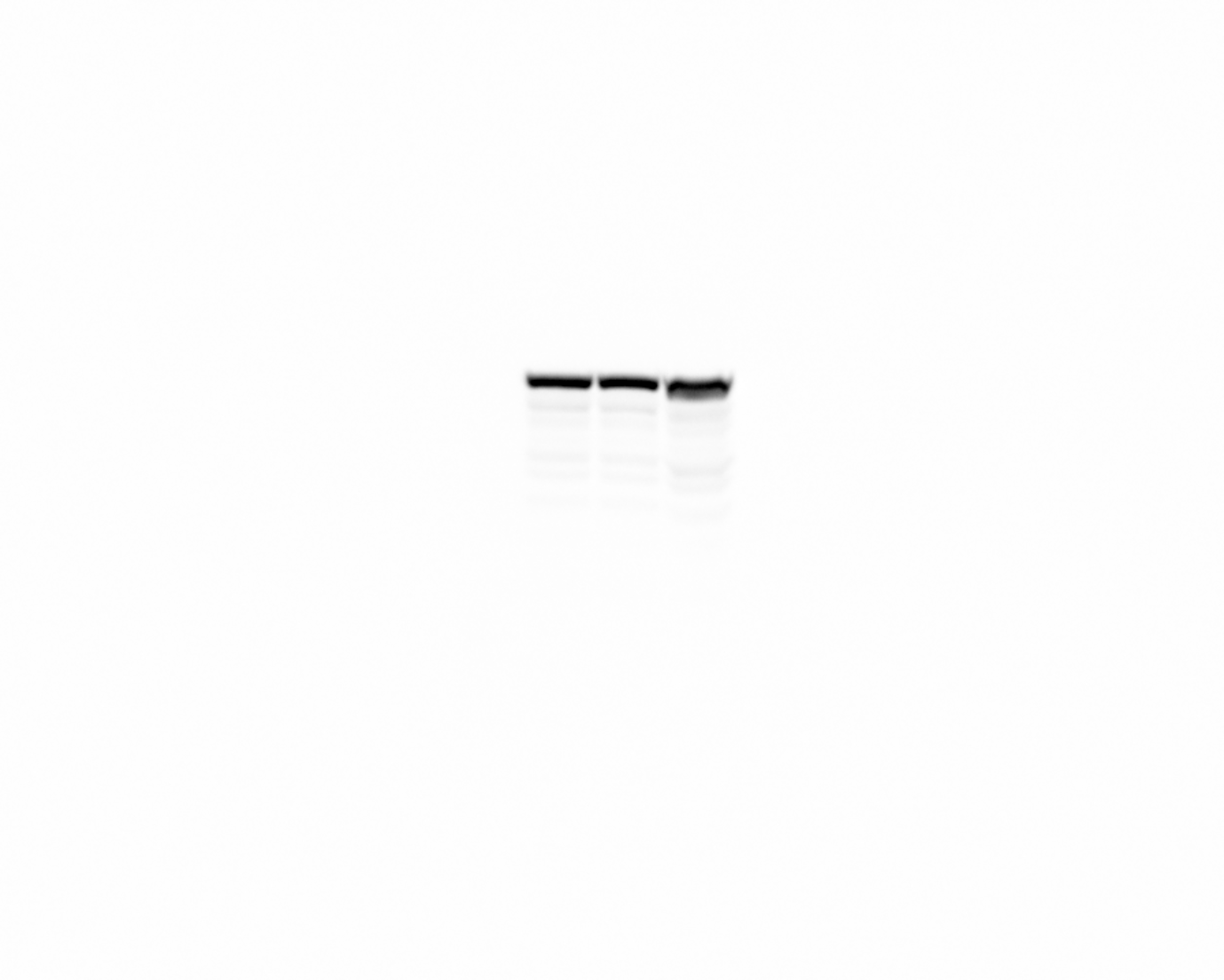

Supplement: Figure 4—source data 3. [file elife-96353-fig4-data3.zip › fig. 4B/input anti-flag_200ms-0005.tif]

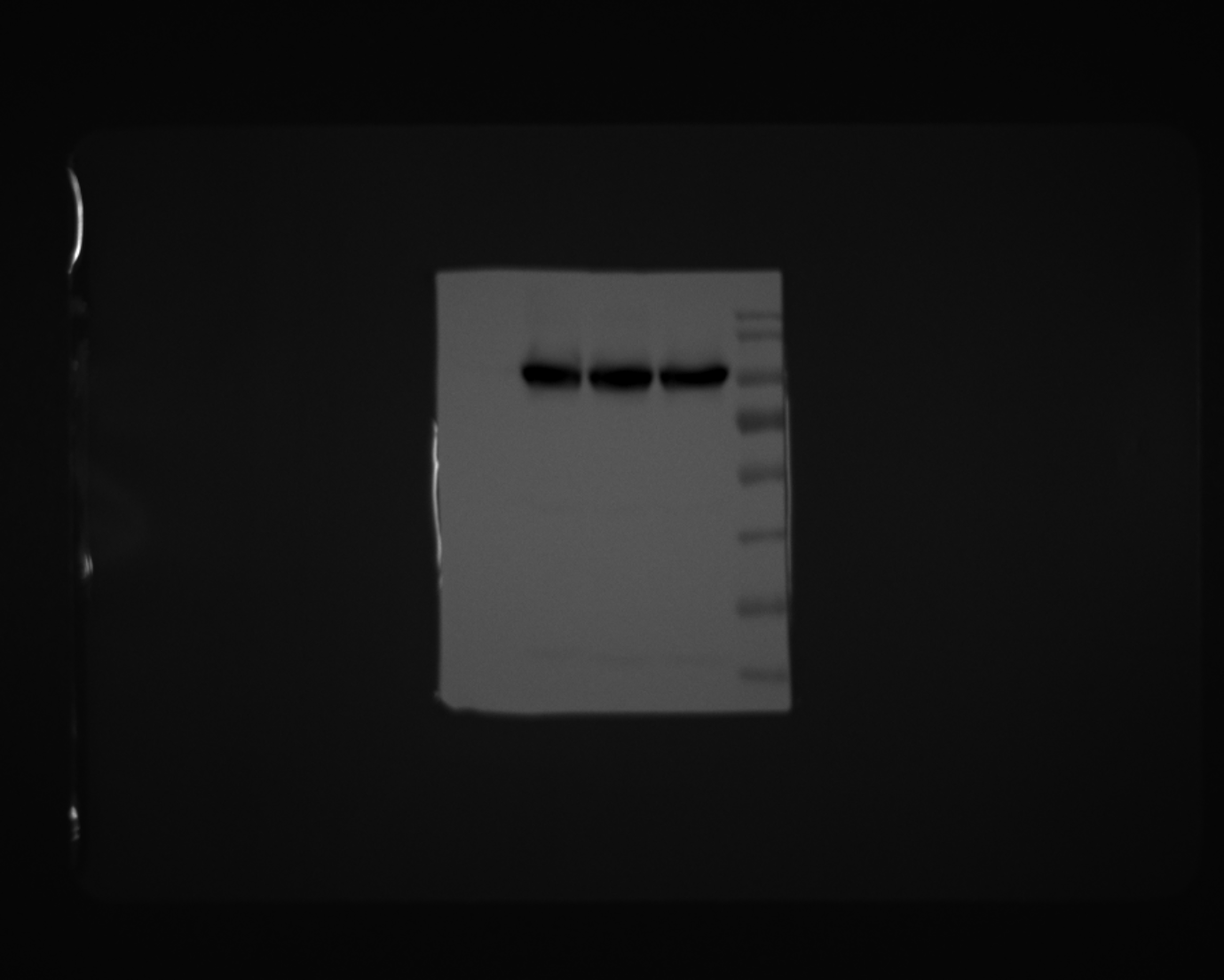

Supplement: Figure 4—source data 3. [file elife-96353-fig4-data3.zip › fig. 4B/input anti-gfp.tif]

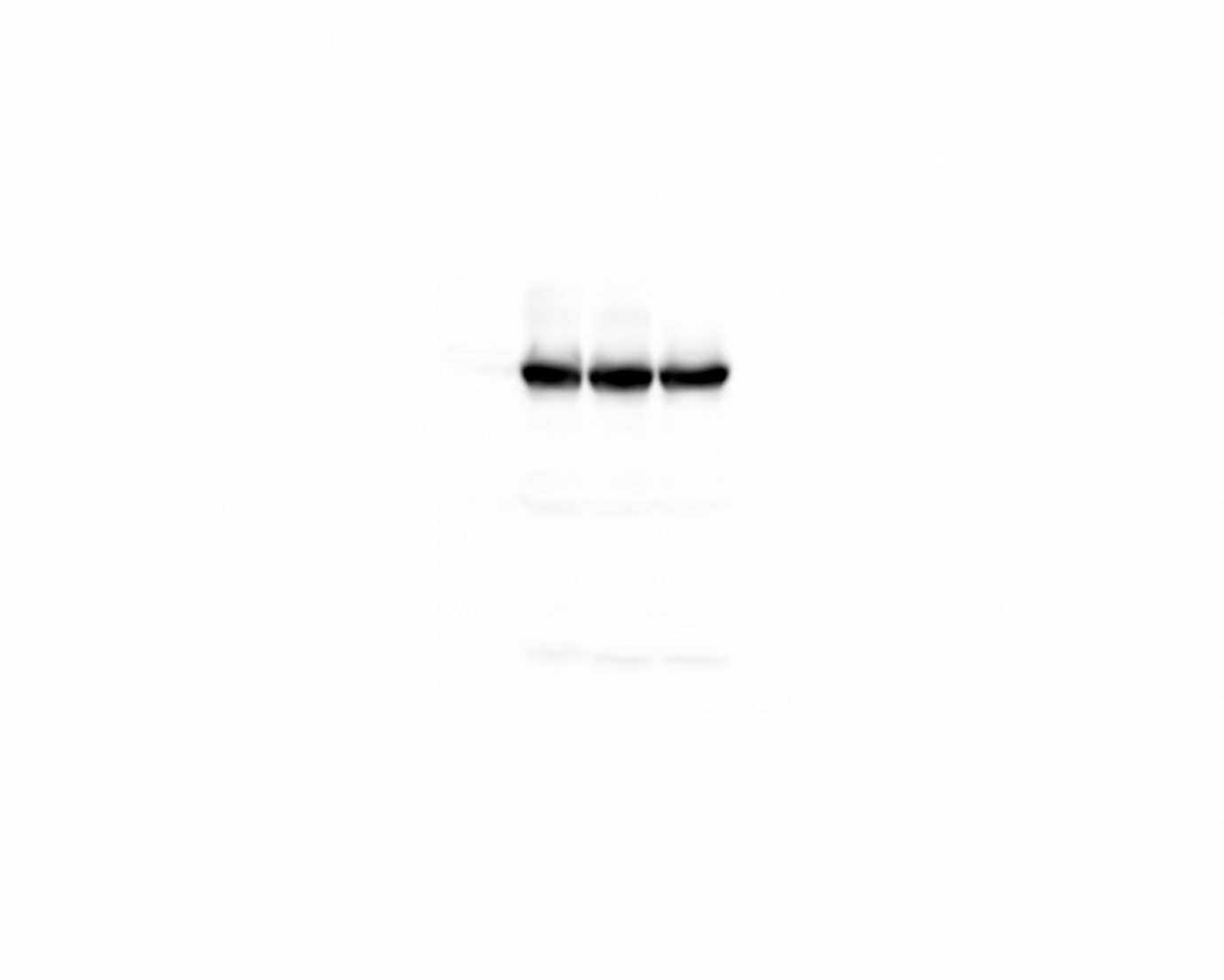

Supplement: Figure 4—source data 3. [file elife-96353-fig4-data3.zip › fig. 4B/input anti-gfp_400ms-0003.tif]

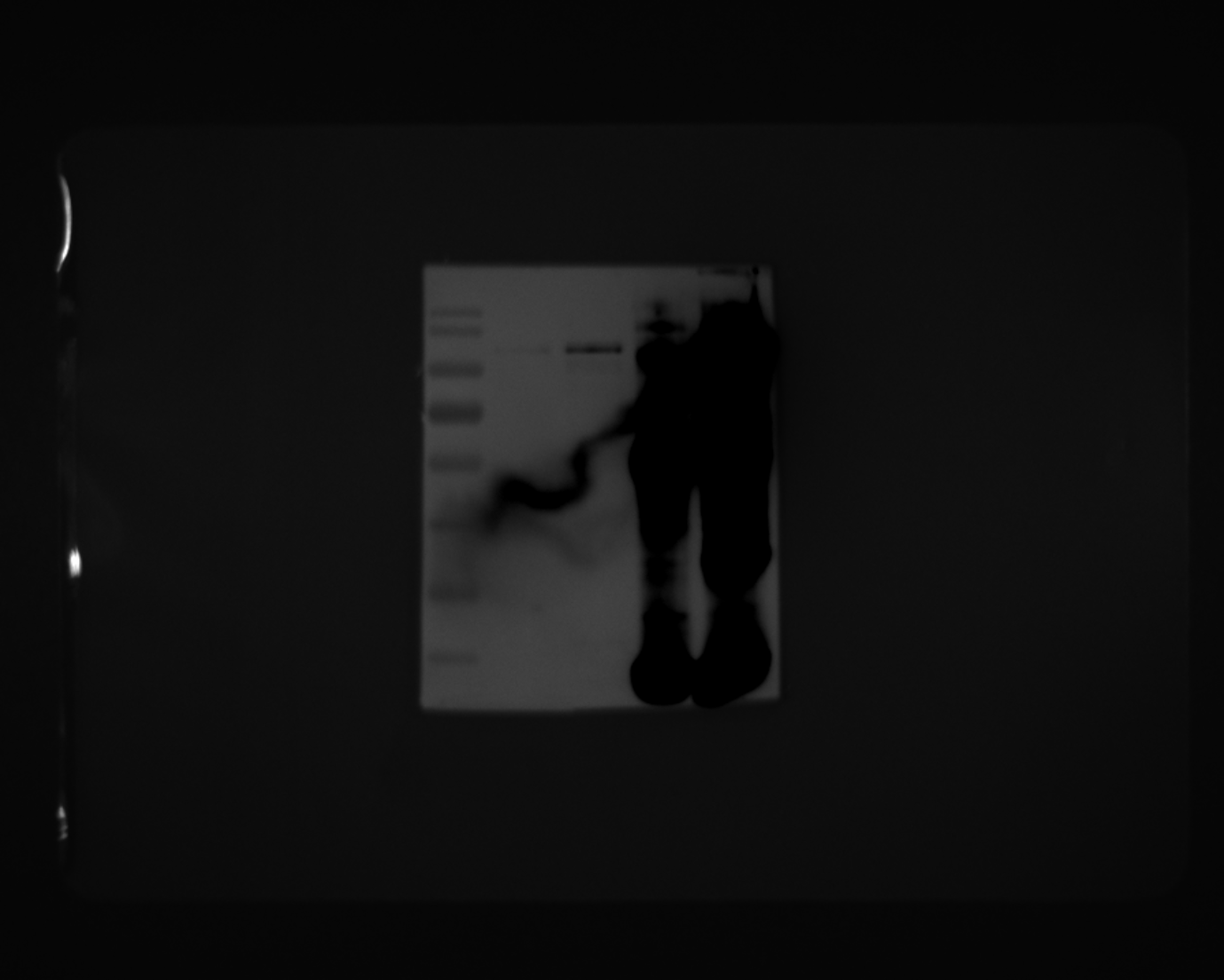

Supplement: Figure 4—source data 3. [file elife-96353-fig4-data3.zip › fig. 4B/IP anti-flag.tif]

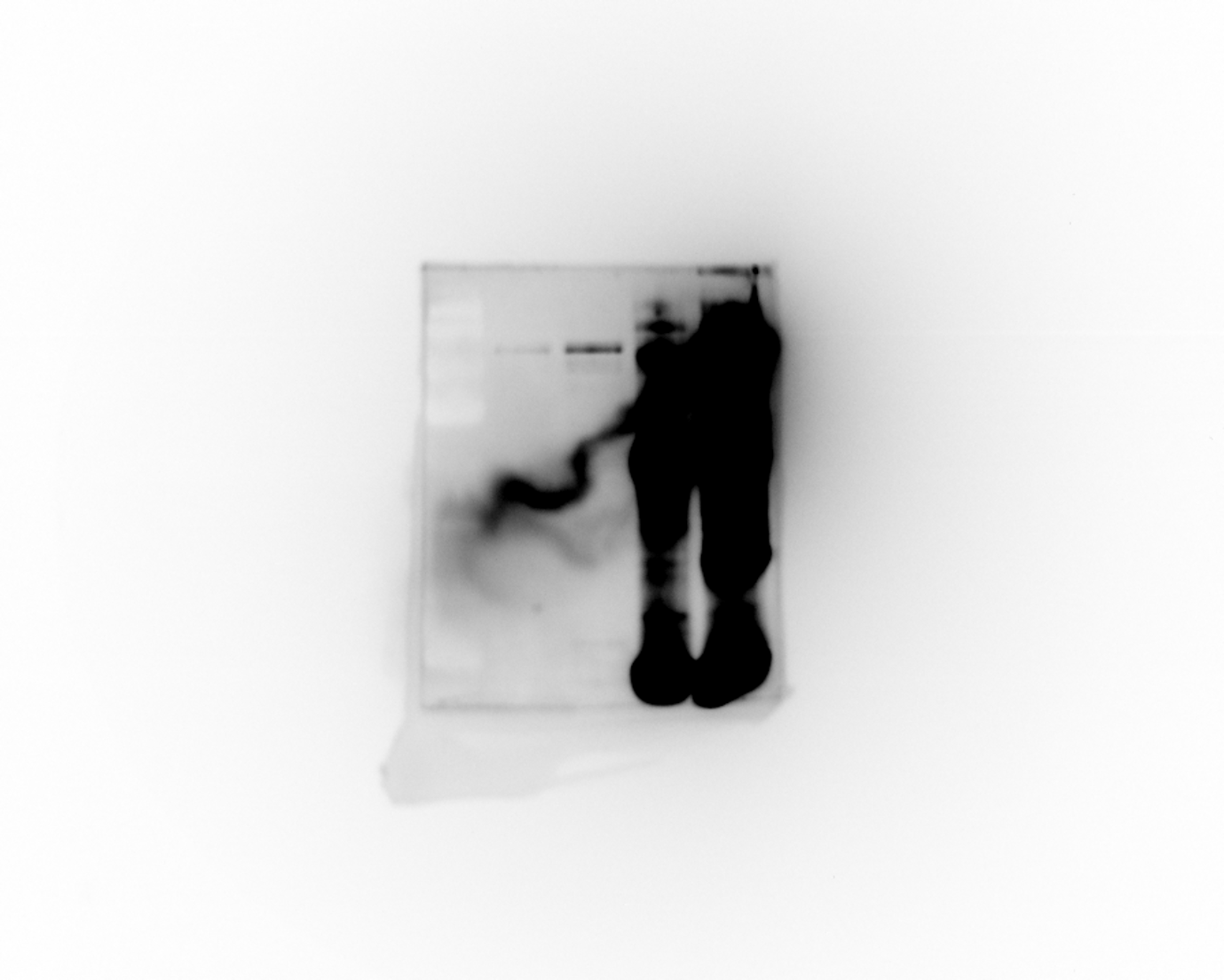

Supplement: Figure 4—source data 3. [file elife-96353-fig4-data3.zip › fig. 4B/IP anti-flag_30s-0008.tif]

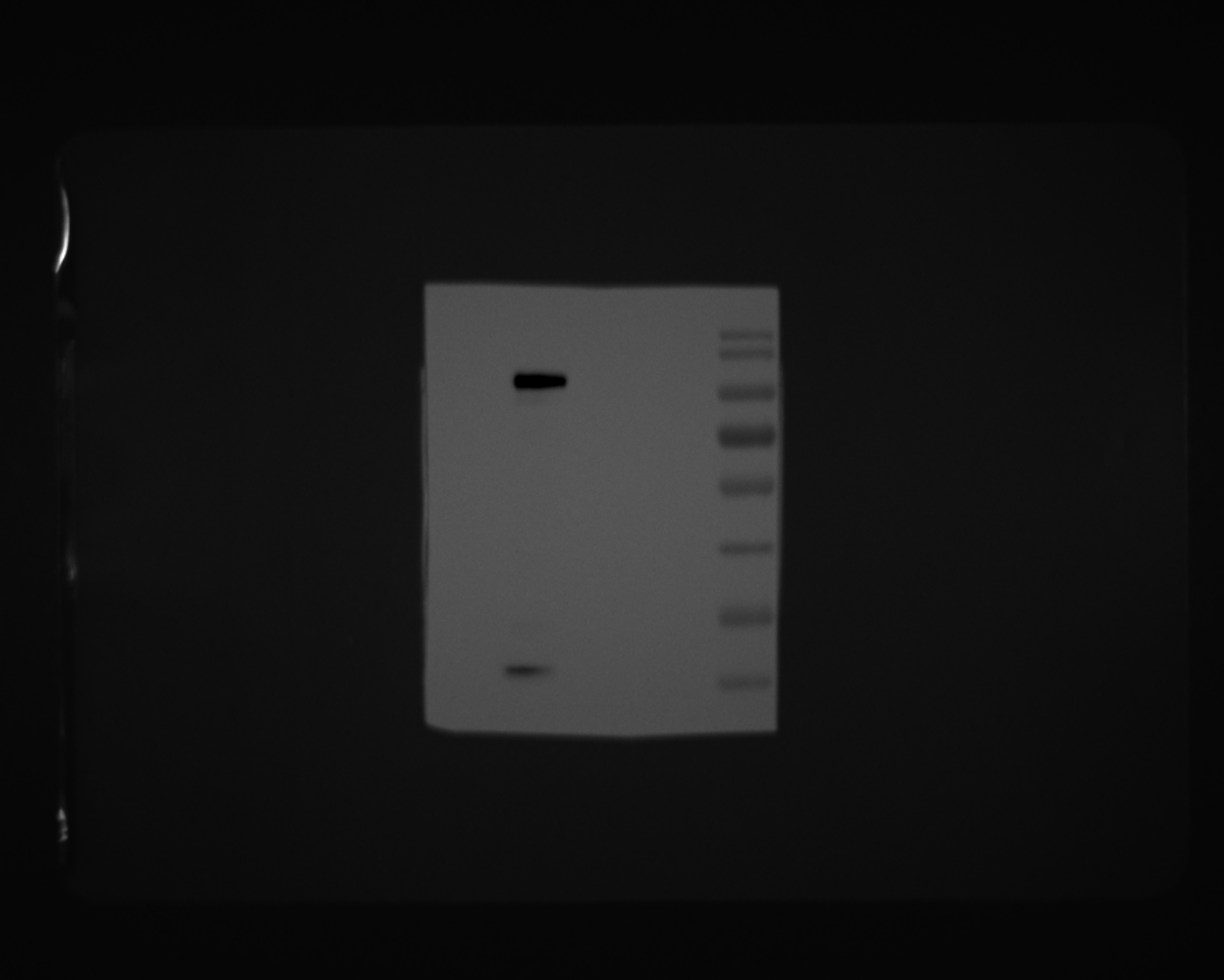

Supplement: Figure 4—source data 3. [file elife-96353-fig4-data3.zip › fig. 4B/IP anti-gfp.tif]

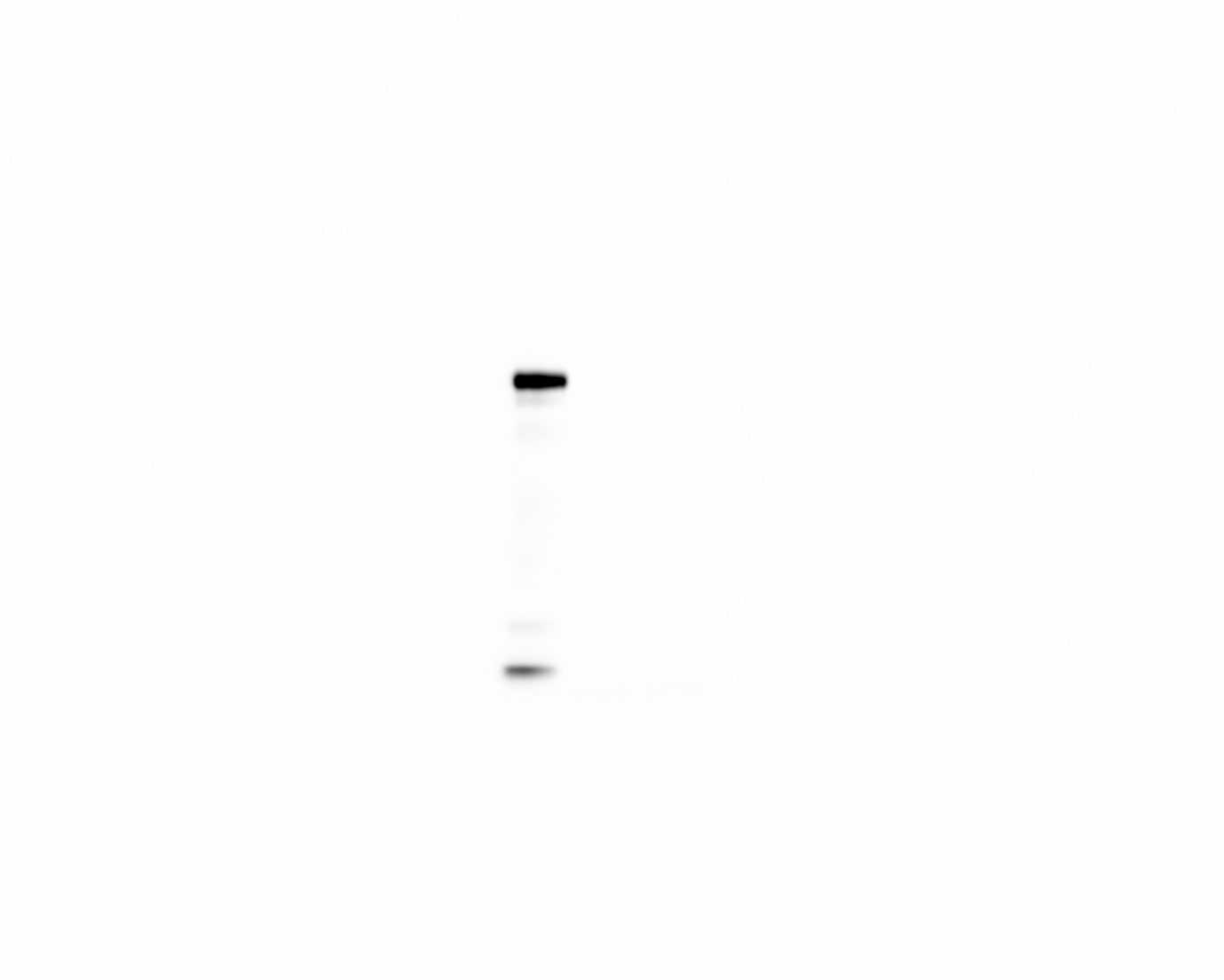

Supplement: Figure 4—source data 3. [file elife-96353-fig4-data3.zip › fig. 4B/IP anti-gfp_100ms-0004.tif]

**Figure 4B**

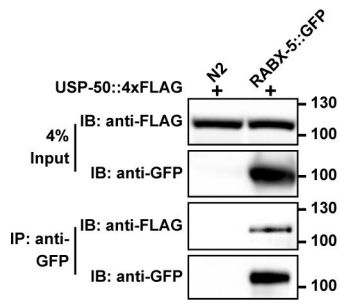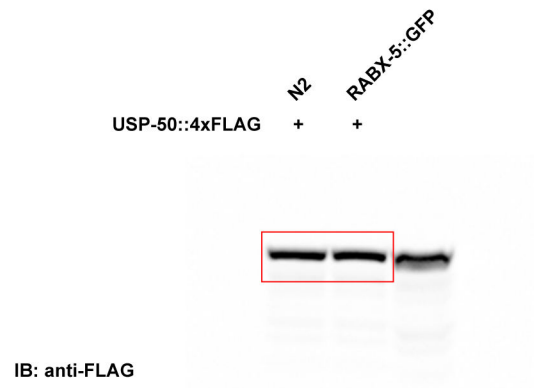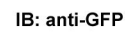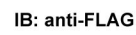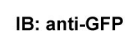

Supplement: Figure 4—source data 4. [file elife-96353-fig4-data4.pdf]

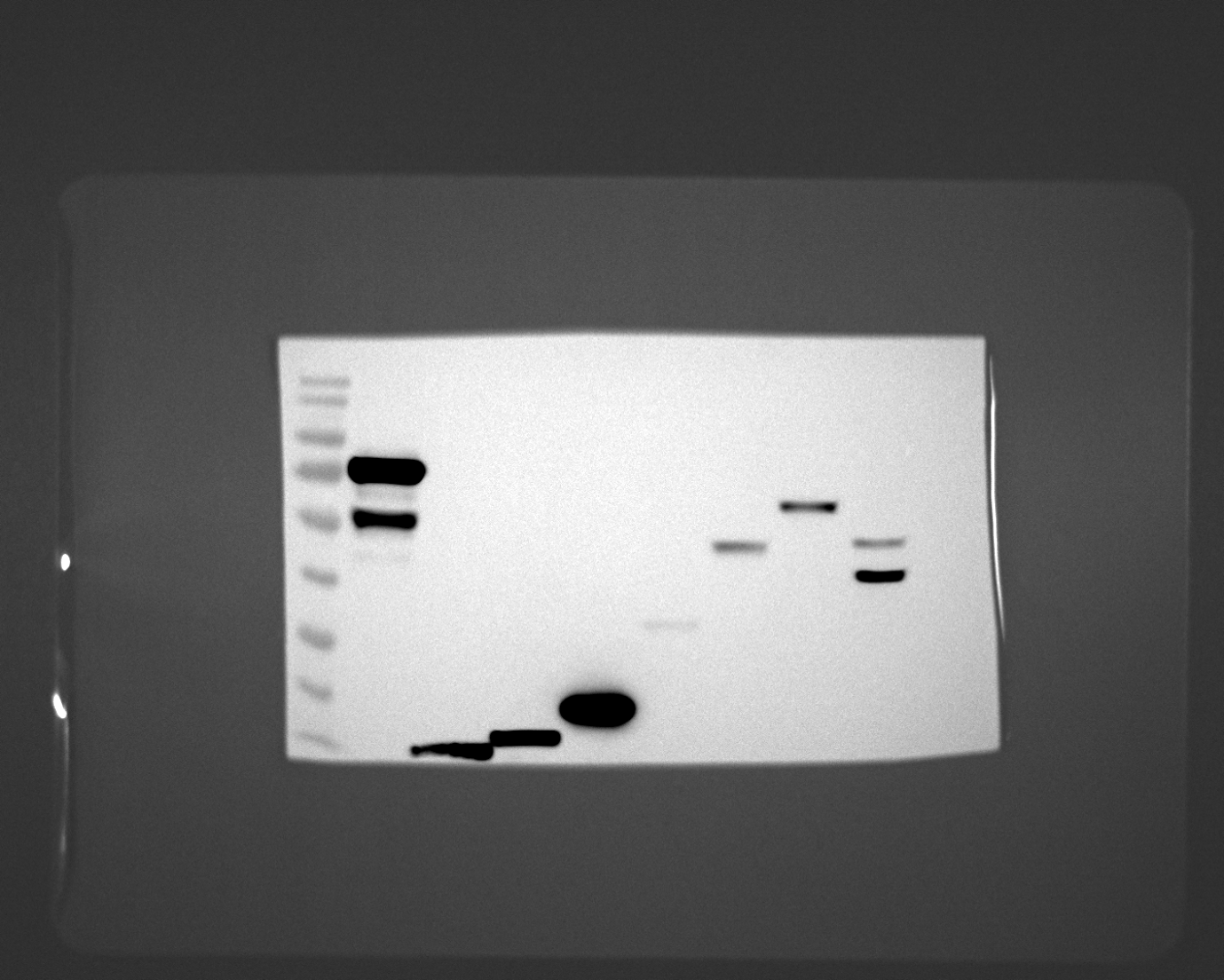

Supplement: Figure 4—source data 5. [file elife-96353-fig4-data5.zip › fig. 4D/input anti-flag left.tif]

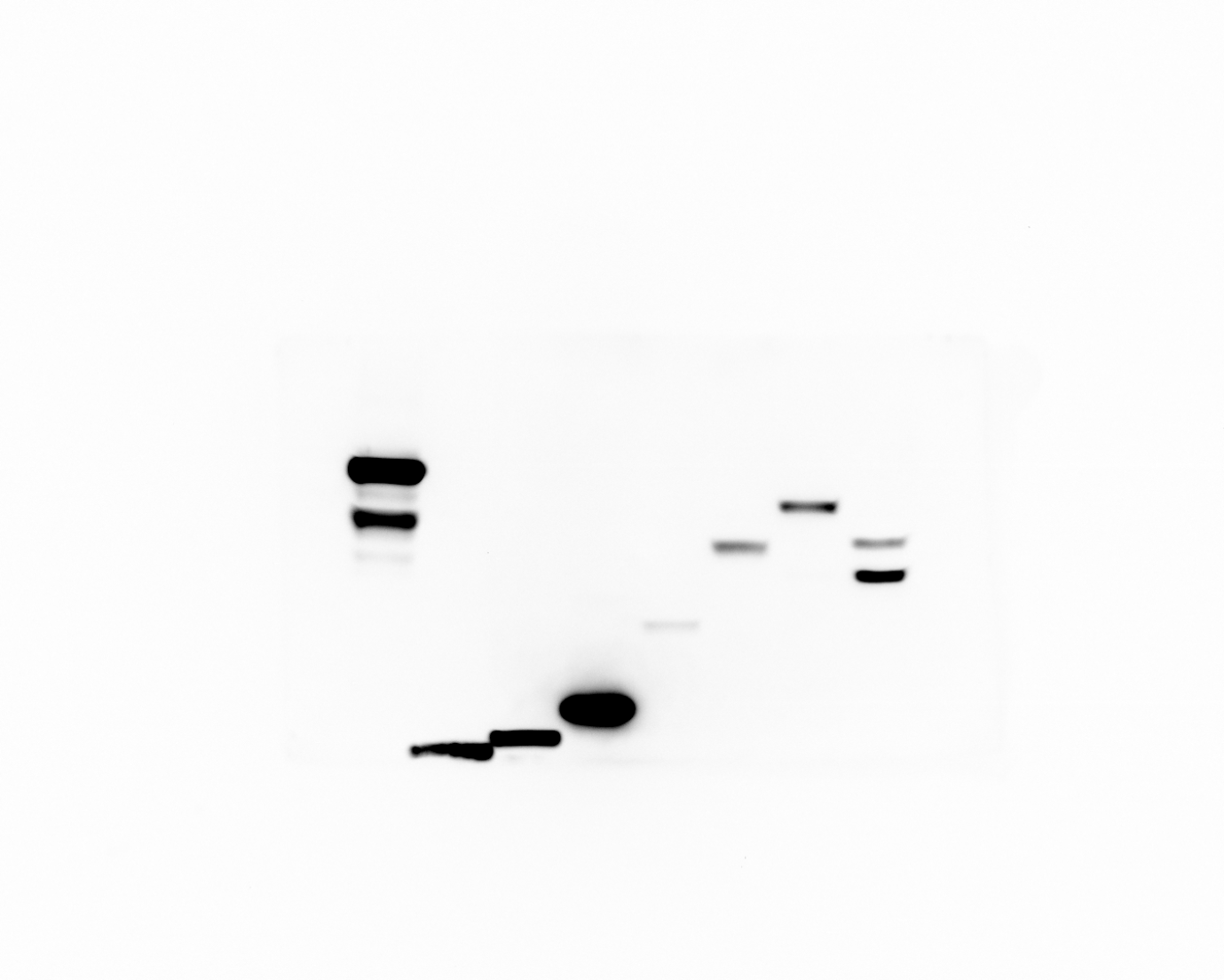

Supplement: Figure 4—source data 5. [file elife-96353-fig4-data5.zip › fig. 4D/input anti-flag left_1min_opt-0003.tif]

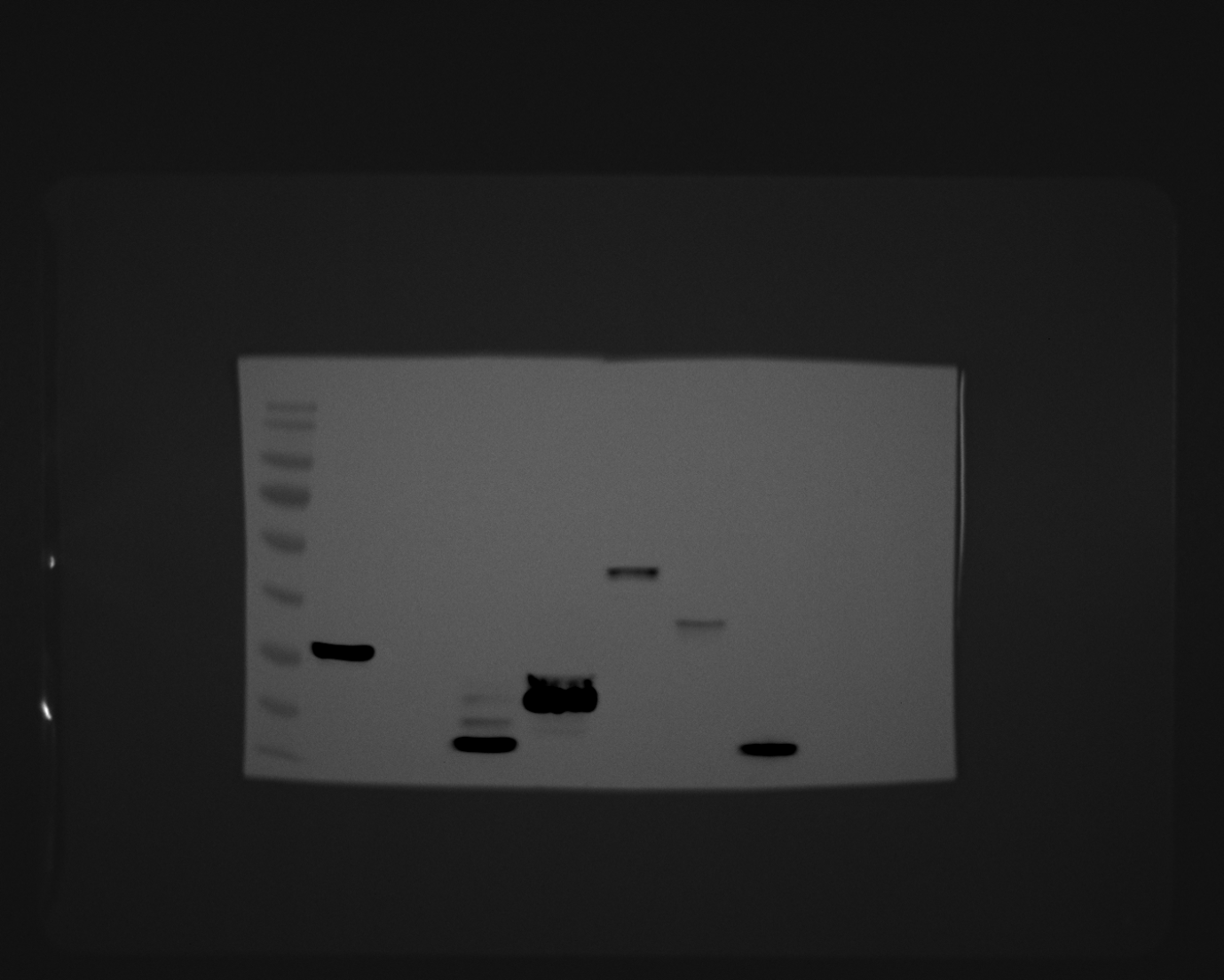

Supplement: Figure 4—source data 5. [file elife-96353-fig4-data5.zip › fig. 4D/input anti-flag right.tif]

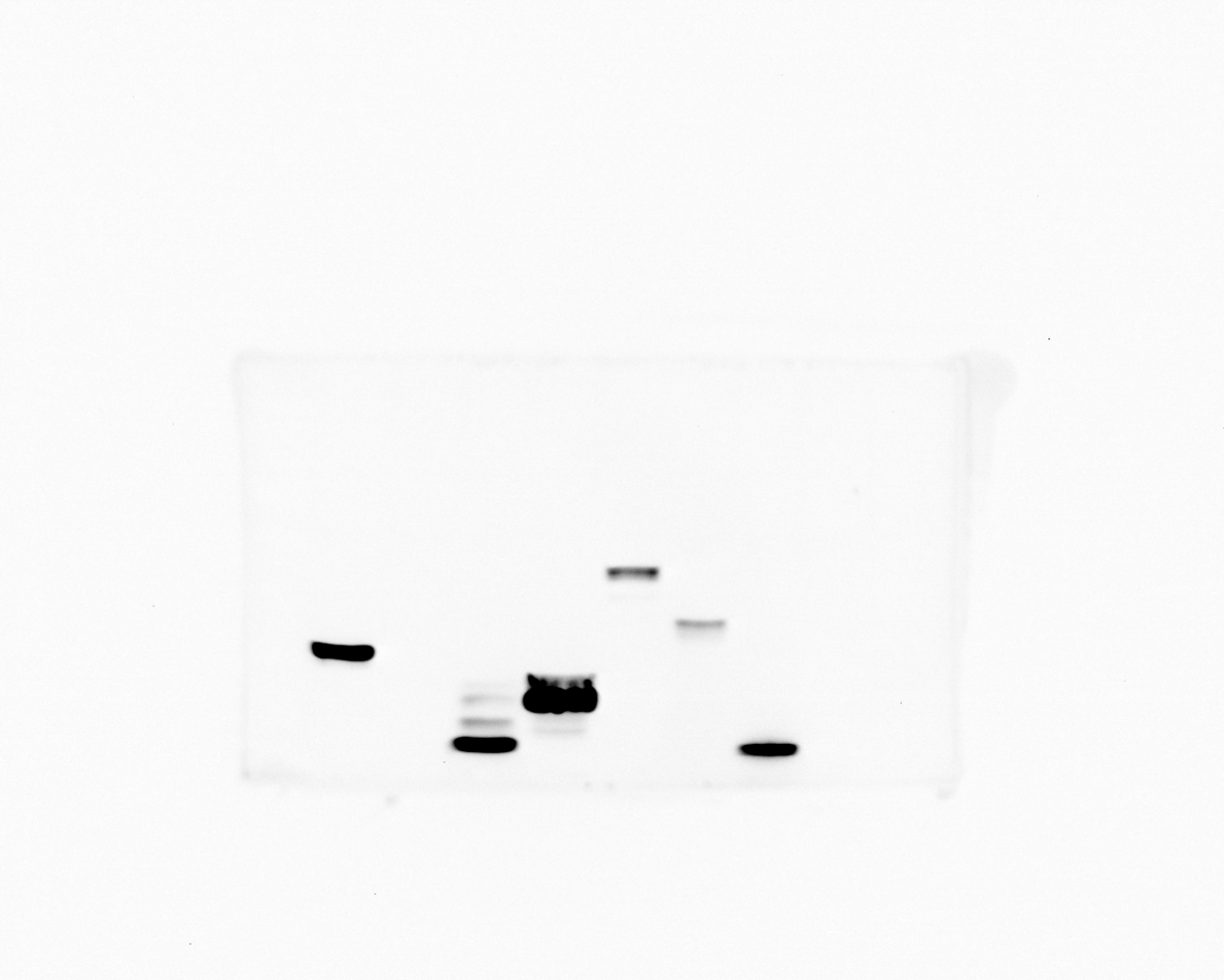

Supplement: Figure 4—source data 5. [file elife-96353-fig4-data5.zip › fig. 4D/input anti-flag right_1min_opt-0009.tif]

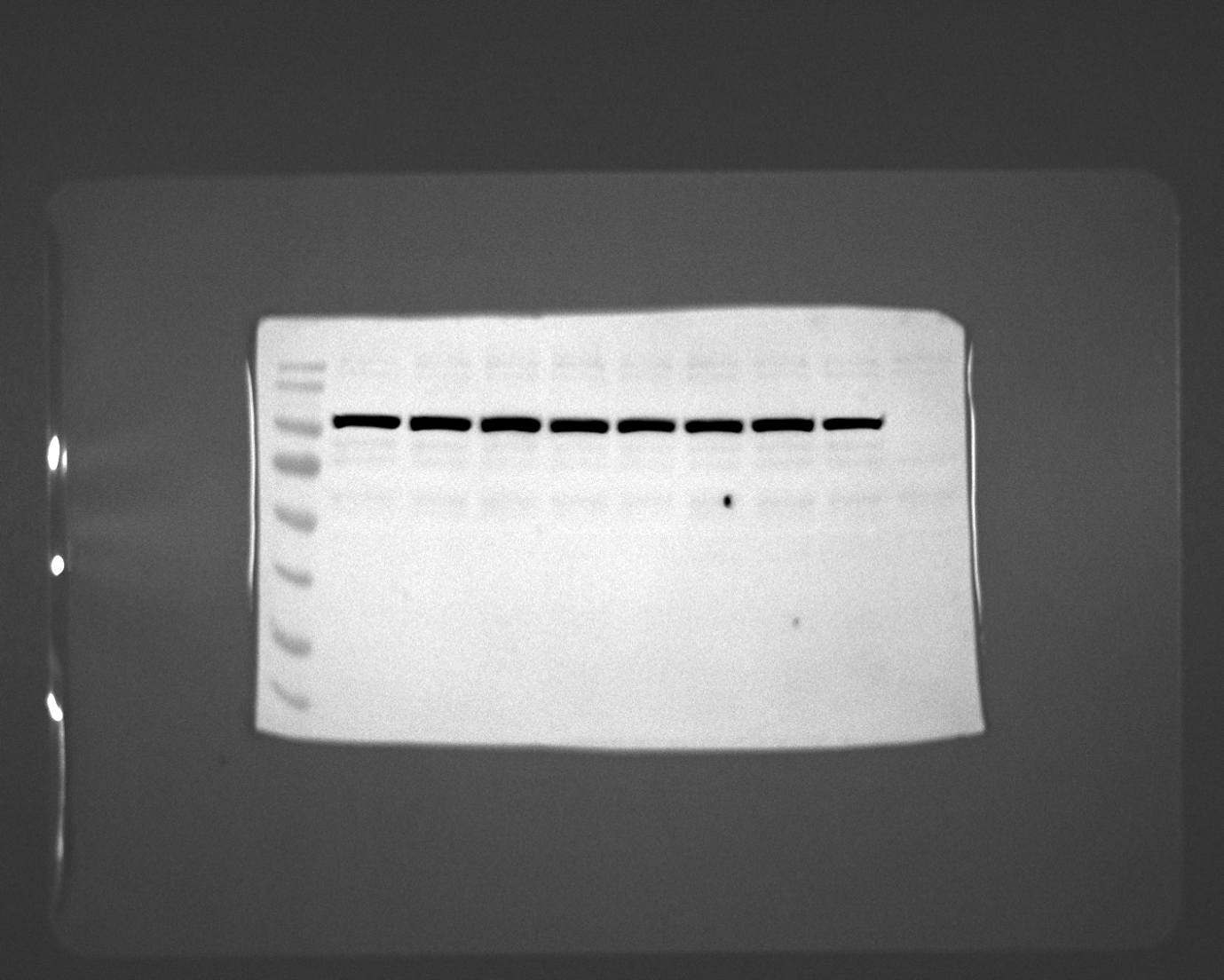

Supplement: Figure 4—source data 5. [file elife-96353-fig4-data5.zip › fig. 4D/input anti-myc left.tif]

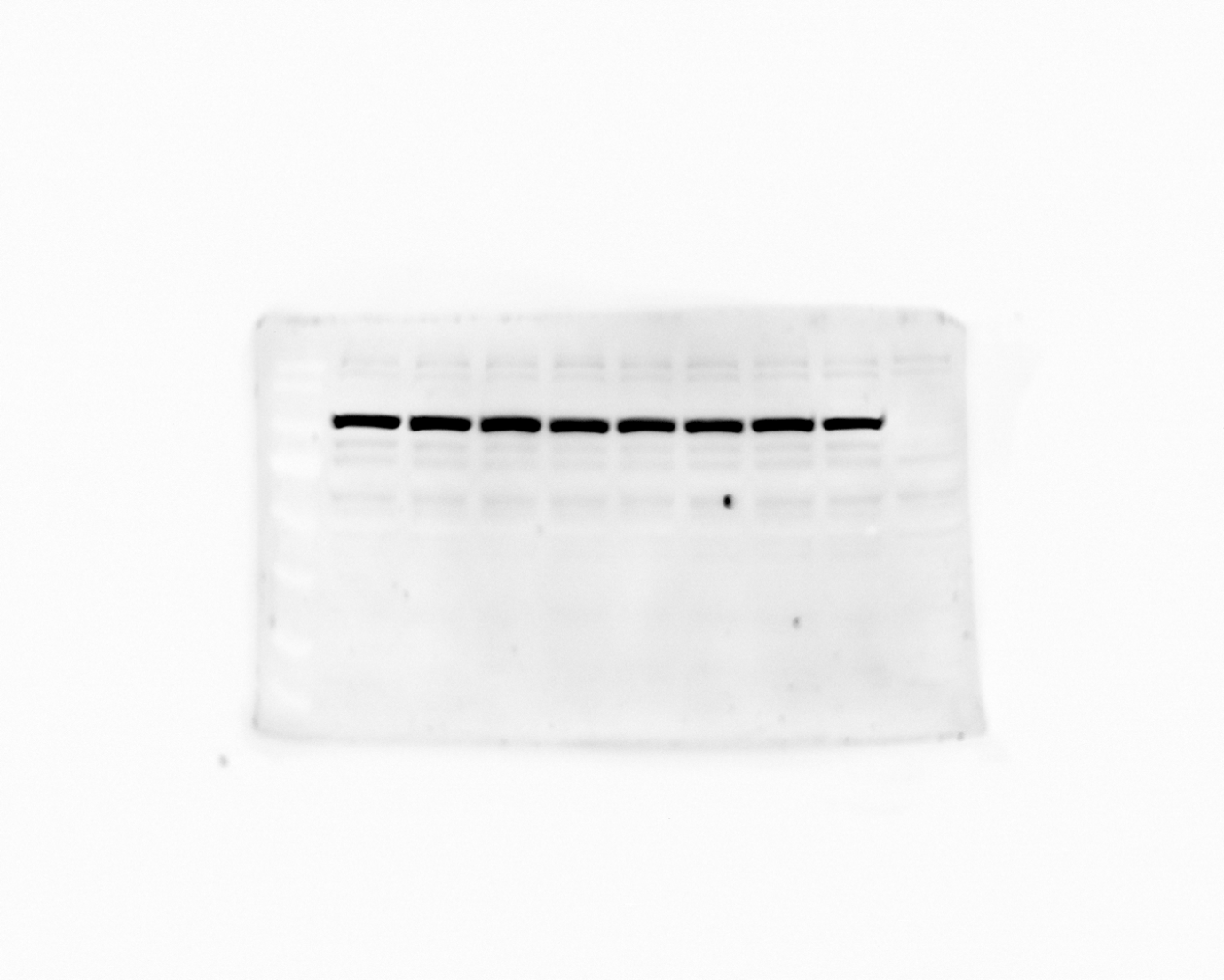

Supplement: Figure 4—source data 5. [file elife-96353-fig4-data5.zip › fig. 4D/input anti-myc left_20s_opt-0007.tif]

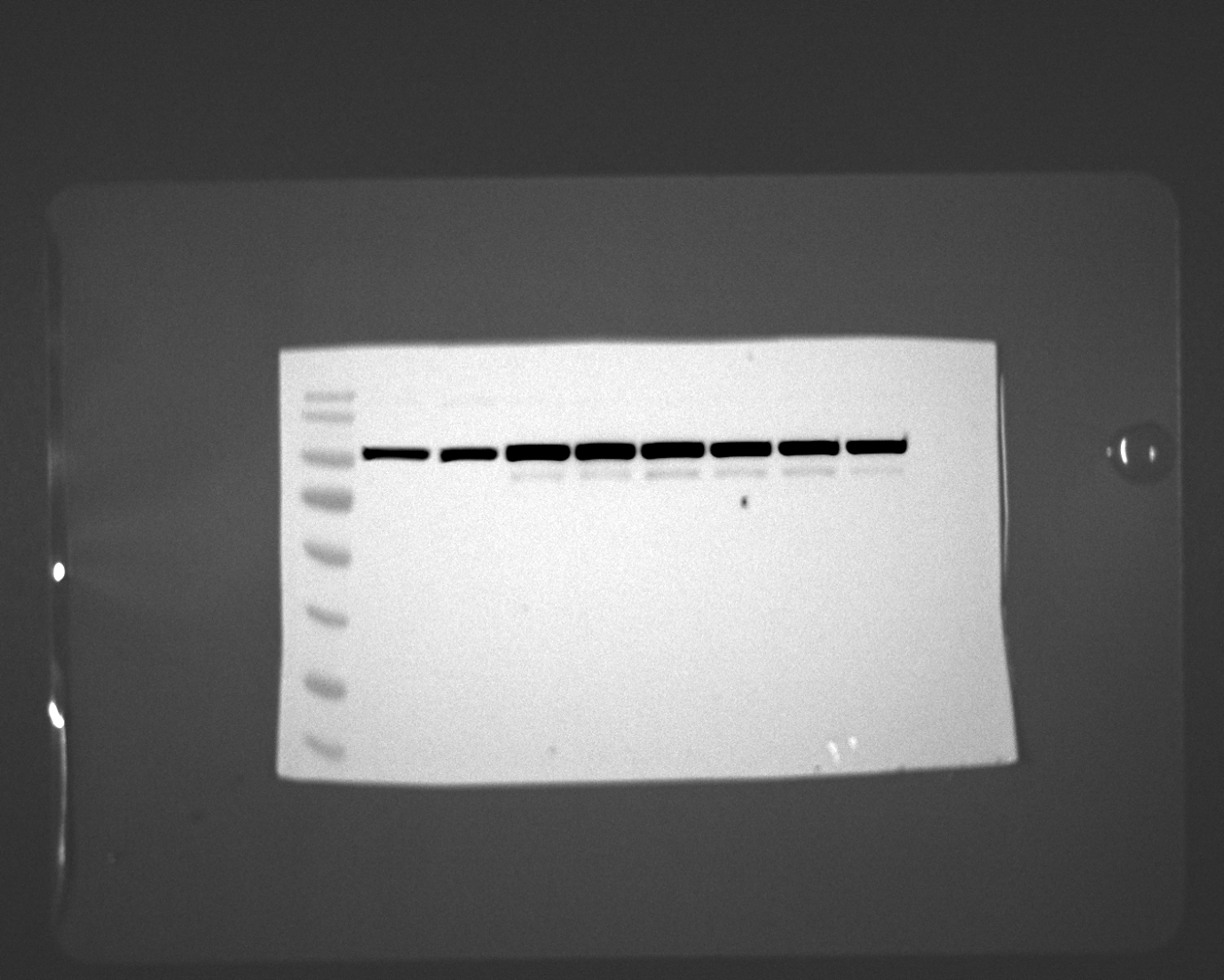

Supplement: Figure 4—source data 5. [file elife-96353-fig4-data5.zip › fig. 4D/input anti-myc right.tif]

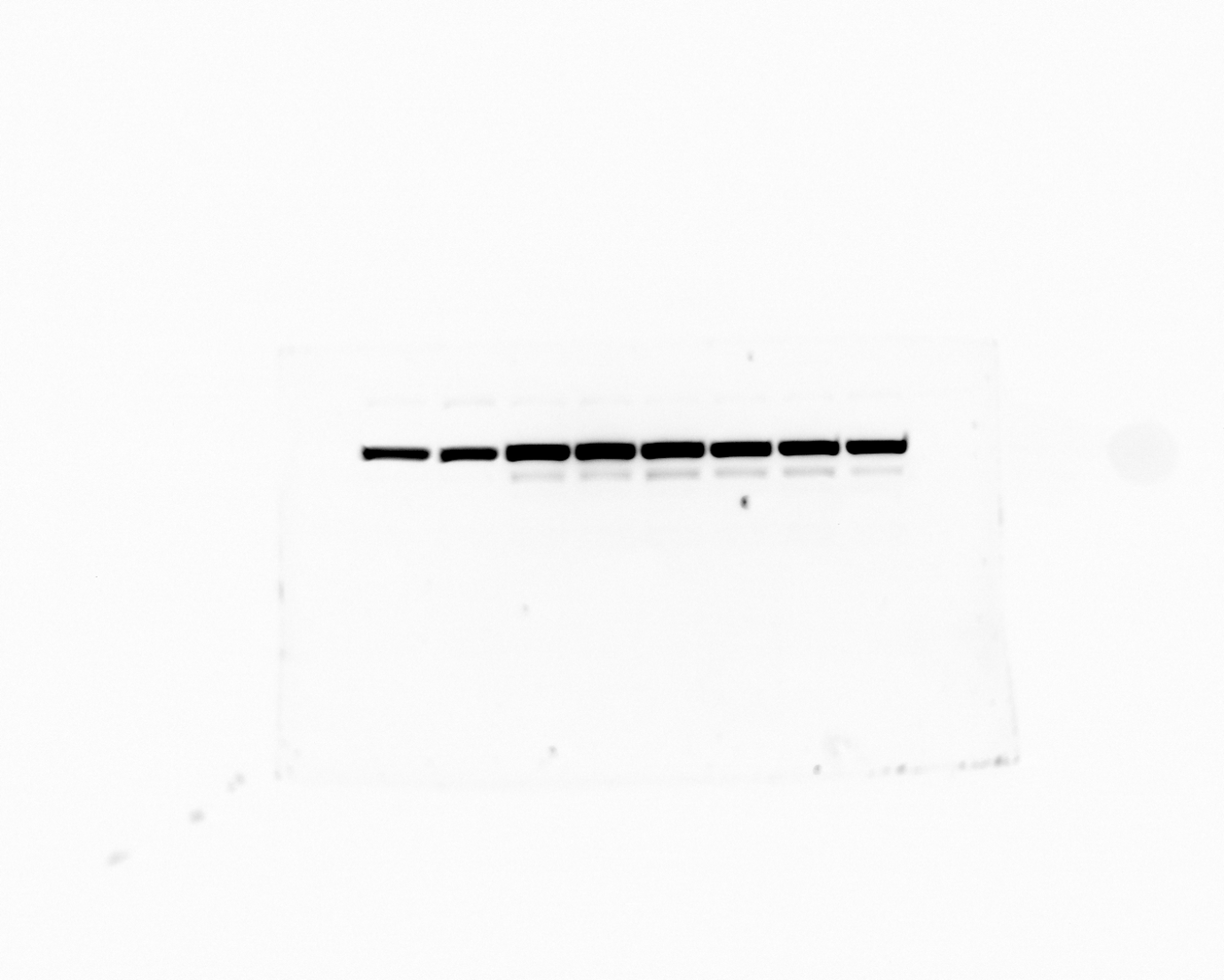

Supplement: Figure 4—source data 5. [file elife-96353-fig4-data5.zip › fig. 4D/input anti-myc right_20s_opt-0007.tif]

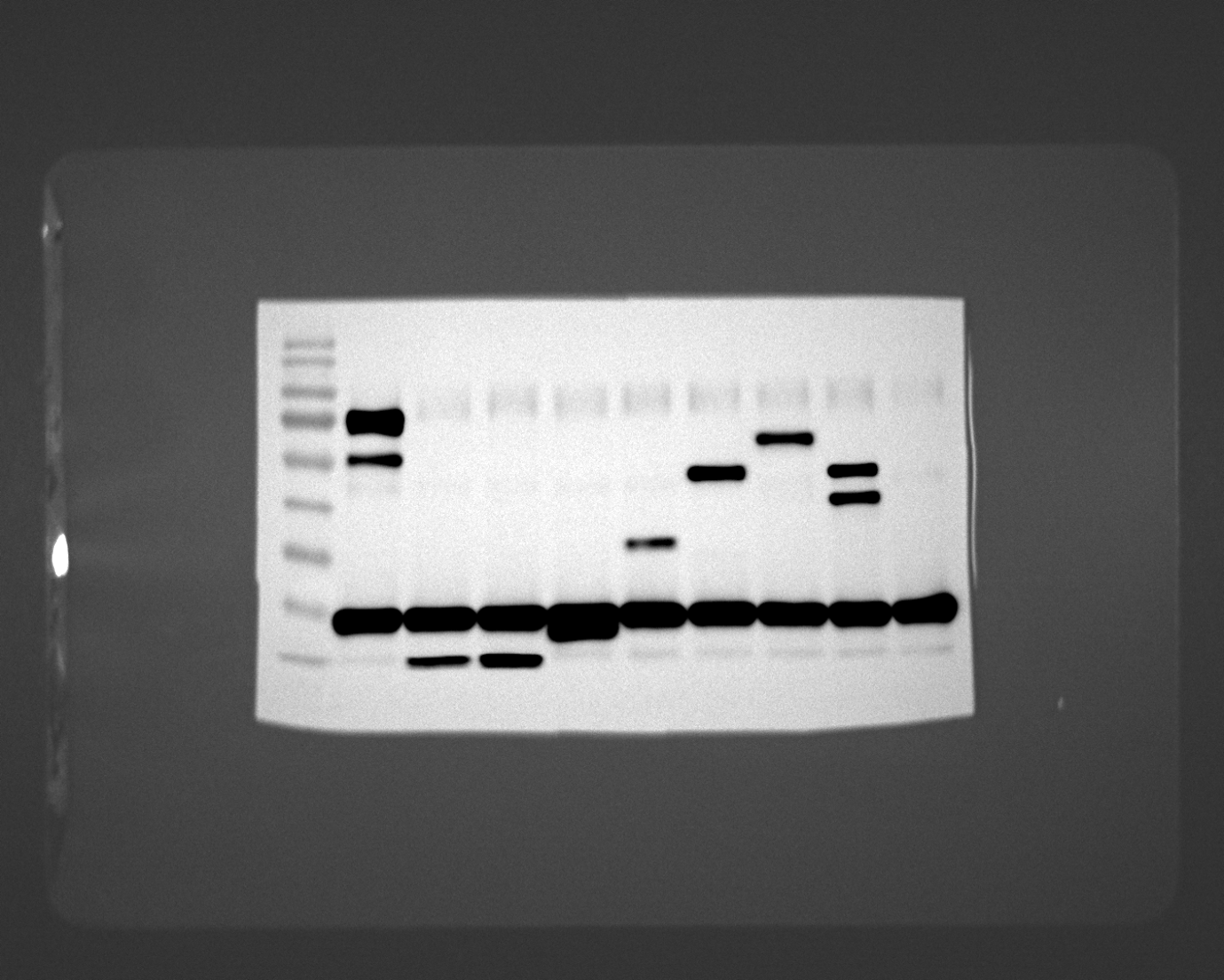

Supplement: Figure 4—source data 5. [file elife-96353-fig4-data5.zip › fig. 4D/IP anti-flag left.tif]

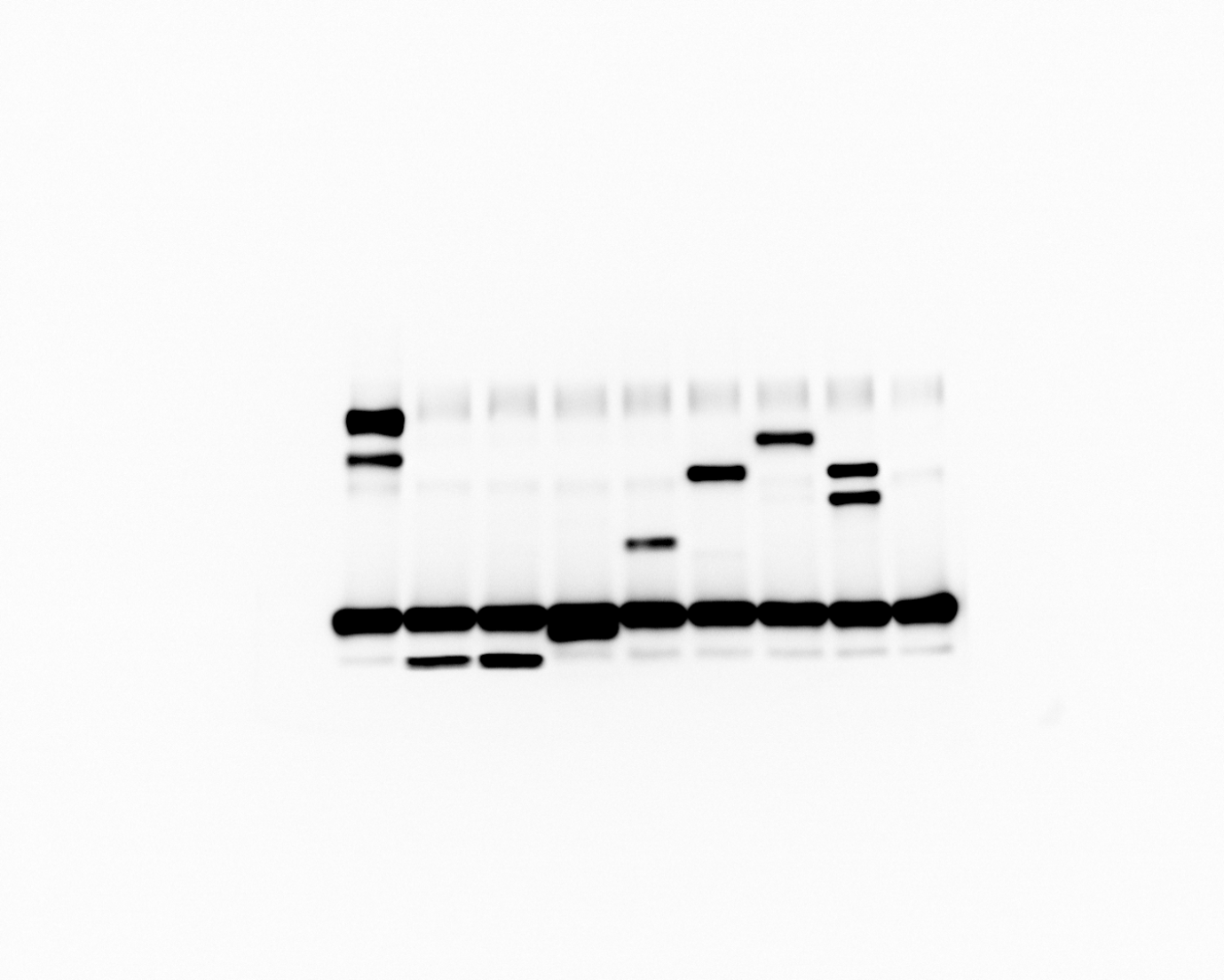

Supplement: Figure 4—source data 5. [file elife-96353-fig4-data5.zip › fig. 4D/IP anti-flag left_1s_opt-0007.tif]

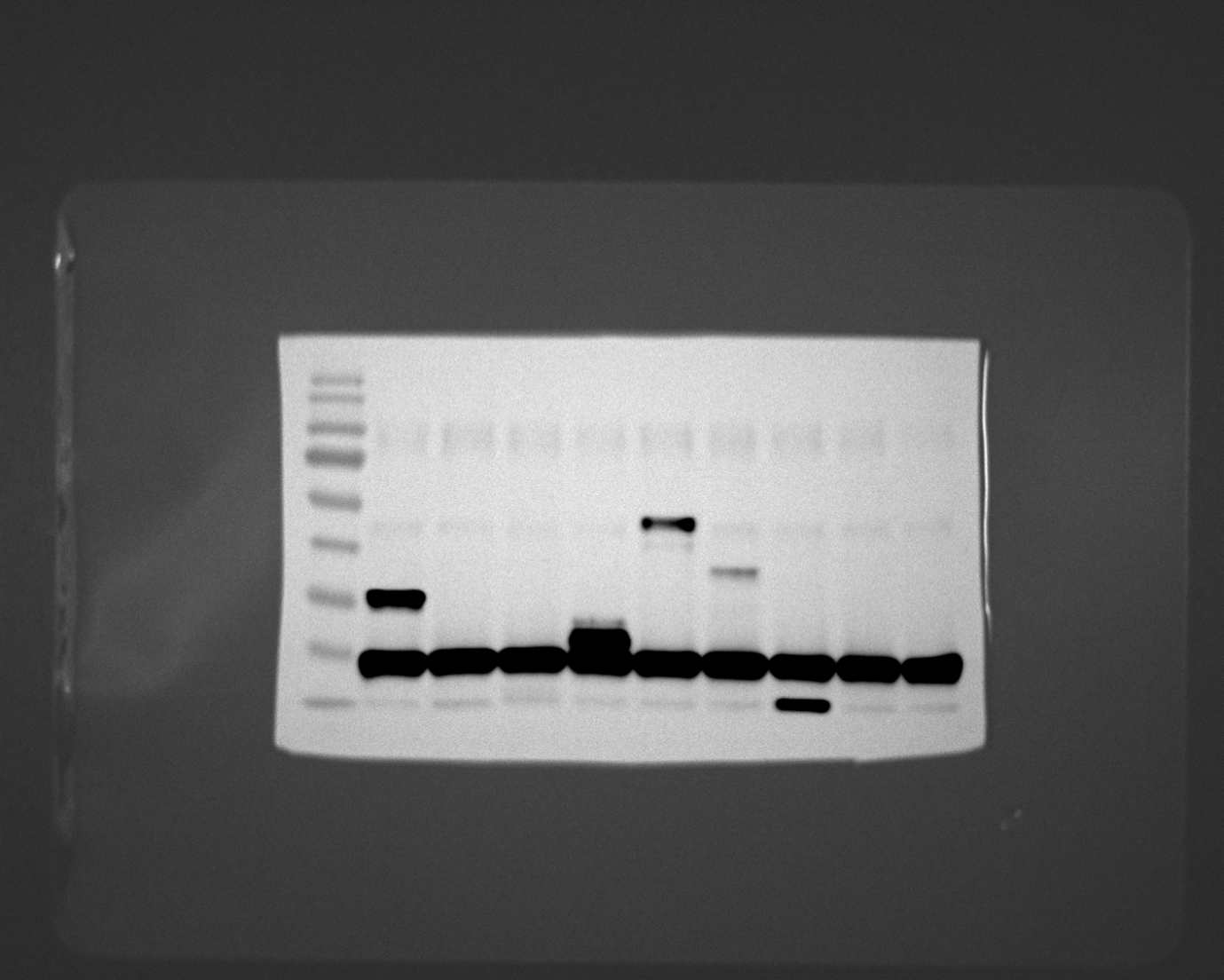

Supplement: Figure 4—source data 5. [file elife-96353-fig4-data5.zip › fig. 4D/IP anti-flag right.tif]

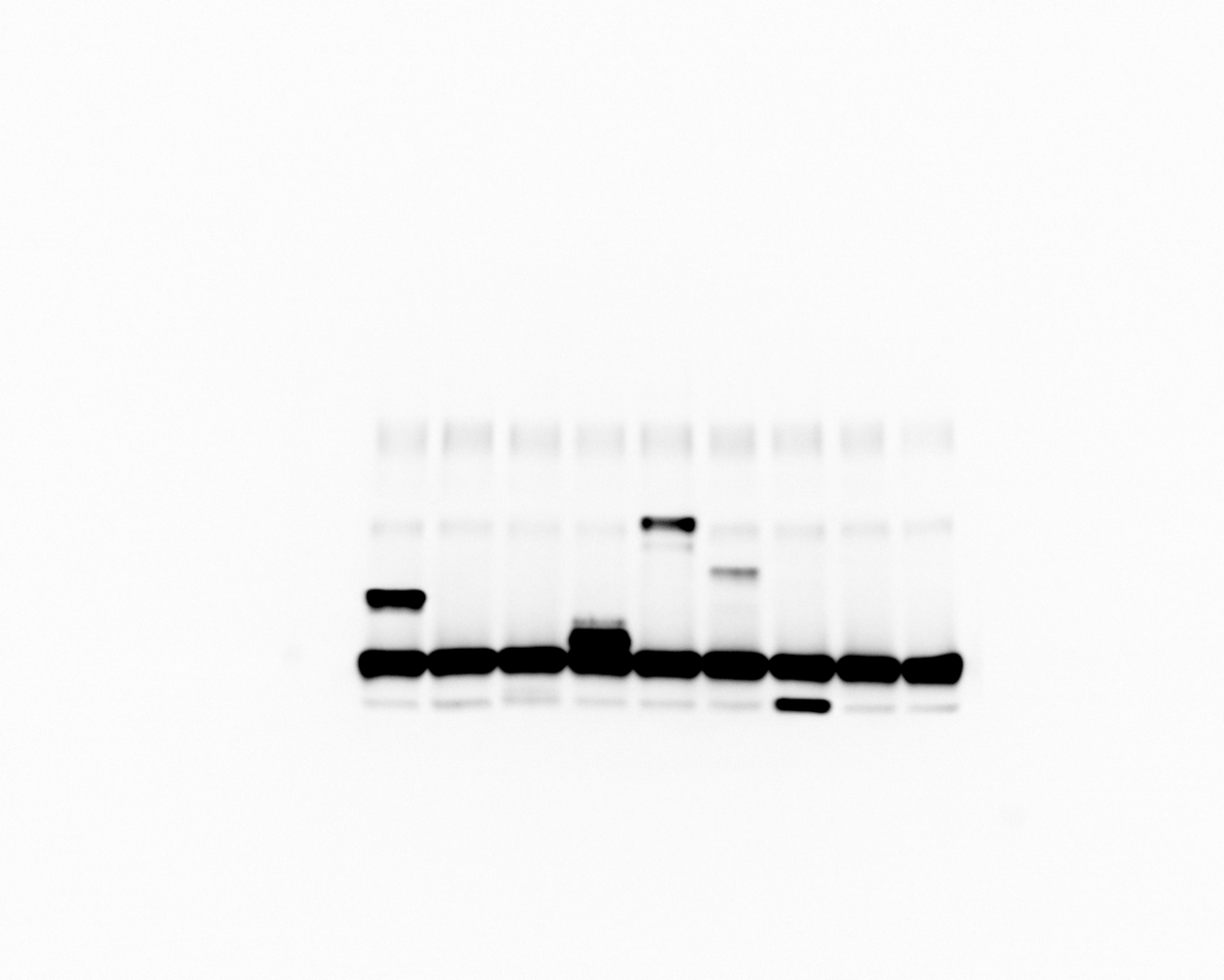

Supplement: Figure 4—source data 5. [file elife-96353-fig4-data5.zip › fig. 4D/IP anti-flag right_1s_opt-0007.tif]

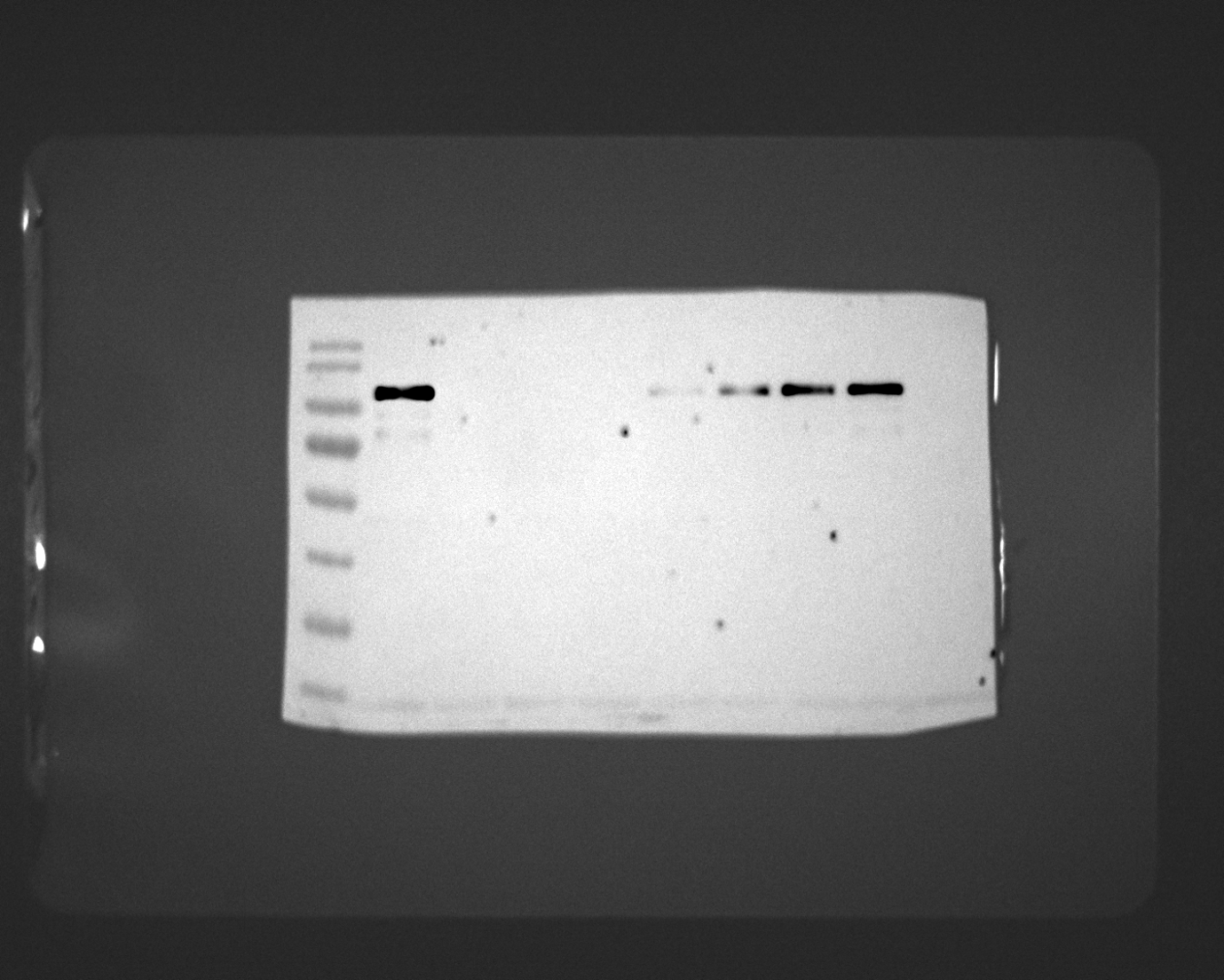

Supplement: Figure 4—source data 5. [file elife-96353-fig4-data5.zip › fig. 4D/IP anti-myc left.tif]

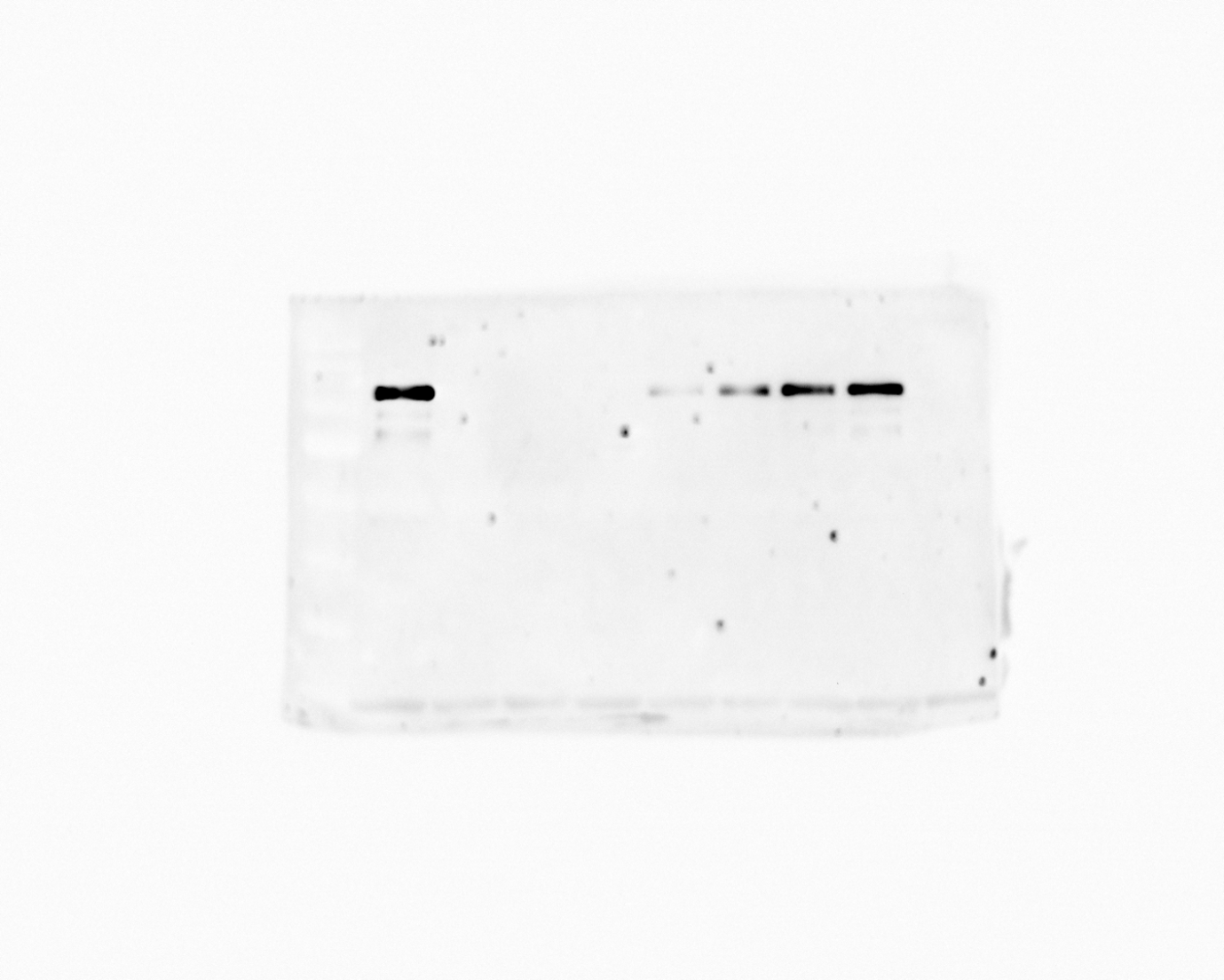

Supplement: Figure 4—source data 5. [file elife-96353-fig4-data5.zip › fig. 4D/IP anti-myc left_10s_opt-0009.tif]

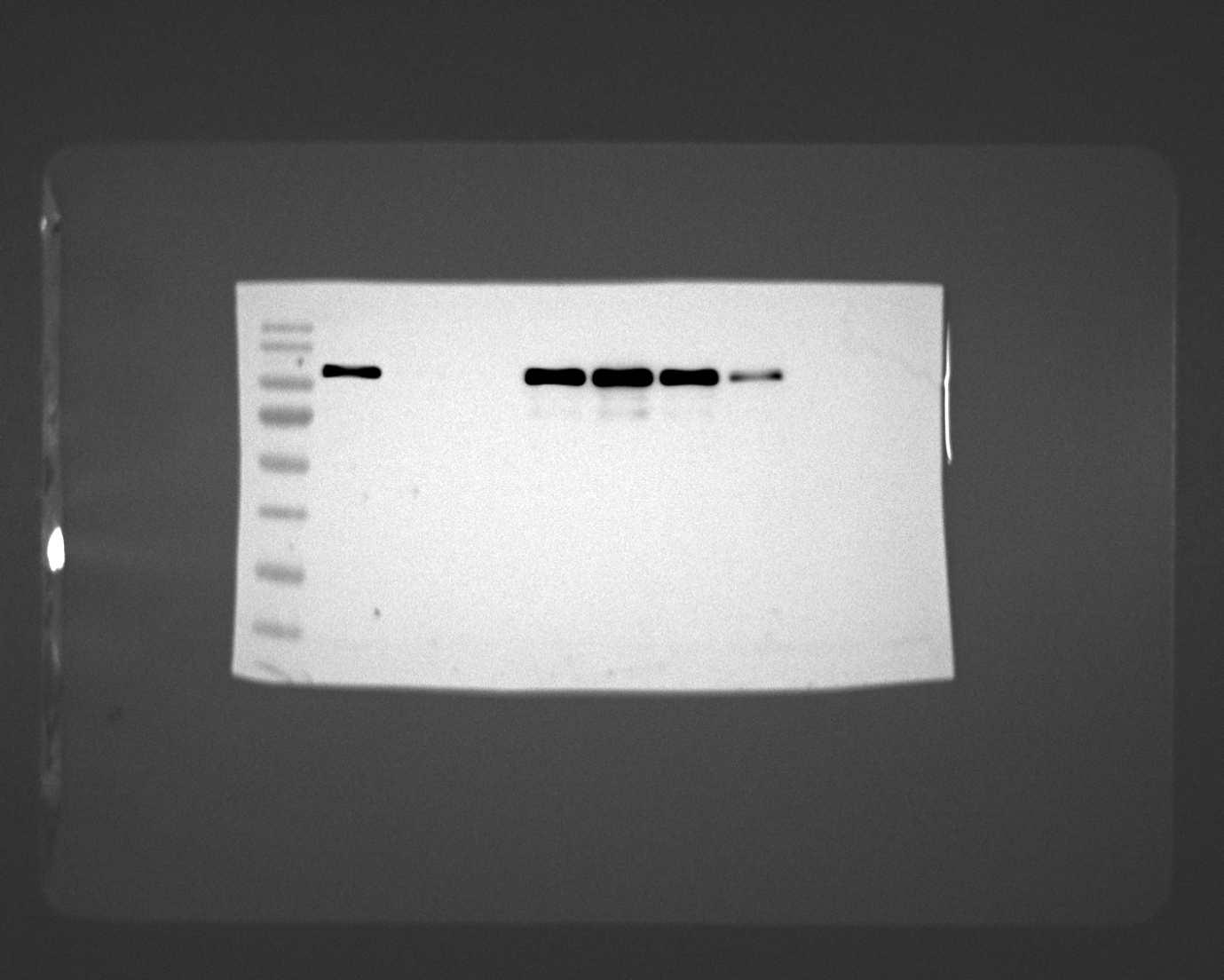

Supplement: Figure 4—source data 5. [file elife-96353-fig4-data5.zip › fig. 4D/IP anti-myc right.tif]

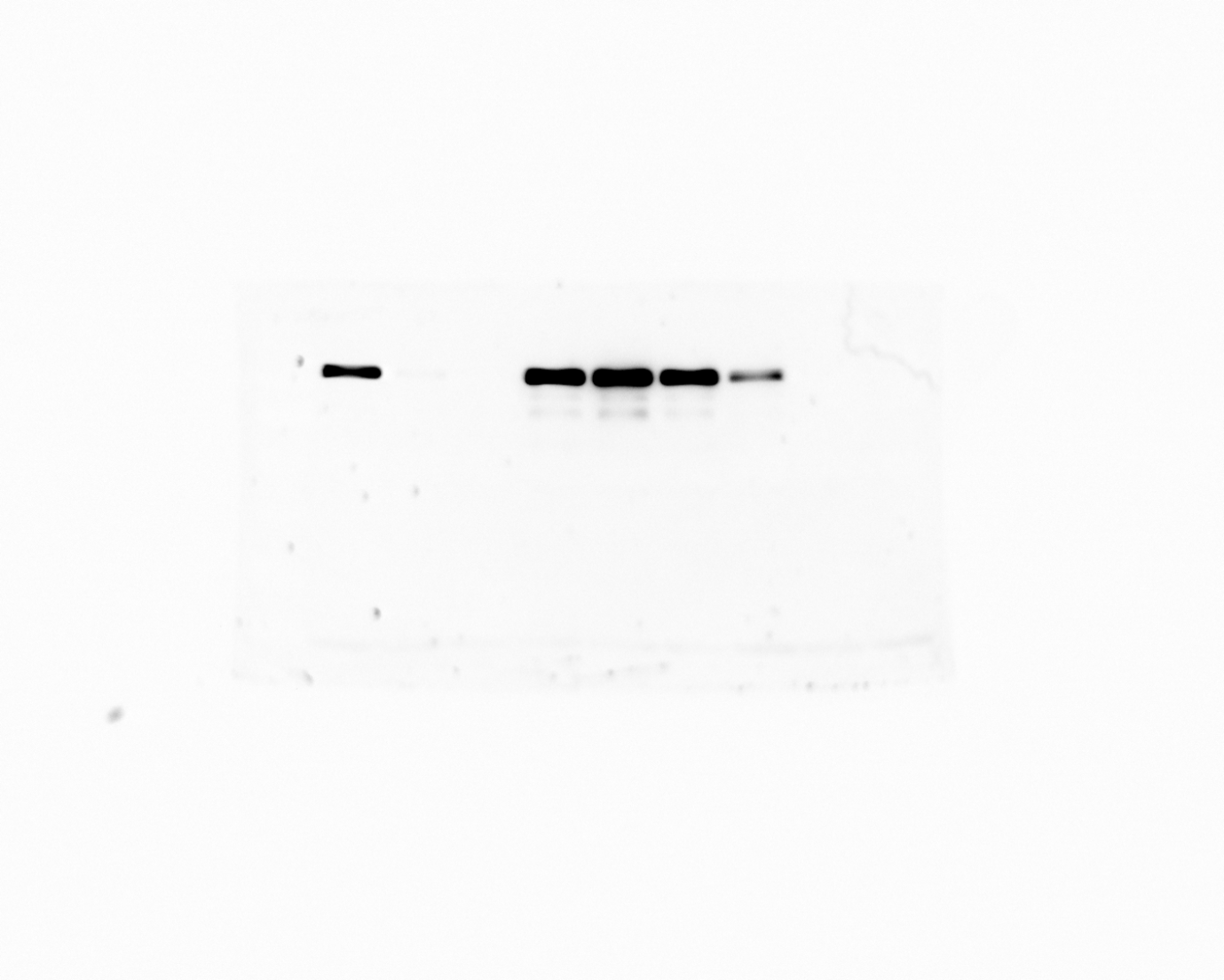

Supplement: Figure 4—source data 5. [file elife-96353-fig4-data5.zip › fig. 4D/IP anti-myc right_10s_opt-0006.tif]

Figure 4D

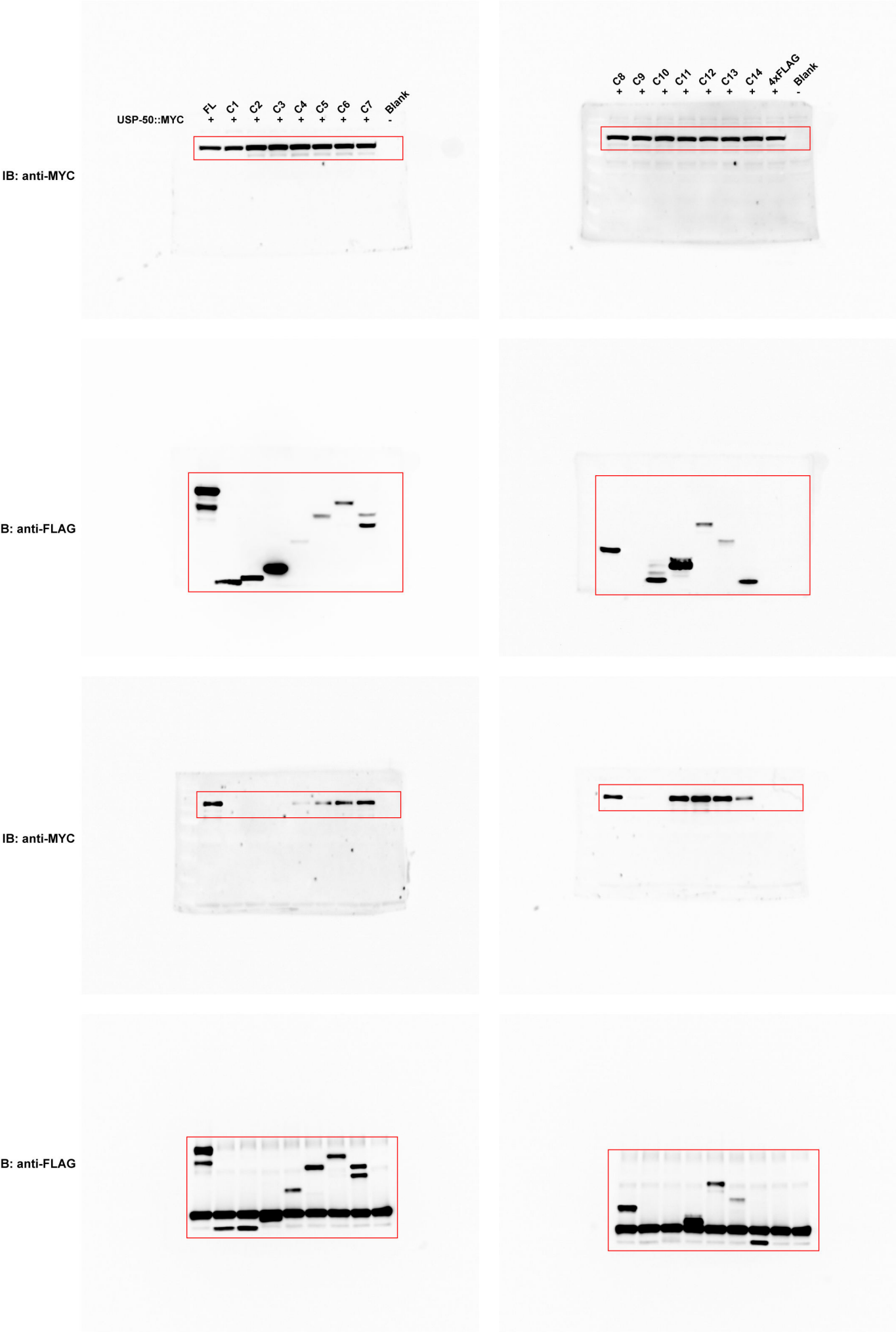

Supplement: Figure 4—source data 6. [file elife-96353-fig4-data6.pdf]

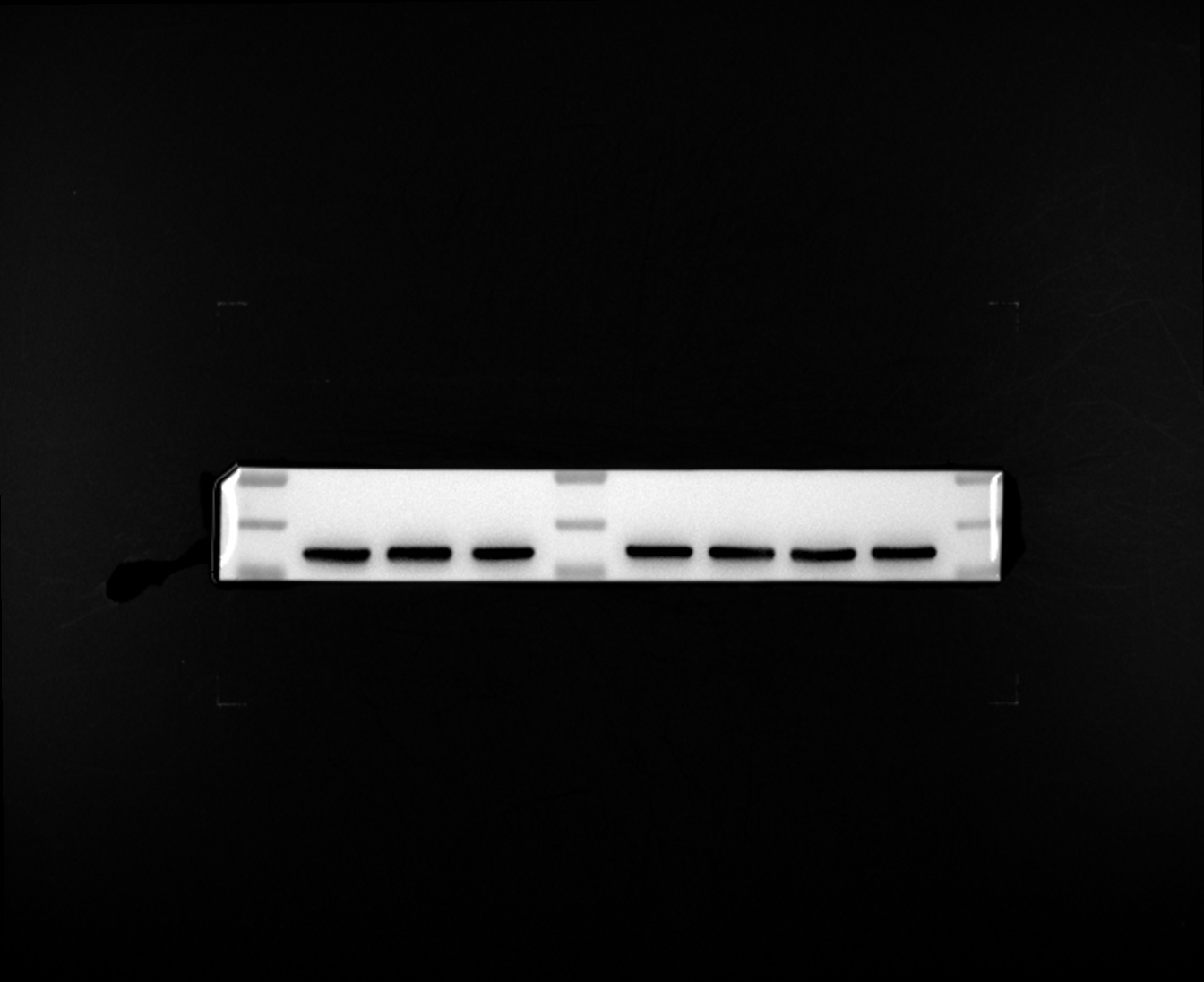

Supplement: Figure 5—figure supplement 1—source data 1. [file elife-96353-fig5-figsupp1-data1.zip › fig. 5-S1E/GAPDH-1.5S+M-1(fig5-s1).tif]

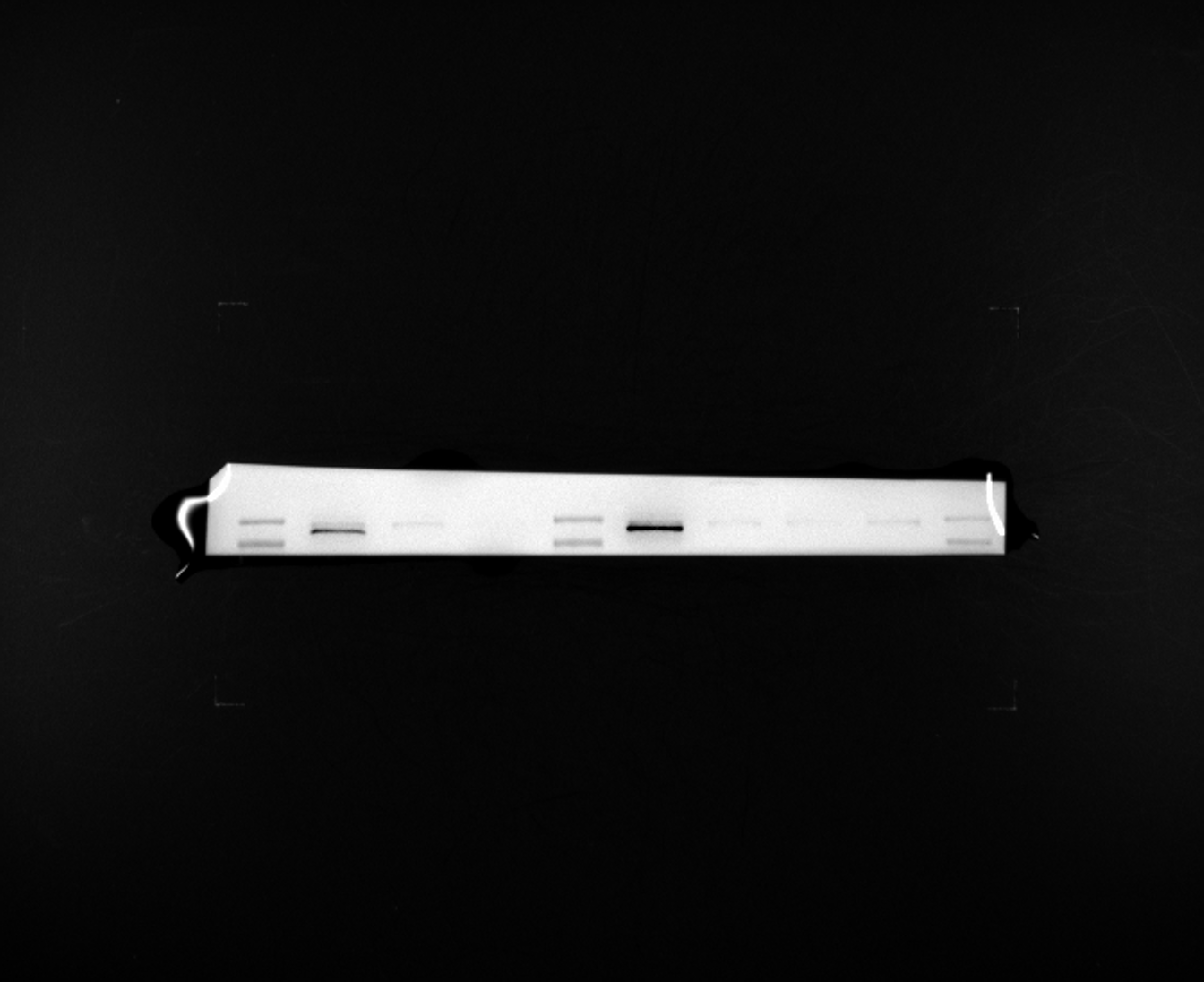

Supplement: Figure 5—figure supplement 1—source data 1. [file elife-96353-fig5-figsupp1-data1.zip › fig. 5-S1E/UBPY-1.5S+M(fig5-s1).Tif]

**Figure 5-S1E**

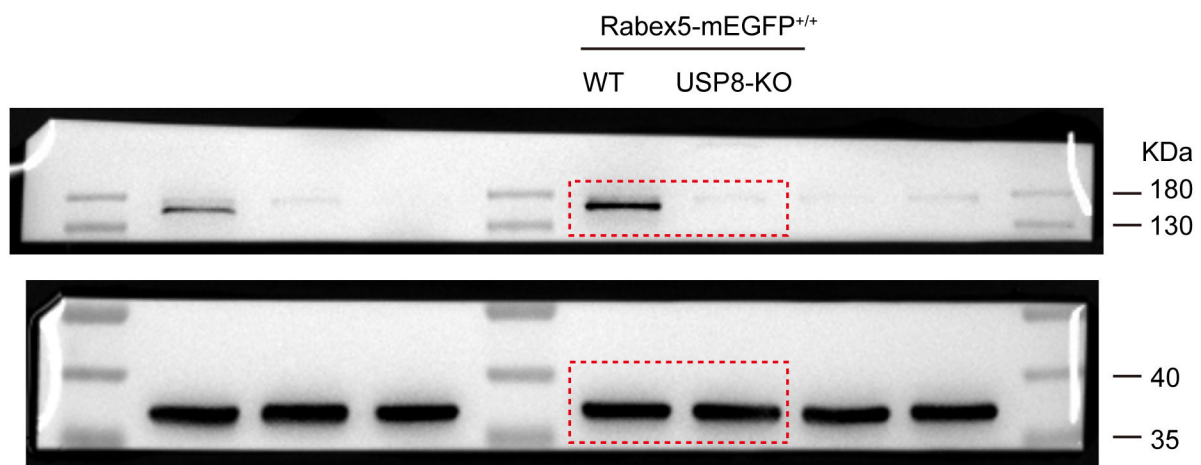

Supplement: Figure 5—figure supplement 1—source data 2. [file elife-96353-fig5-figsupp1-data2.pdf]

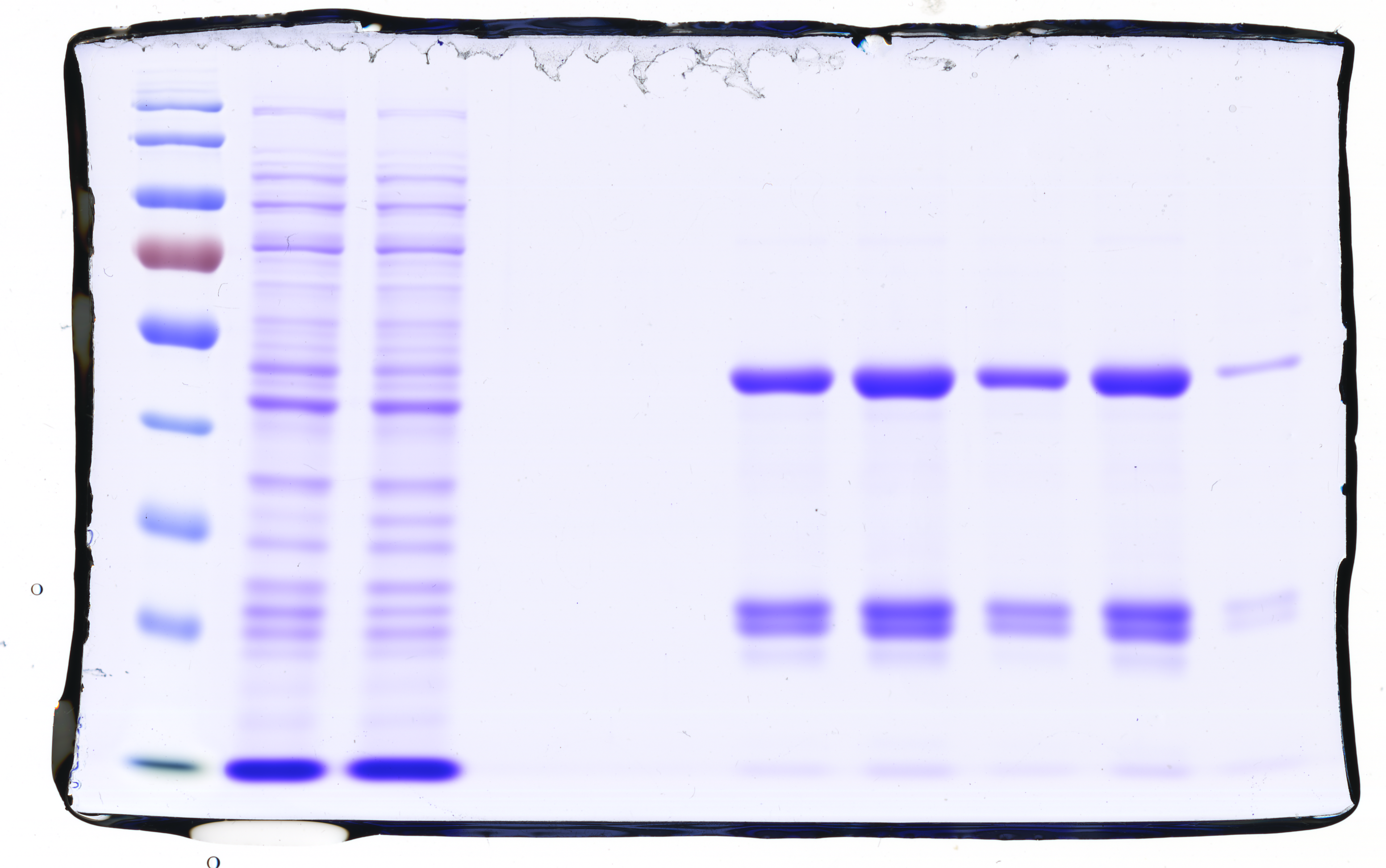

Supplement: Figure 5—figure supplement 2—source data 1. [file elife-96353-fig5-figsupp2-data1.zip › fig. 5-S2E/20200722.tif]

Figure 5-S2E

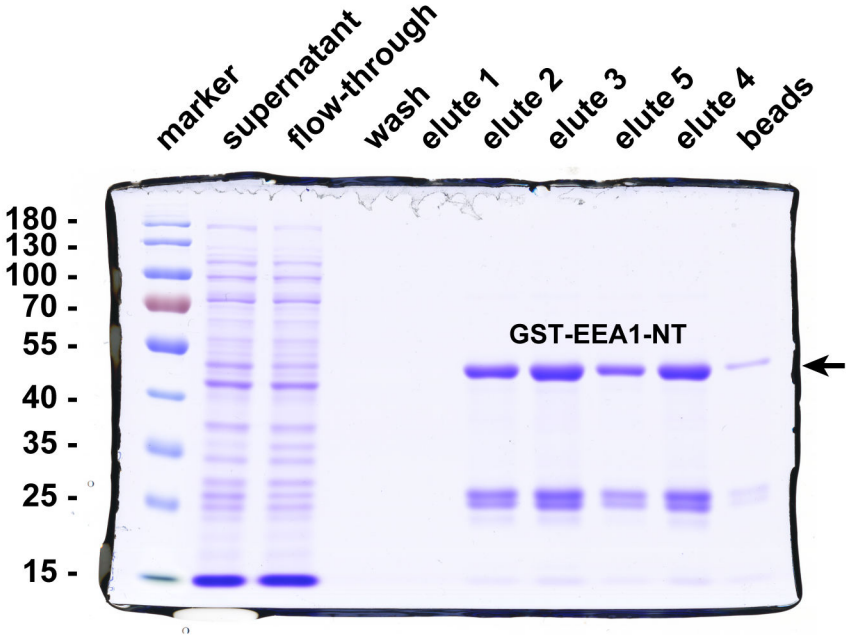

Supplement: Figure 5—figure supplement 2—source data 2. [file elife-96353-fig5-figsupp2-data2.pdf]

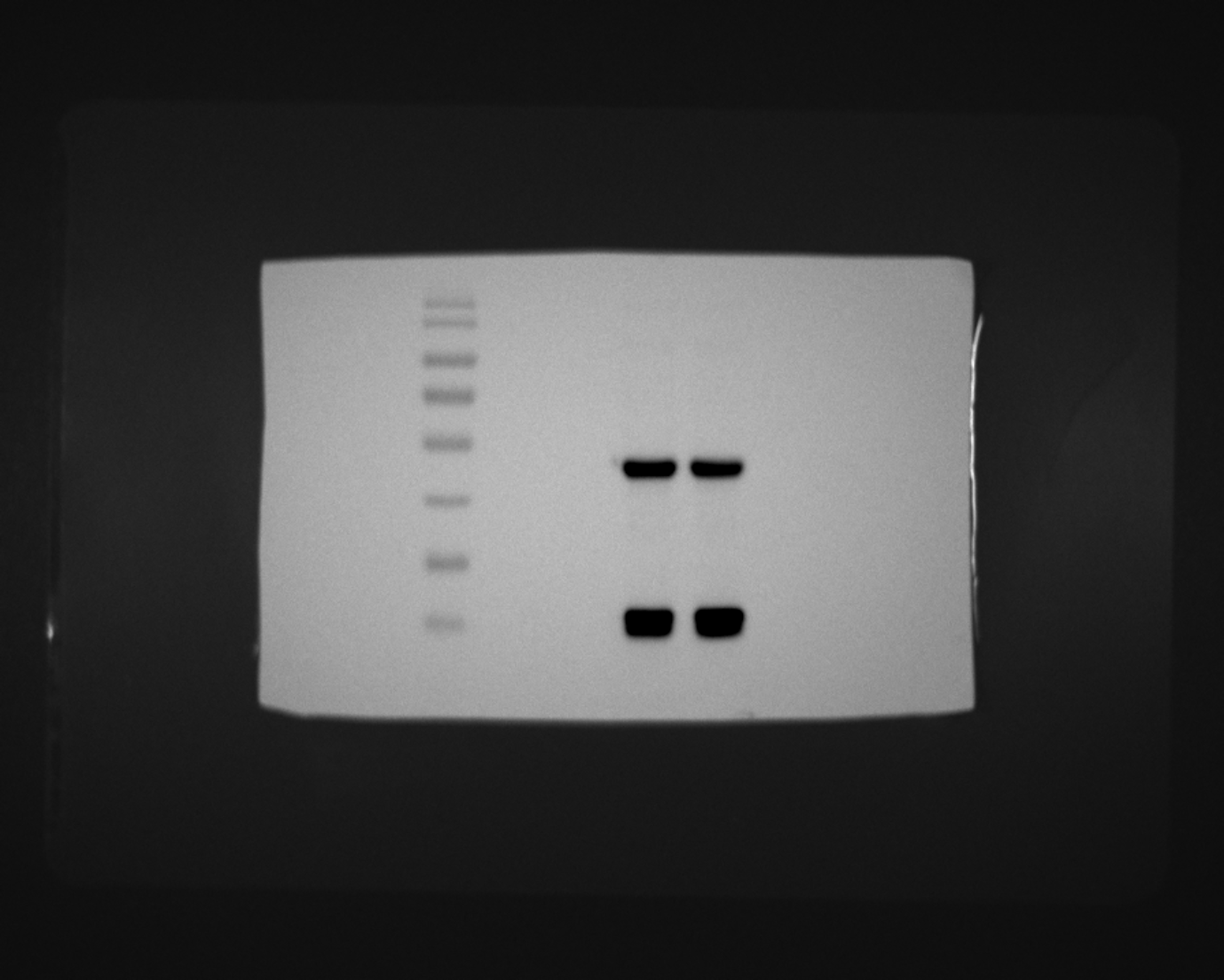

Supplement: Figure 5—figure supplement 2—source data 3. [file elife-96353-fig5-figsupp2-data3.zip › fig. 5-S2F/anti-gst.tif]

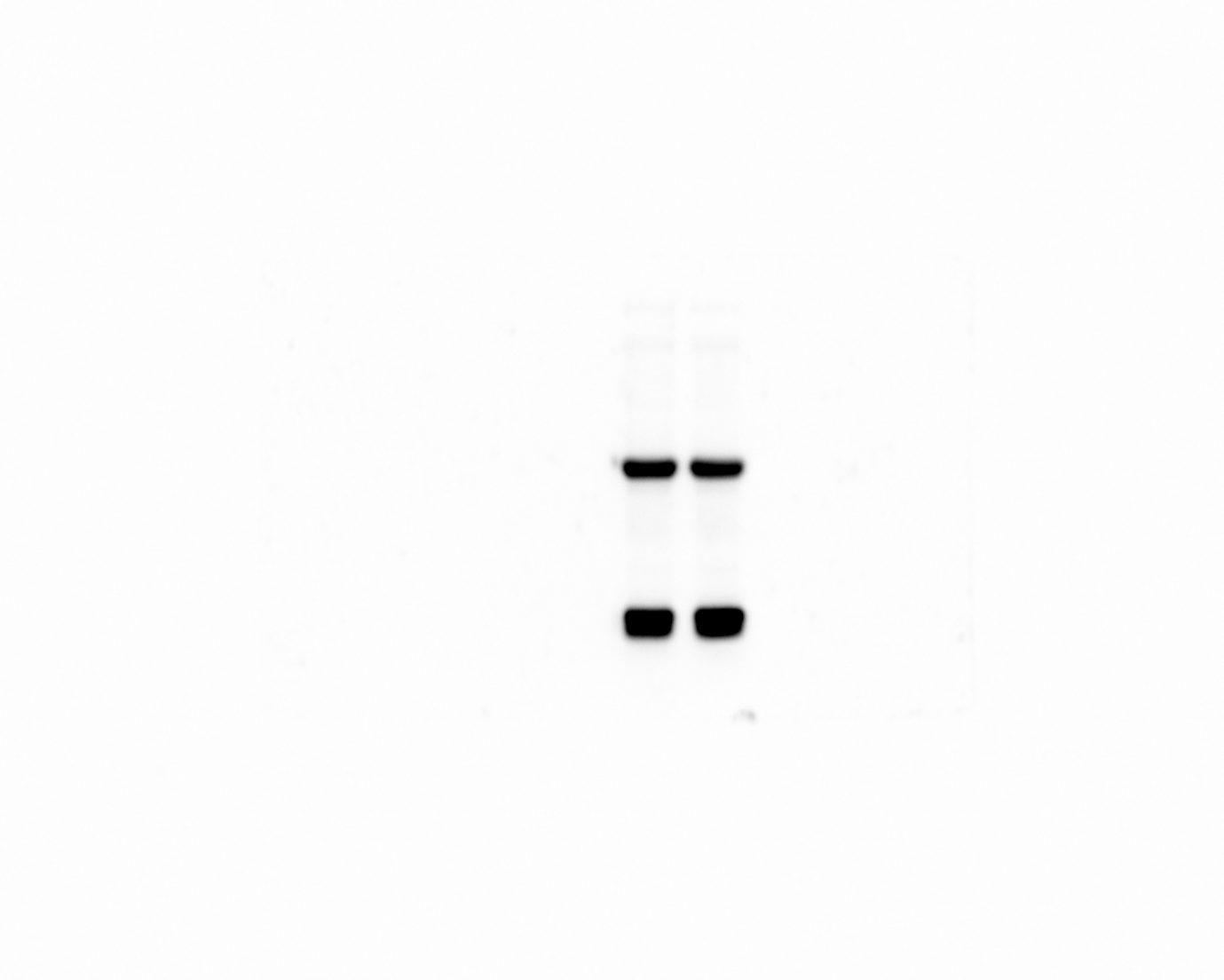

Supplement: Figure 5—figure supplement 2—source data 3. [file elife-96353-fig5-figsupp2-data3.zip › fig. 5-S2F/anti-gst_1s-0009.tif]

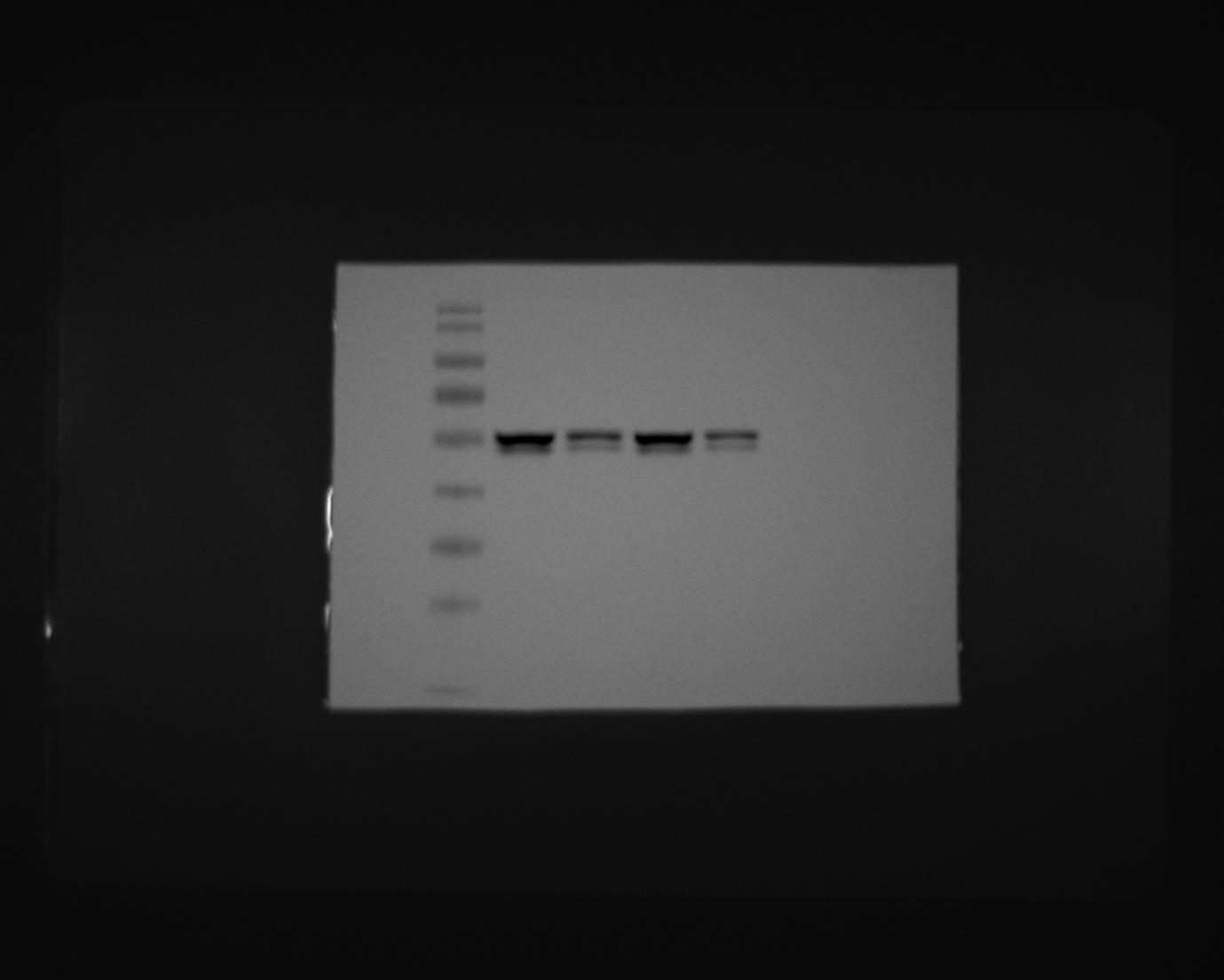

Supplement: Figure 5—figure supplement 2—source data 3. [file elife-96353-fig5-figsupp2-data3.zip › fig. 5-S2F/input anti-gfp.tif]

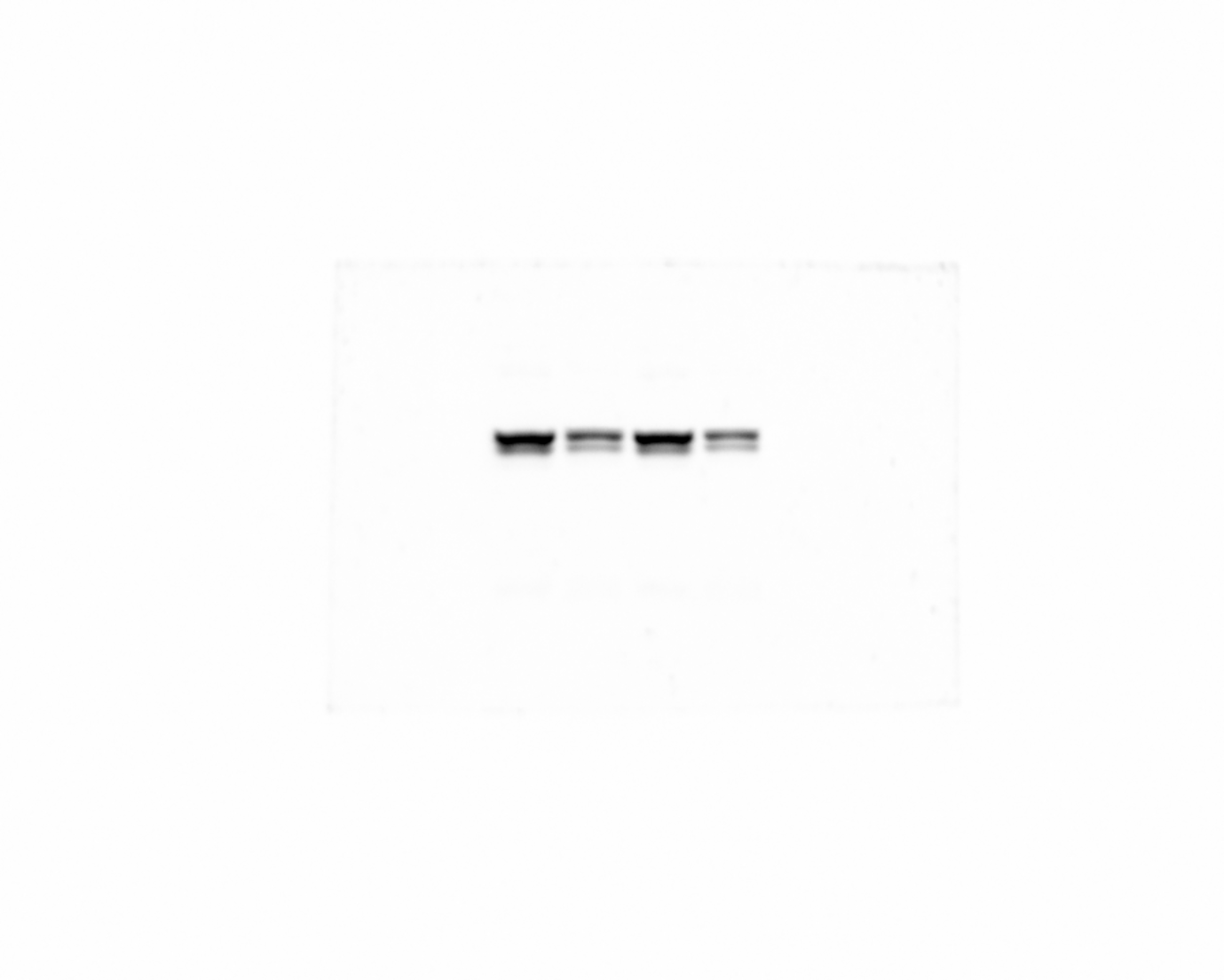

Supplement: Figure 5—figure supplement 2—source data 3. [file elife-96353-fig5-figsupp2-data3.zip › fig. 5-S2F/input anti-gfp_1s-0004.tif]

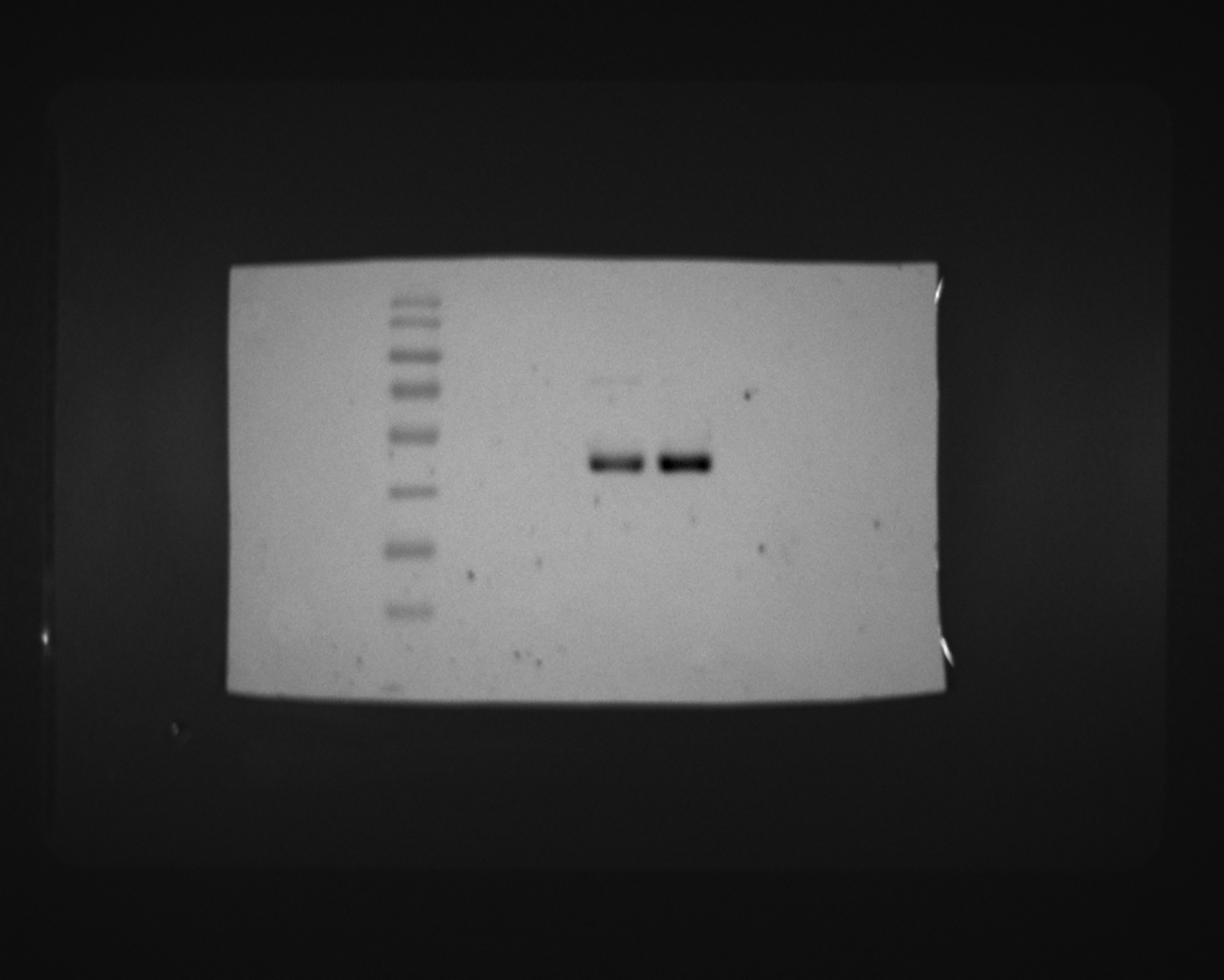

Supplement: Figure 5—figure supplement 2—source data 3. [file elife-96353-fig5-figsupp2-data3.zip › fig. 5-S2F/pull down anti-gfp.tif]

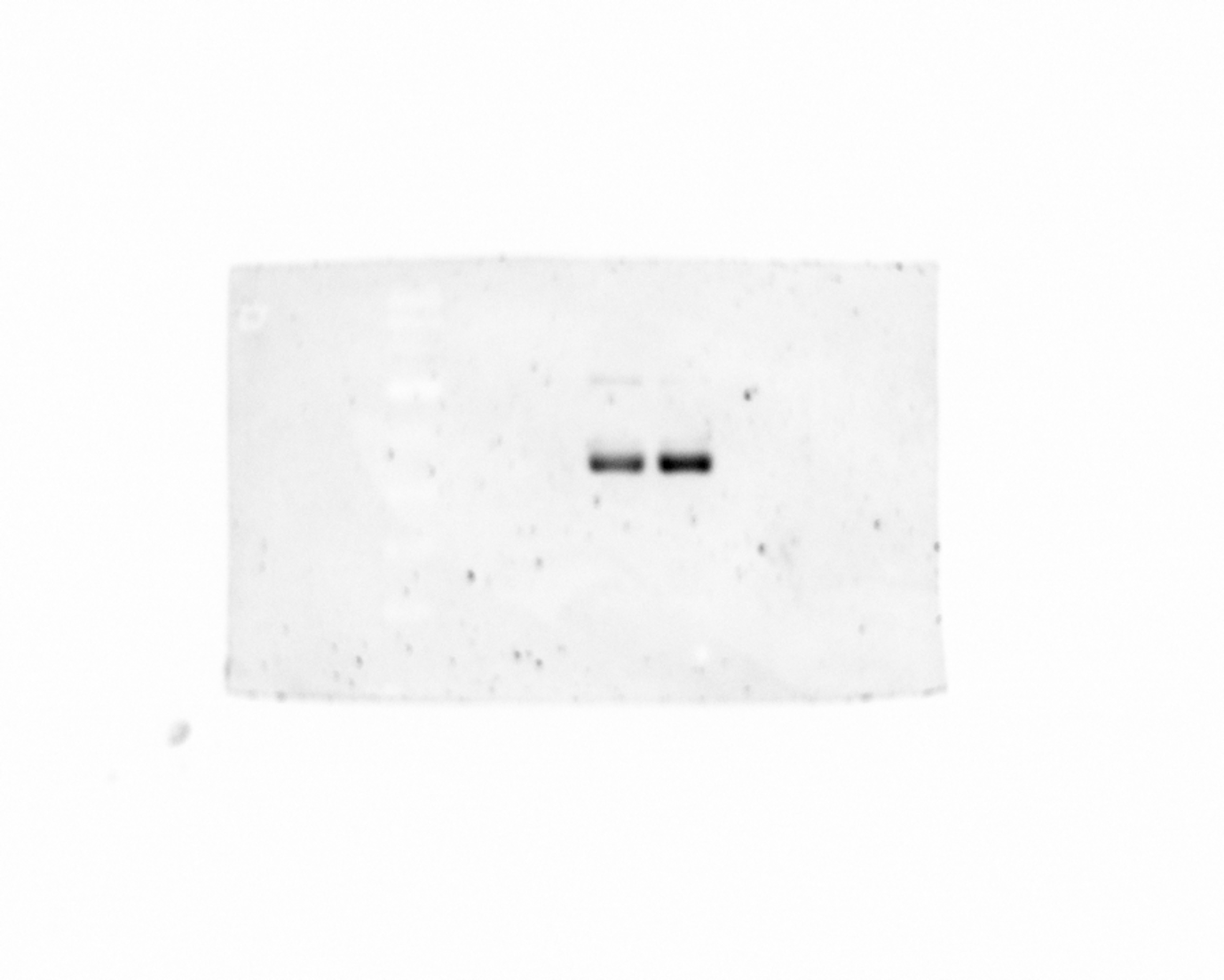

Supplement: Figure 5—figure supplement 2—source data 3. [file elife-96353-fig5-figsupp2-data3.zip › fig. 5-S2F/pull down anti-gfp_10s-0001.tif]

Figure 5-S2F

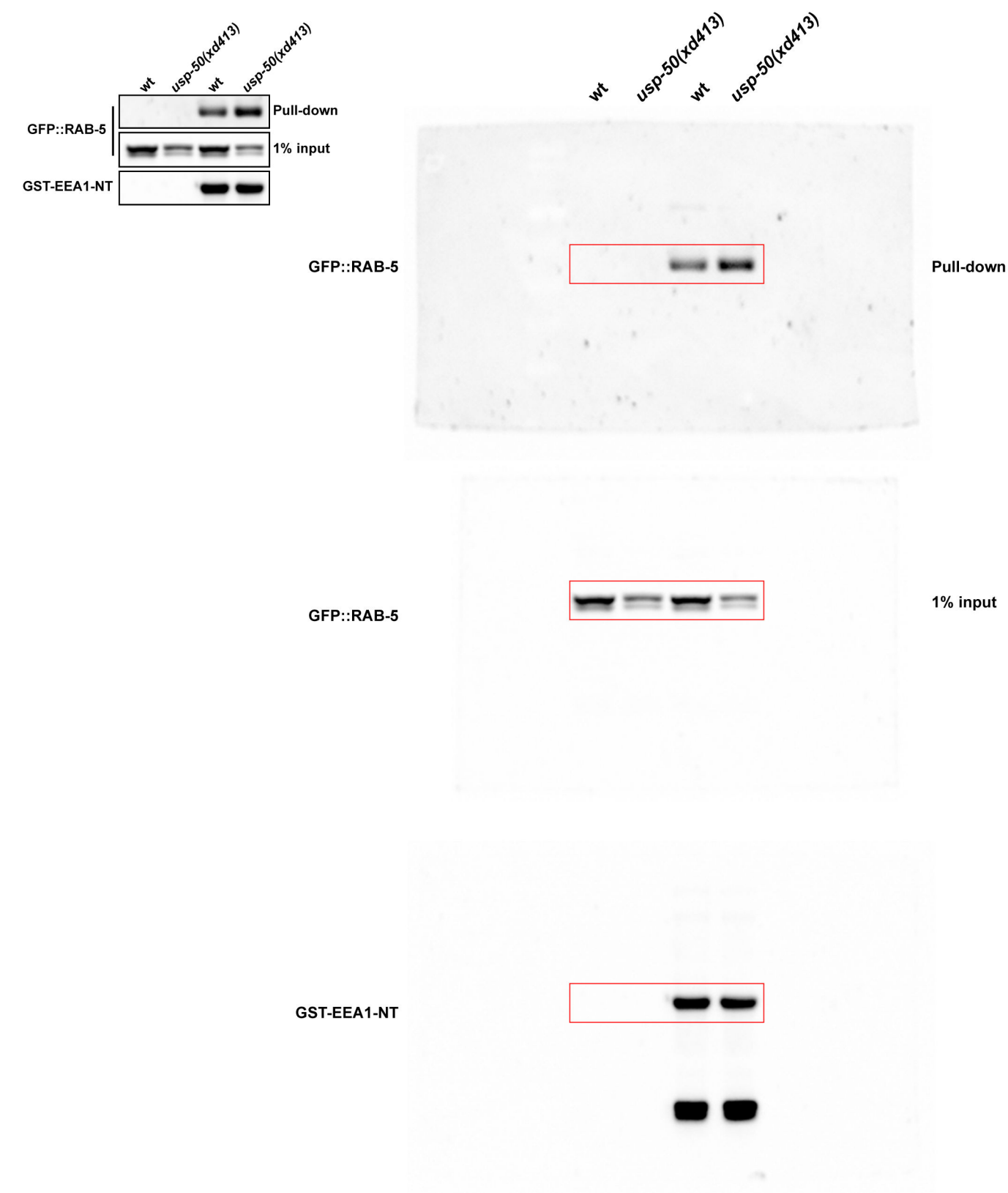

Supplement: Figure 5—figure supplement 2—source data 4. [file elife-96353-fig5-figsupp2-data4.pdf]

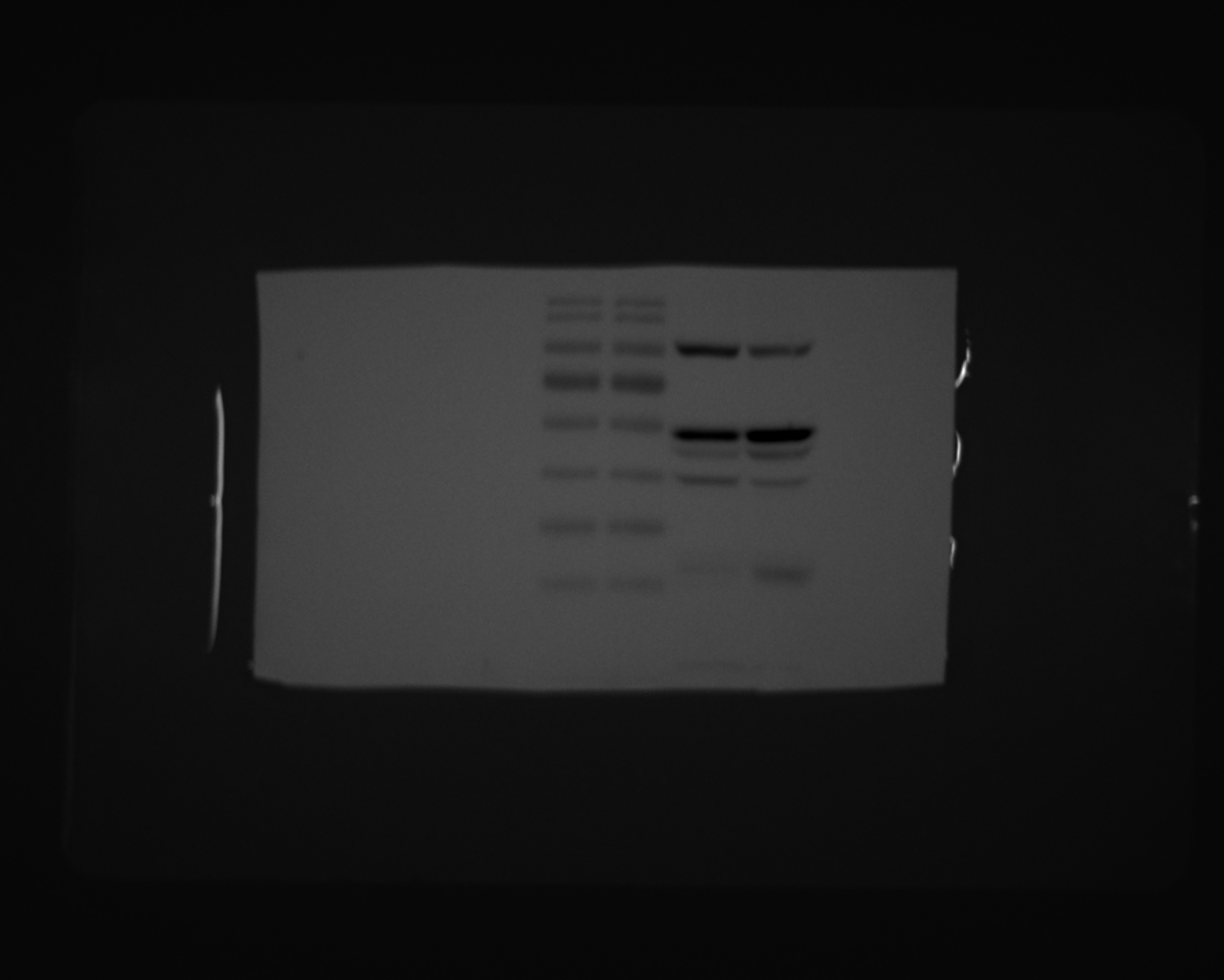

Supplement: Figure 5—figure supplement 2—source data 5. [file elife-96353-fig5-figsupp2-data5.zip › fig. 5-S2G/anti-gfp.tif]

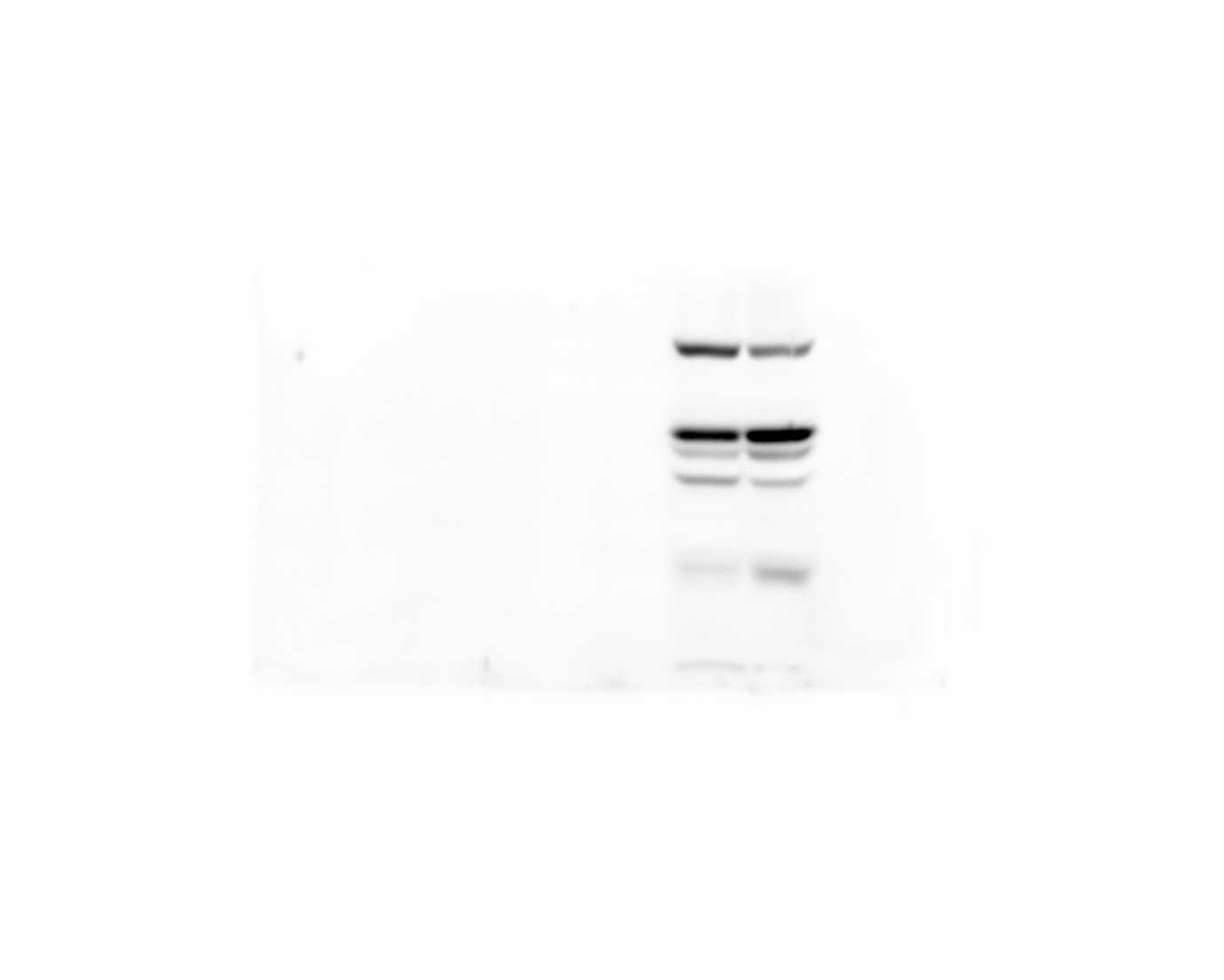

Supplement: Figure 5—figure supplement 2—source data 5. [file elife-96353-fig5-figsupp2-data5.zip › fig. 5-S2G/anti-gfp_500ms-0006.tif]

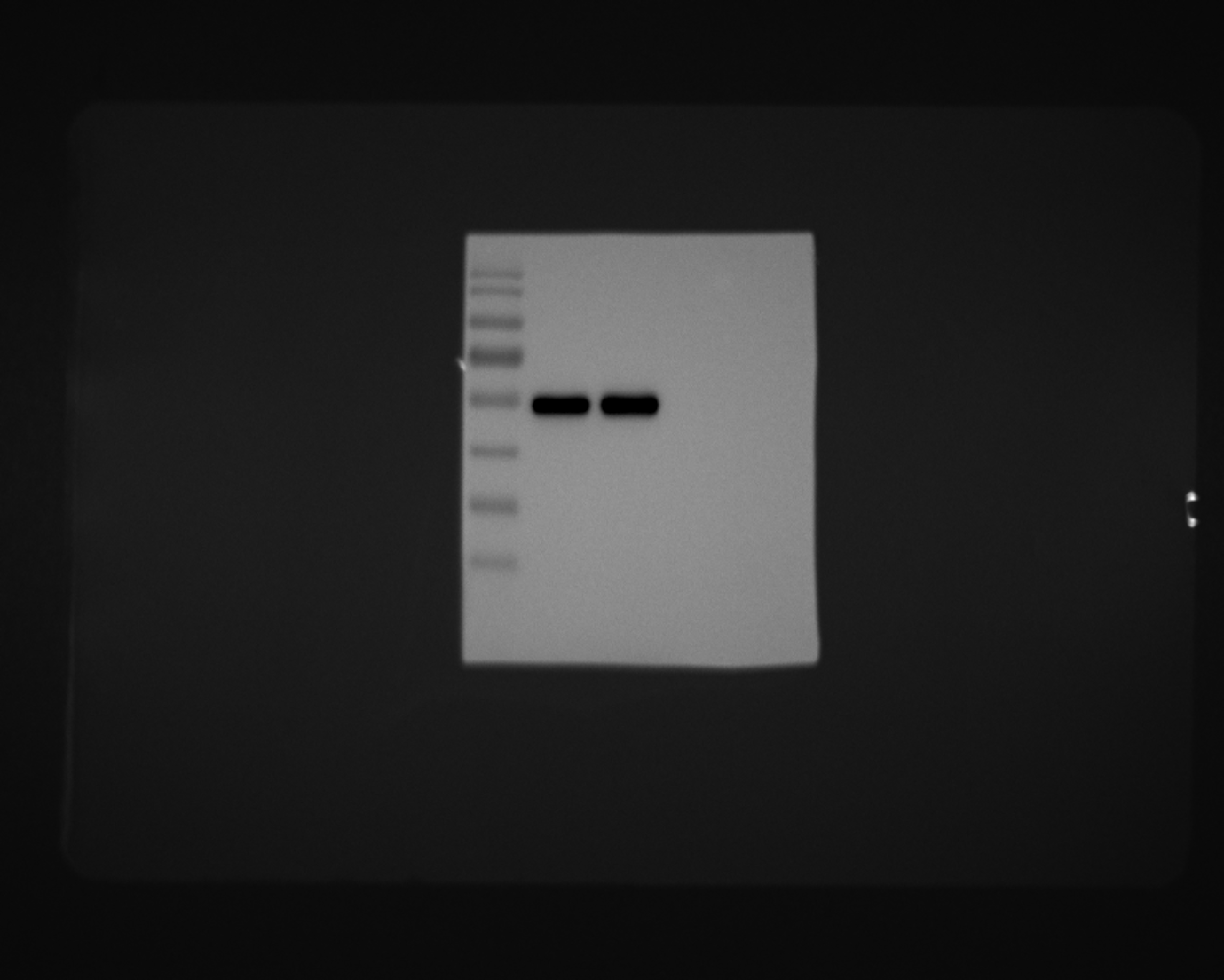

Supplement: Figure 5—figure supplement 2—source data 5. [file elife-96353-fig5-figsupp2-data5.zip › fig. 5-S2G/anti-tub.tif]

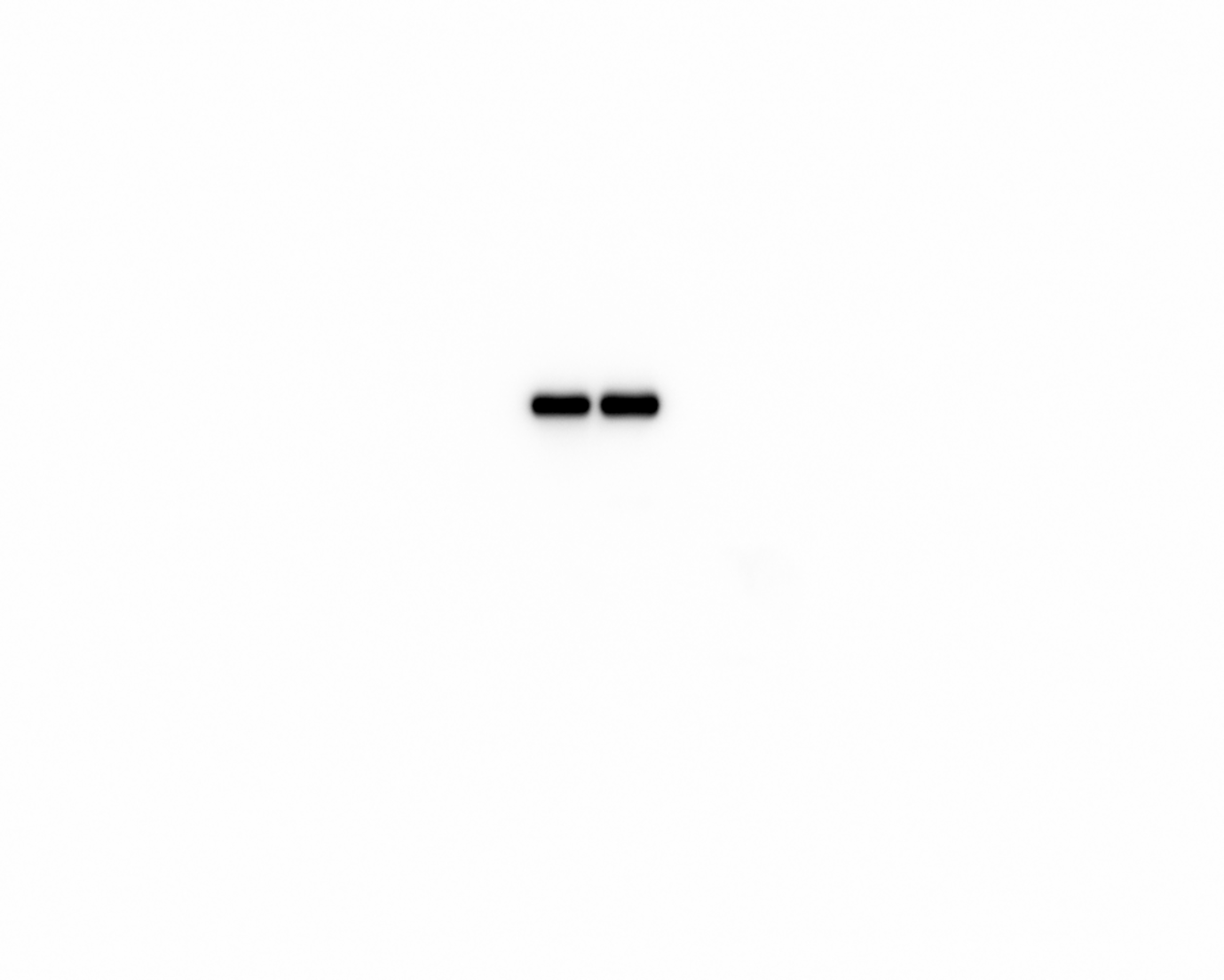

Supplement: Figure 5—figure supplement 2—source data 5. [file elife-96353-fig5-figsupp2-data5.zip › fig. 5-S2G/anti-tub_50ms-0005.tif]

Figure 5-S2G

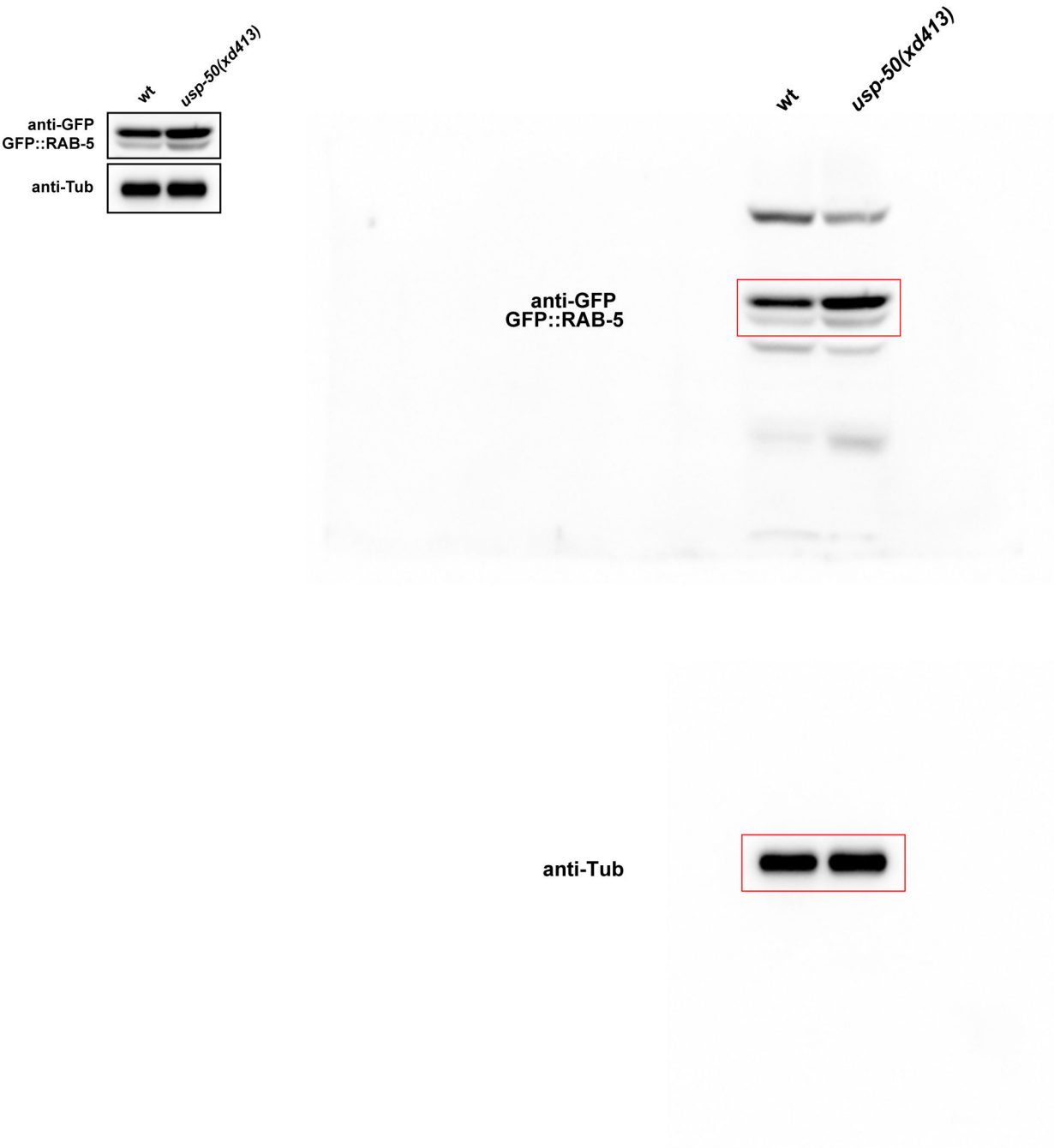

Supplement: Figure 5—figure supplement 2—source data 6. [file elife-96353-fig5-figsupp2-data6.pdf]

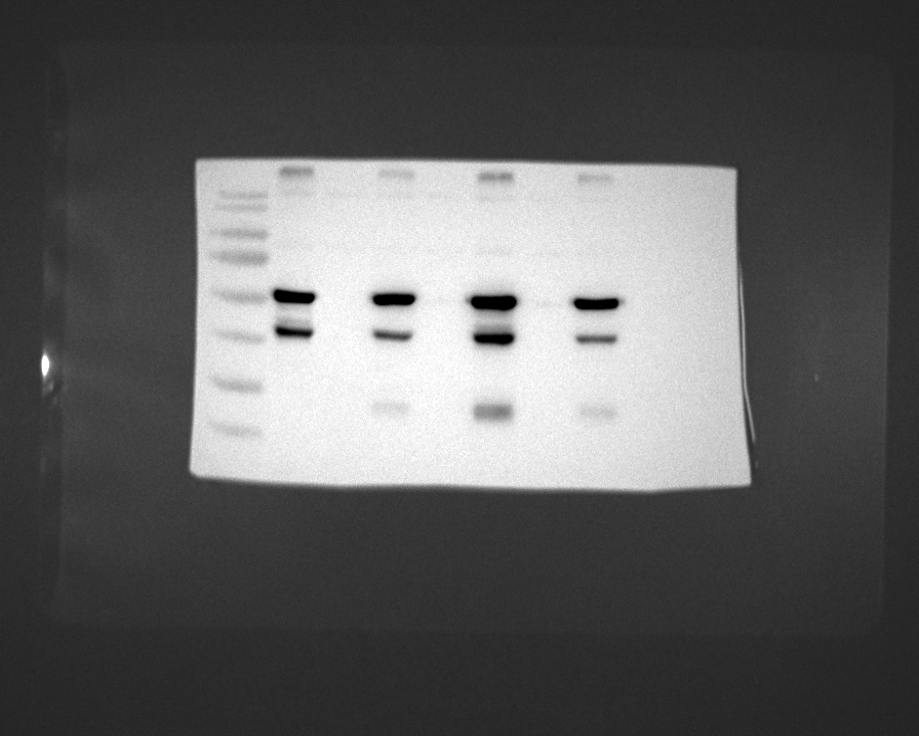

Supplement: Figure 6—source data 1. [file elife-96353-fig6-data1.zip › fig. 6P/input anti-flag.tif]

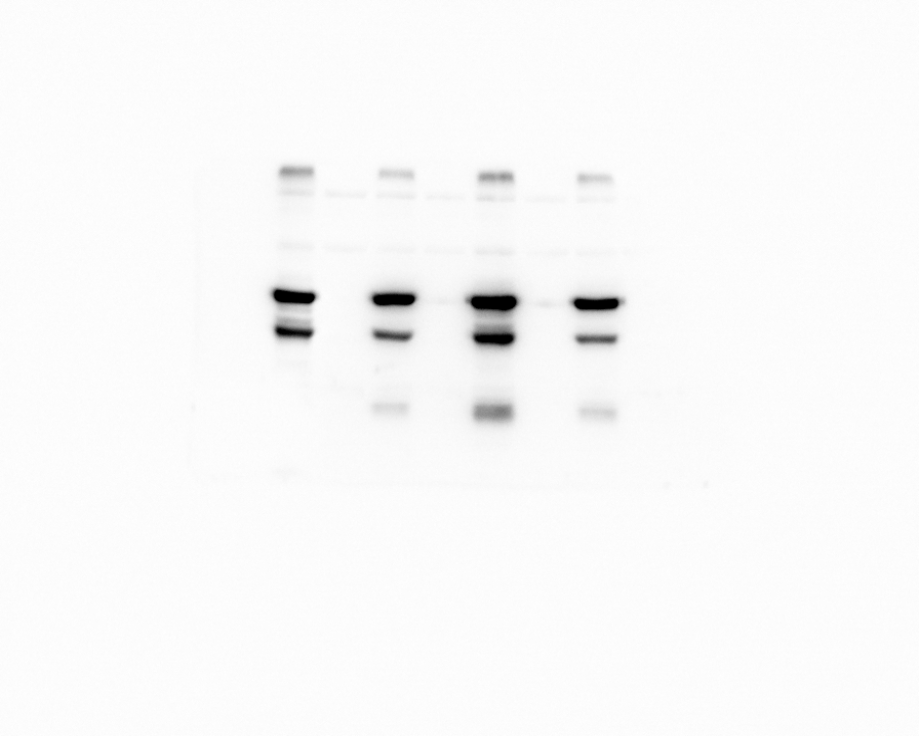

Supplement: Figure 6—source data 1. [file elife-96353-fig6-data1.zip › fig. 6P/input anti-flag_1s_opt-0005.tif]

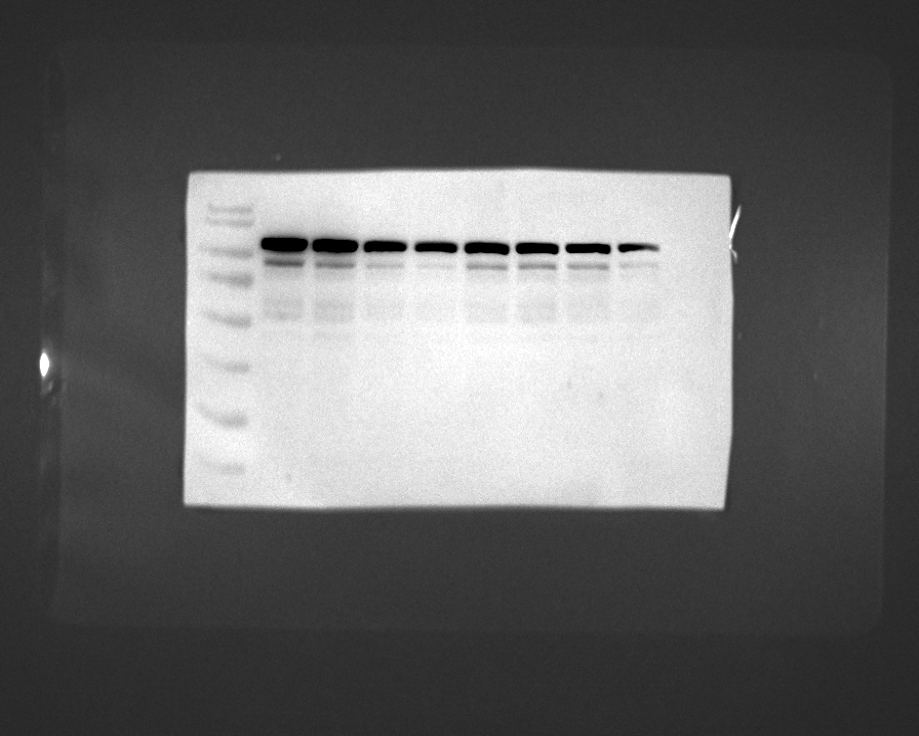

Supplement: Figure 6—source data 1. [file elife-96353-fig6-data1.zip › fig. 6P/input anti-myc.tif]

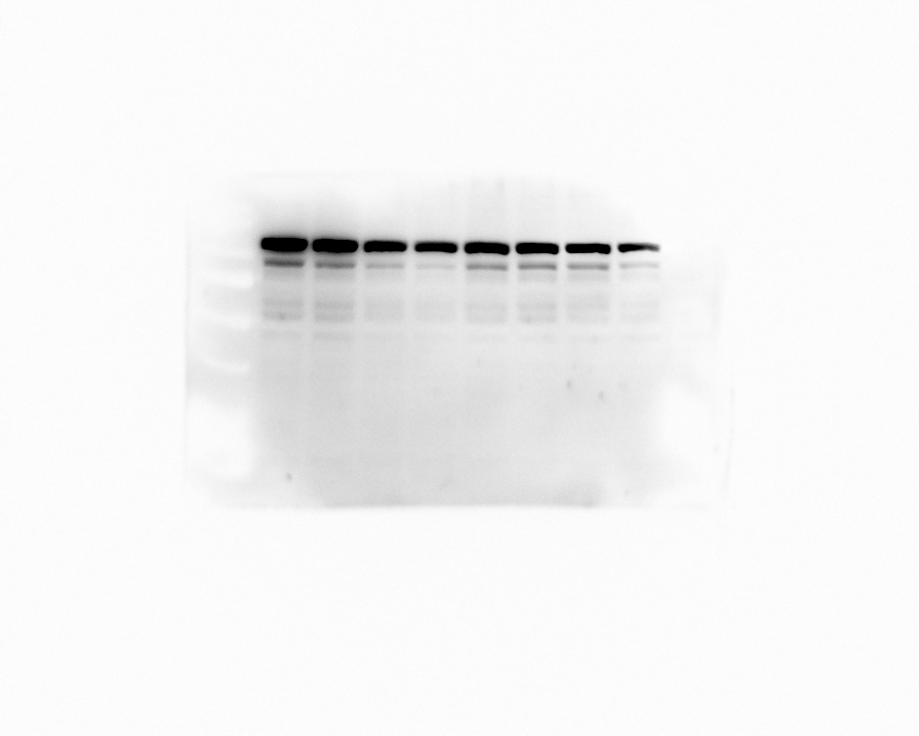

Supplement: Figure 6—source data 1. [file elife-96353-fig6-data1.zip › fig. 6P/input anti-myc_1s_opt-0006.tif]

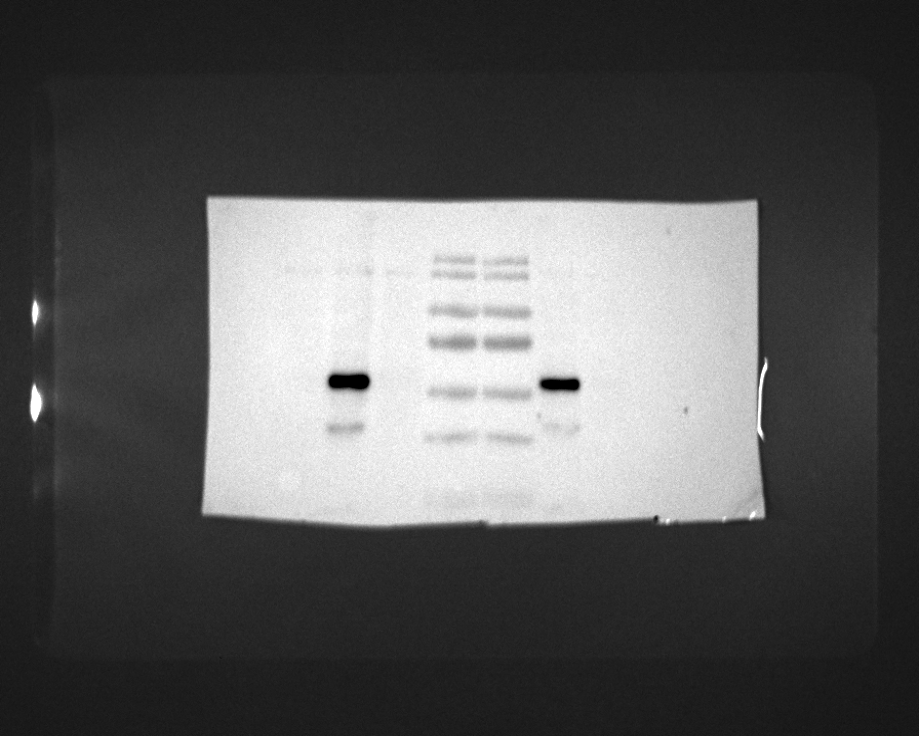

Supplement: Figure 6—source data 1. [file elife-96353-fig6-data1.zip › fig. 6P/IP anti-flag.tif]

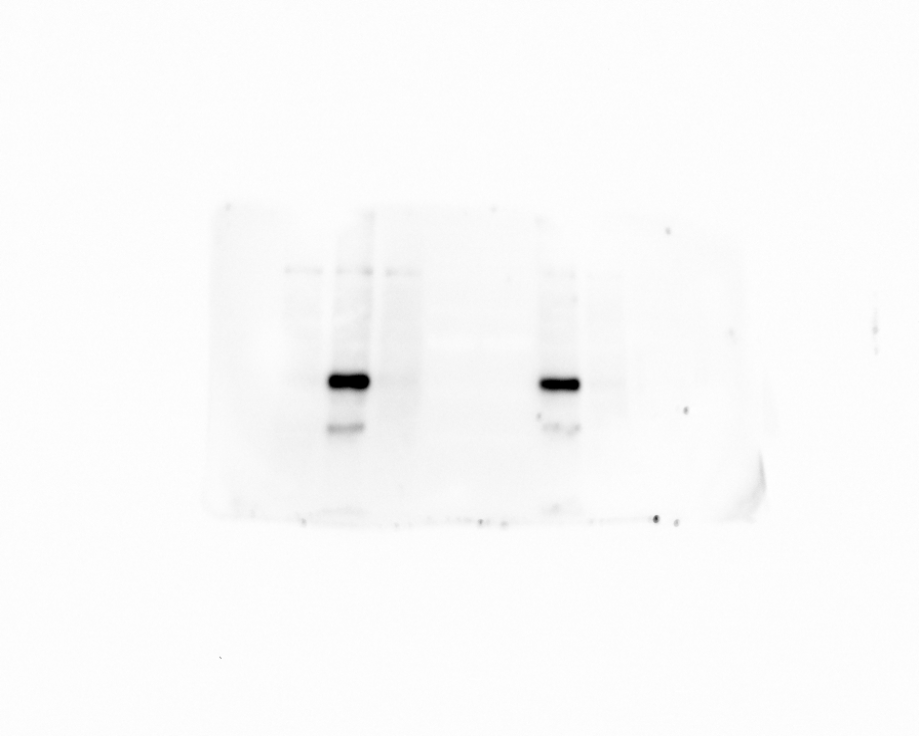

Supplement: Figure 6—source data 1. [file elife-96353-fig6-data1.zip › fig. 6P/IP anti-flag_30s_opt-0004.tif]

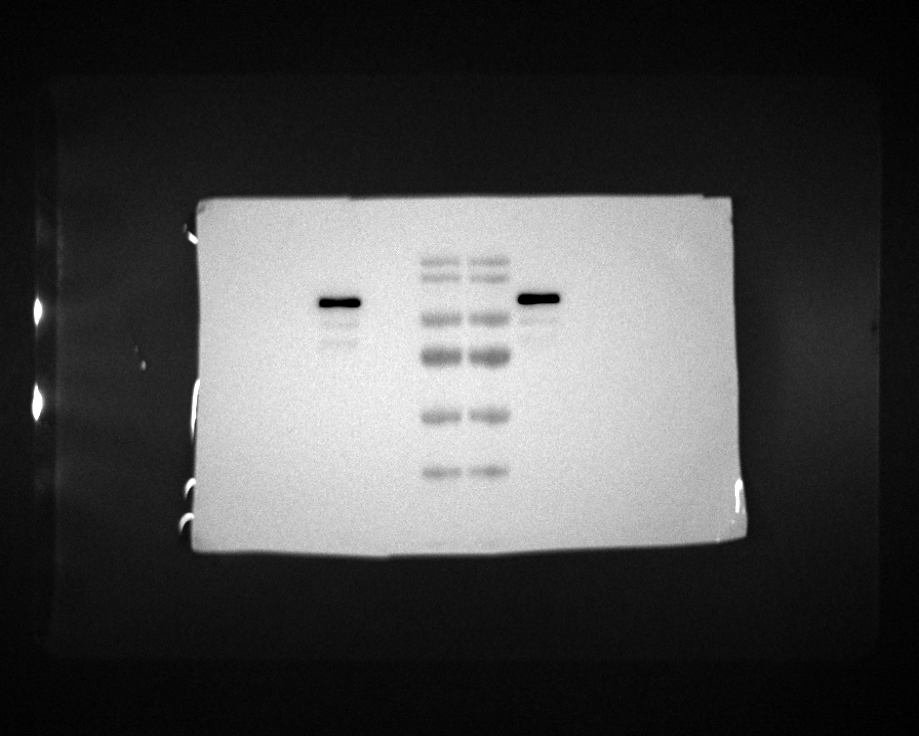

Supplement: Figure 6—source data 1. [file elife-96353-fig6-data1.zip › fig. 6P/IP anti-myc.tif]

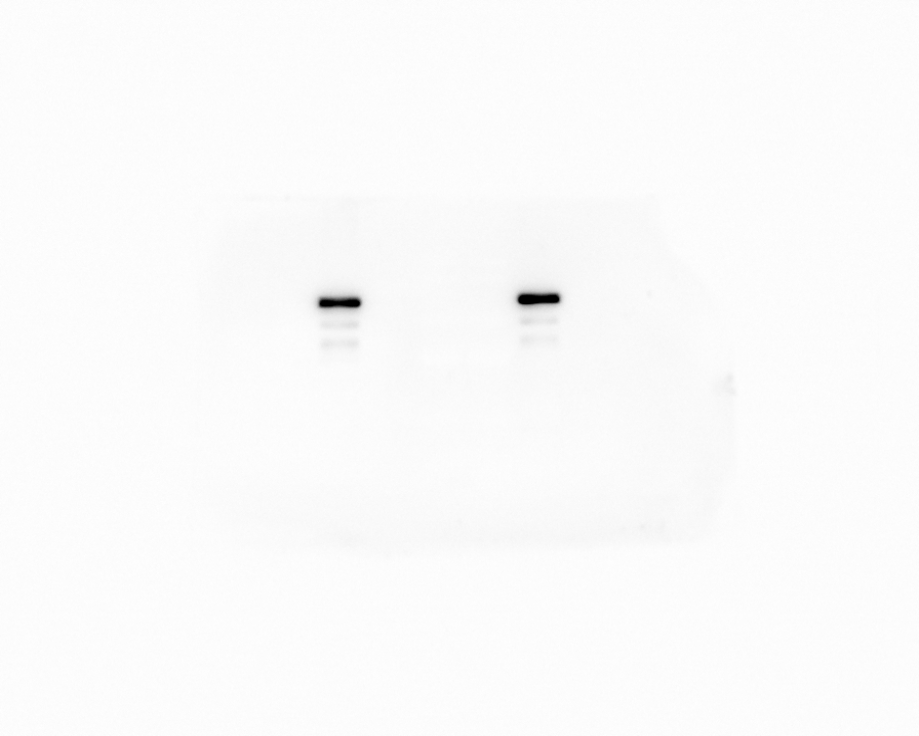

Supplement: Figure 6—source data 1. [file elife-96353-fig6-data1.zip › fig. 6P/IP anti-myc_1s_opt-0005.tif]

Figure 6P

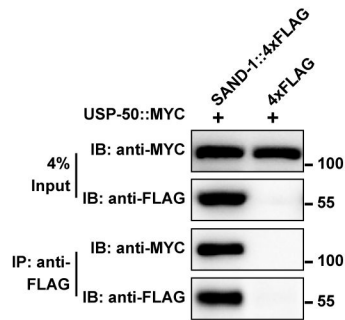

IB: anti-MYC

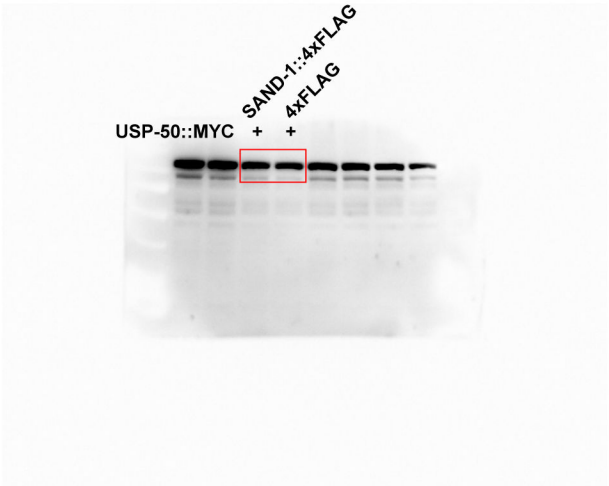

IB: anti-FLAG

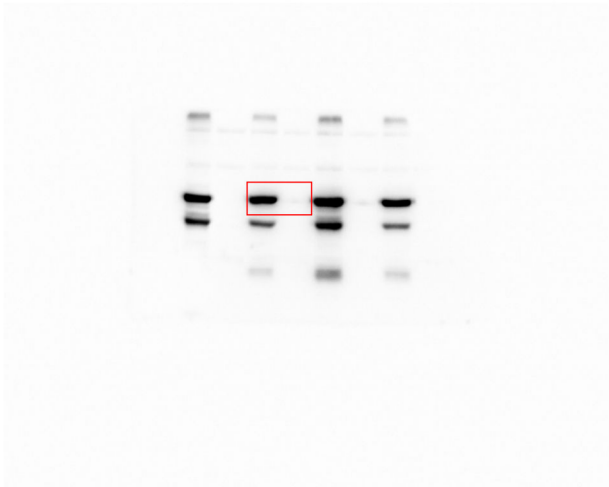

IB: anti-MYC

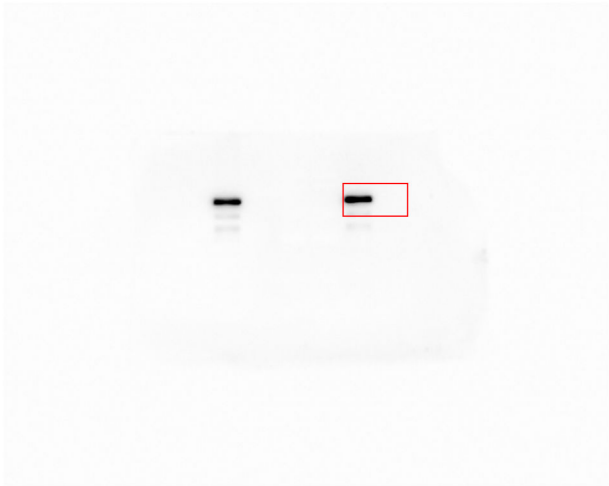

IB: anti-FLAG

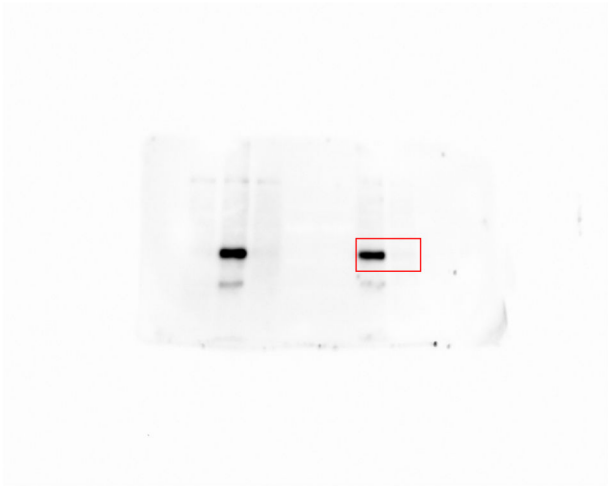

Supplement: Figure 6—source data 2. [file elife-96353-fig6-data2.pdf]

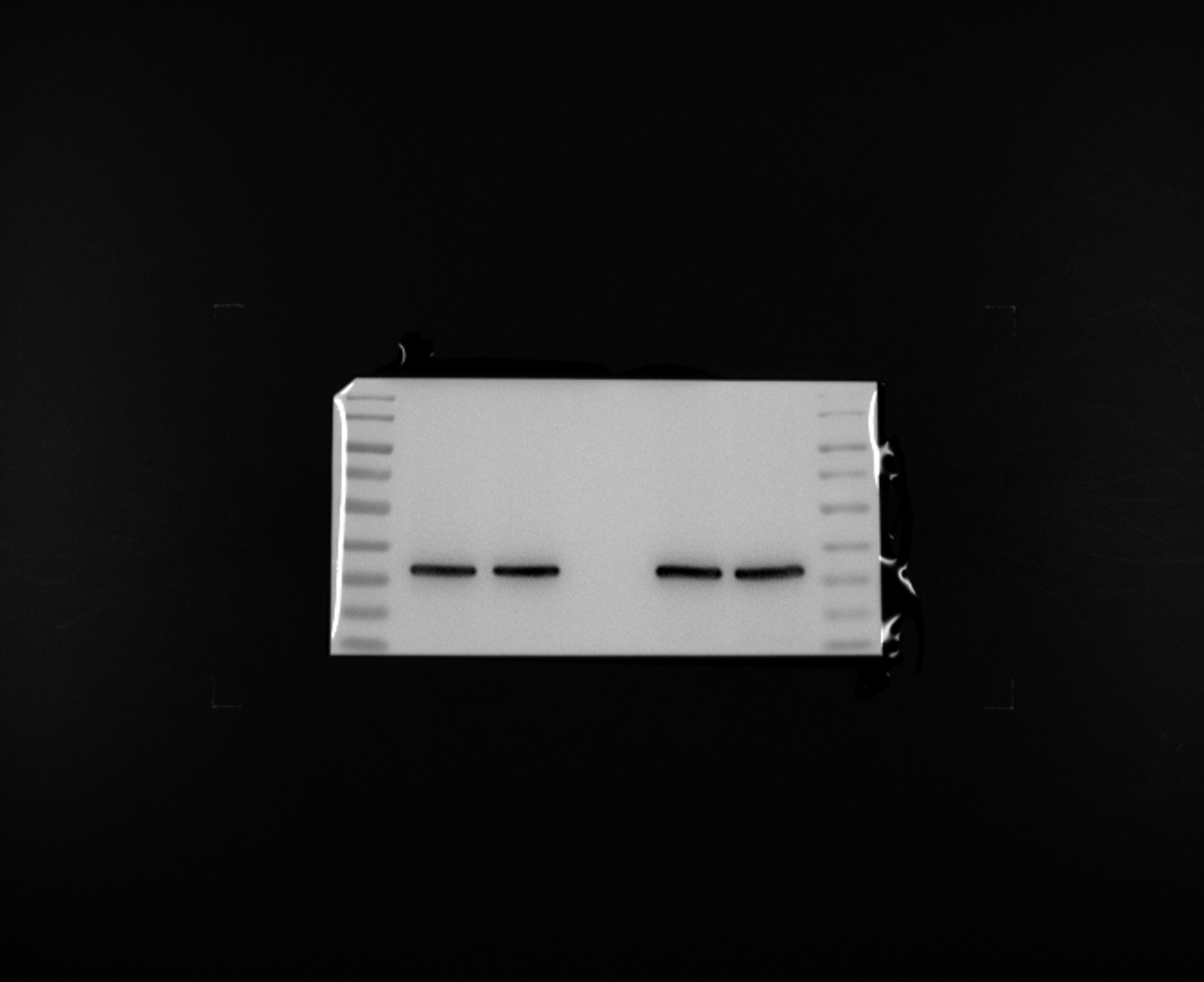

Supplement: Figure 6—figure supplement 1—source data 1. [file elife-96353-fig6-figsupp1-data1.zip › fig. 6-S1B, E/GAPDH-1.5S+M(fig6-s1).Tif]

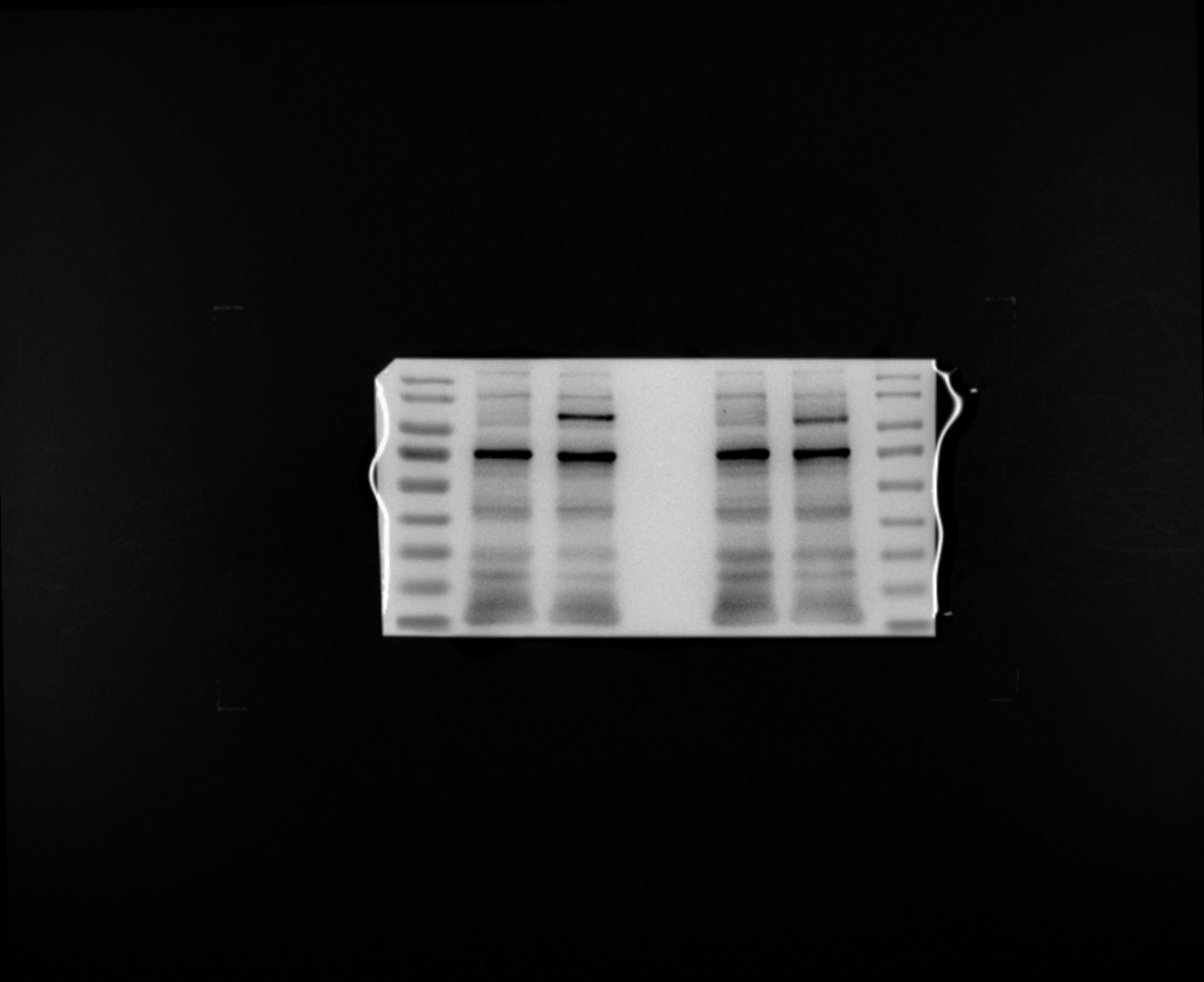

Supplement: Figure 6—figure supplement 1—source data 1. [file elife-96353-fig6-figsupp1-data1.zip › fig. 6-S1B, E/GFP-15S+M-1(fig6-s1).tif]

Figure 6-S1B

Figure 6-S1E

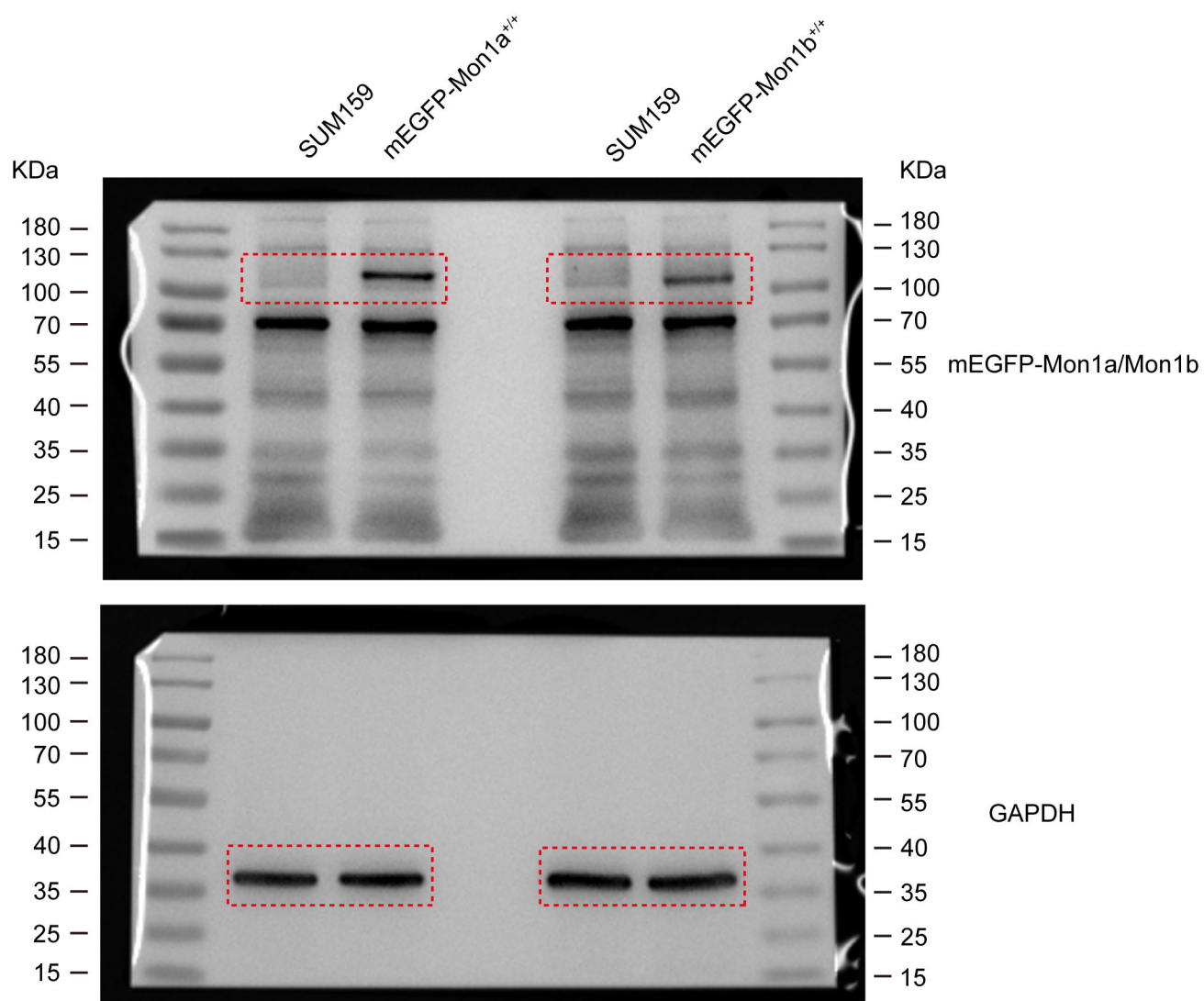

Supplement: Figure 6—figure supplement 1—source data 2. [file elife-96353-fig6-figsupp1-data2.pdf]
