# Supplementary material for: Spatiotemporal recruitment of the ubiquitin-specific protease USP8 directs endosome maturation
Source: eLife. 2024 Nov 22;13:RP96353. doi: 10.7554/eLife.96353 (PMC11584181; doi:10.7554/eLife.96353)
Supplement: Figure 4—source data 2. [file elife-96353-fig4-data2.pdf]

Figure 4A

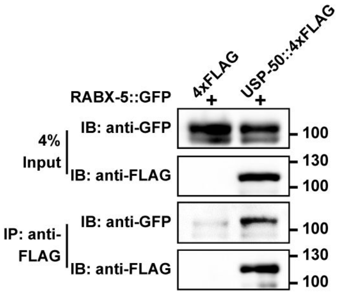
4xFLAG
USP-50::4xFLAG
RABX-5::GFP
+
+
4% Input
IB: anti-GFP
IB: anti-FLAG
IP: anti-FLAG
IB: anti-GFP
IB: anti-FLAG
100
130
100
100
130
100

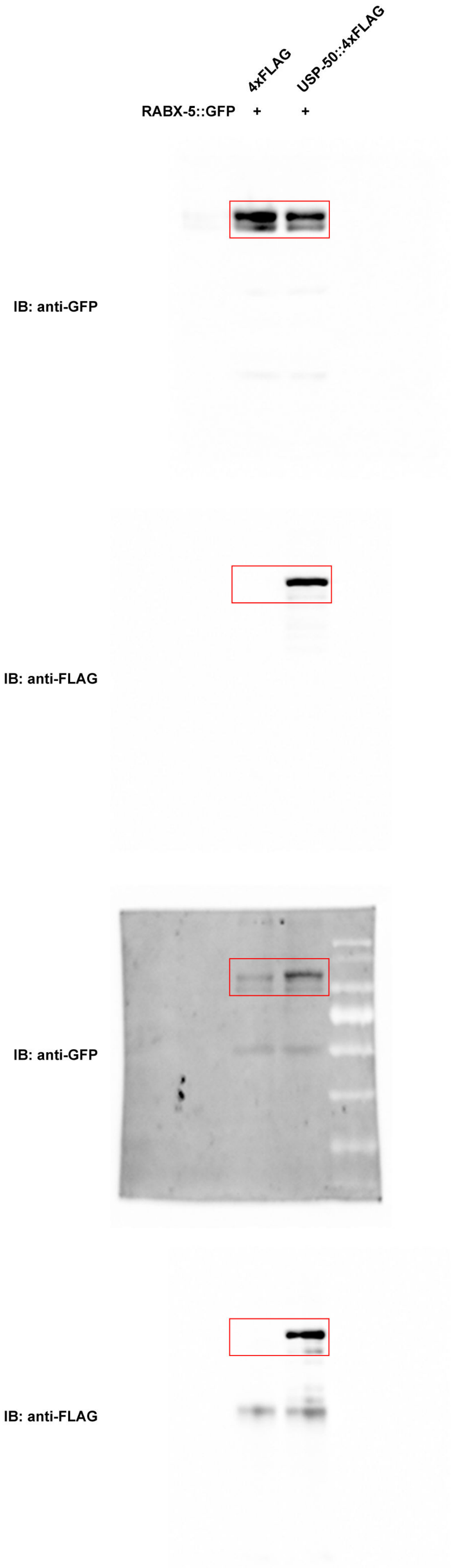
4xFLAG
USP-50::4xFLAG
RABX-5::GFP
+
+
IB: anti-GFP
IB: anti-FLAG
IB: anti-GFP
IB: anti-FLAG
